# Brain p3‐Alcβ peptide restores neuronal viability impaired by Alzheimer's amyloid β‐peptide

**DOI:** 10.15252/emmm.202217052

**Published:** 2023-03-30

**Authors:** Saori Hata, Haruka Saito, Takeharu Kakiuchi, Dai Fukumoto, Shigeyuki Yamamoto, Kensaku Kasuga, Ayano Kimura, Koichi Moteki, Ruriko Abe, Shungo Adachi, Shoich Kinoshita, Kumiko Yoshizawa‐Kumagaye, Hideki Nishio, Takashi Saito, Takaomi C Saido, Tohru Yamamoto, Masaki Nishimura, Hidenori Taru, Yuriko Sobu, Hiroyuki Ohba, Shingo Nishiyama, Norihiro Harada, Takeshi Ikeuchi, Hideo Tsukada, Yasuomi Ouchi, Toshiharu Suzuki

**Affiliations:** ^1^ Laboratory of Neuroscience, Graduate School of Pharmaceutical Sciences Hokkaido University Sapporo Japan; ^2^ Biomedical Research Institute National Institute of Advanced Industrial Science and Technology (AIST) Tsukuba Japan; ^3^ Advanced Prevention and Research Laboratory for Dementia, Graduate School of Pharmaceutical Sciences Hokkaido University Sapporo Japan; ^4^ Central Research Laboratory, Hamamatsu Photonics K.K. Hamamatsu Japan; ^5^ Molecular Genetics Niigata University Brain Research Institute Nigata Japan; ^6^ Cellular and Molecular Biotechnology Research Institute National Institute of Advanced Industrial Science and Technology (AIST) Tokyo Japan; ^7^ Peptide Institute, Inc. Ibaraki Japan; ^8^ Laboratory for Proteolytic Neuroscience RIKEN Center for Brain Science Institute Wako Japan; ^9^ Department of Neurocognitive Science, Institute of Brain Science Nagoya City University Graduate School of Medical Sciences Nagoya Japan; ^10^ Department of Molecular Neurobiology, Factory of Medicine Kagawa University Takamatsu Japan; ^11^ Molecular Neuroscience Research Center Shiga University of Medical Science Shiga Japan; ^12^ Department of Biofunctional Imaging, Preeminent Medical Education & Research Center Hamamatsu University School of Medicine Hamamatsu Japan

**Keywords:** AD therapy, alcadein, Alzheimer's disease (AD), mitochondria, PET imaging, Neuroscience

## Abstract

We propose a new therapeutic strategy for Alzheimer's disease (AD). Brain peptide p3‐Alcβ37 is generated from the neuronal protein alcadein β through cleavage of γ‐secretase, similar to the generation of amyloid β (Aβ) derived from Aβ‐protein precursor/APP. Neurotoxicity by Aβ oligomers (Aβo) is the prime cause prior to the loss of brain function in AD. We found that p3‐Alcβ37 and its shorter peptide p3‐Alcβ9‐19 enhanced the mitochondrial activity of neurons and protected neurons against Aβo‐induced toxicity. This is due to the suppression of the Aβo‐mediated excessive Ca^2+^ influx into neurons by p3‐Alcβ. Successful transfer of p3‐Alcβ9‐19 into the brain following peripheral administration improved the mitochondrial viability in the brain of AD mice model, in which the mitochondrial activity is attenuated by increasing the neurotoxic human Aβ42 burden, as revealed through brain PET imaging to monitor mitochondrial function. Because mitochondrial dysfunction is common in the brain of AD patients alongside increased Aβ and reduced p3‐Alcβ37 levels, the administration of p3‐Alcβ9‐19 may be a promising treatment for restoring, protecting, and promoting brain functions in patients with AD.

## Graphical abstract

This study presents a novel therapeutic approach for treating Alzheimer's disease (AD) by utilizing a small peptide derived from the brain peptide p3‐Alcβ37.

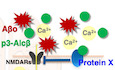

**The paper explained**

**Problem**

Neuronal p3‐Alcβ peptides are generated through proteolytic cleavage of the precursor protein Alcadein β/Alcβ (also known as calsyntenin‐3/Clstn3) by α‐ and γ‐secretases, while neurotoxic amyloid β (Aβ) is derived from the sequential cleavage of Aβ‐protein precursor/APP by β‐ and γ‐secretases. Aβ oligomers (Aβo) are considered highly neurotoxic and the main culprit playing a role in the pathophysiology of Alzheimer's disease (AD). AD is the most common, incurable neurodegenerative disease in elderly subjects with dementia. A series of our experiments so far have shown that the p3‐Alcβ level in CSF decreases with age and with Aβ accumulation increasing, which causes to reduce the p3‐Alcβ expression in the brain. Moreover, the CSF p3‐Alcβ level significantly decreases in AD patients compared with that in age‐matched nondemented subjects. Intriguingly, subjects carrying presenilin gene mutations of hereditary and familial AD show a lower p3‐Alcβ level than noncarriers in the family. These lines of evidence suggest that p3‐Alcβ may associate strongly with AD pathophysiology, but the exact functions of p3‐Alcβ remain unclear.

**Results**

As a proof‐of‐concept, we asked about the effects of p3‐Alcβ on neurons. In contrast to the neurotoxic effect of Aβo, the p3‐Alcβ increased neuronal viability and protected neurons against the neurotoxicity of Aβo. We identified a functionally active shorter peptide p3‐Alcβ9‐19, composed of 11‐amino acid sequence of endogenous p3‐Alcβ37. Interestingly, p3‐Alcβ37 and p3‐Alcβ9‐19 inhibited anomalous Aβo‐induced Ca^2+^ influx in neurons and restored neuronal viability by keeping intracellular Ca^2+^ homeostasis. Moreover, peripheral administration of p3‐Alcβ9‐19, which shows an excellent BBB crossing property, restored neuronal viability impaired by increasing Aβ burden in the brain of the AD mouse model.

**Impact**

No curative drugs for AD exist yet. Anti‐AD immunotherapeutic drugs being developed are confronting difficulties in their effectiveness, safety, cost performance, and so on. This reality gives us to reconsider a different therapeutic strategy other than drugs targeting Aβ. Here, we showed that a short peptide p3‐Alcβ9‐19 is transferred into the brain satisfactorily by peripheral administration, which allowed us to develop the transdermal administration procedure with p3‐Alcβ9‐19 pharmaceutical formulation. The p3‐Alcβ is a brain endogenous peptide and is detected in blood in humans, guaranteeing the safety and regular evaluation of its level in patients. In fact, no toxicity was observed in a series of rodent and monkey experiments. The short peptide is much less expensive and stable at 4°C for years. Novel therapy with p3‐Alcβ9‐19 pharmaceutical formulation is expected as a promising drug for AD patients.

## Introduction

Alzheimer's disease (AD) is an incurable neurodegenerative disorder that causes progressive dementia in aged subjects. More than 50 million people live with dementia worldwide and AD accounts for 60–80% of these cases (World Alzheimer's Report, 2018). The generation and accumulation of neurotoxic amyloid β (Aβ) protein, a pathogenic hallmark in the AD brain, are believed to be the primary cause of neurodegeneration, resulting in cognitive deficits and memory loss (McLean *et al*, 1999; Hardy & Selkoe, 2002). In neurons, Aβ is generated from Aβ‐protein precursor (APP), a single membrane‐spanning protein, via intramembrane proteolysis of γ‐secretase after the shedding of the APP extracellular/luminal region by β‐secretase/BACE1 (De Strooper *et al*, 1998; Vassar *et al*, 1999; Thinakaran & Koo, 2008).

Alcadeins (Alcs)/calsyntenins (Clstns) are a family of type I transmembrane proteins expressed in neurons. This family includes alcadein α (Alcα/Clstn1), β (Alcβ/Clstn3), and γ (Alcγ/Clstn2), which are encoded by separate genes (Hintsch *et al*, 2002; Araki *et al*, 2003). Alcα/Clstn1 is a cargo receptor of kinesin‐1 (Konecna *et al*, 2006; Araki *et al*, 2007; Kawano *et al*, 2012; Vagnoni *et al*, 2012; Sobu *et al*, 2017) and regulates secretory pathways (Ludwig *et al*, 2009; Takei *et al*, 2015; Sobu *et al*, 2017). Alcβ/Clstn3 acts as a synaptic adhesion molecule and regulates synapse formation (Pettem *et al*, 2013; Lu *et al*, 2014; Um *et al*, 2014). Alcγ/Clstn2 is involved in memory performance and cognition (Boraxbekk *et al*, 2015; Lipina *et al*, 2016). Following primary cleavage by α‐secretase (ADAM10/ADAM17), Alcs undergo intramembrane proteolysis by γ‐secretase and secrete the short peptides p3‐Alcs, similar to APP (Araki *et al*, 2004; Hata *et al*, 2009). The precise functions of p3‐Alcs are unknown, whereas Aβ generated from APP forms neurotoxic oligomers (Aβo) and triggers pathogenic processes in AD (Benilova *et al*, 2012). Among the p3‐Alcs secreted, the major peptides p3‐Alcα35 (of Alcα) and p3‐Alcβ37 (of Alcβ) are found in human cerebrospinal fluid (CSF) at levels similar to that of Aβ40 (Hata *et al*, 2009, 2011). Moreover, the levels of p3‐Alcβ in CSF are notably reduced in AD (Hata *et al*, 2019). These lines of evidence suggest that p3‐Alcβ is closely associated with AD pathophysiology. Here, we analyzed the functions of p3‐Alcβ in neurons suffering from neurotoxic Aβo *in vitro* and in the brain of an AD mouse model *in vivo*. Our results show a novel neuroprotective function of p3‐Alcβ, which allowed us to propose that the administration of p3‐Alcβ peptides to individuals in the AD continuum protects neurons by increasing p3‐Alcβ levels in the brain and consequently is effective in slowing the progression of the disease.

## Results

### Human CSF p3‐Alcβ37 level decreases significantly in the early stage of AD patients

**Figure 1:**
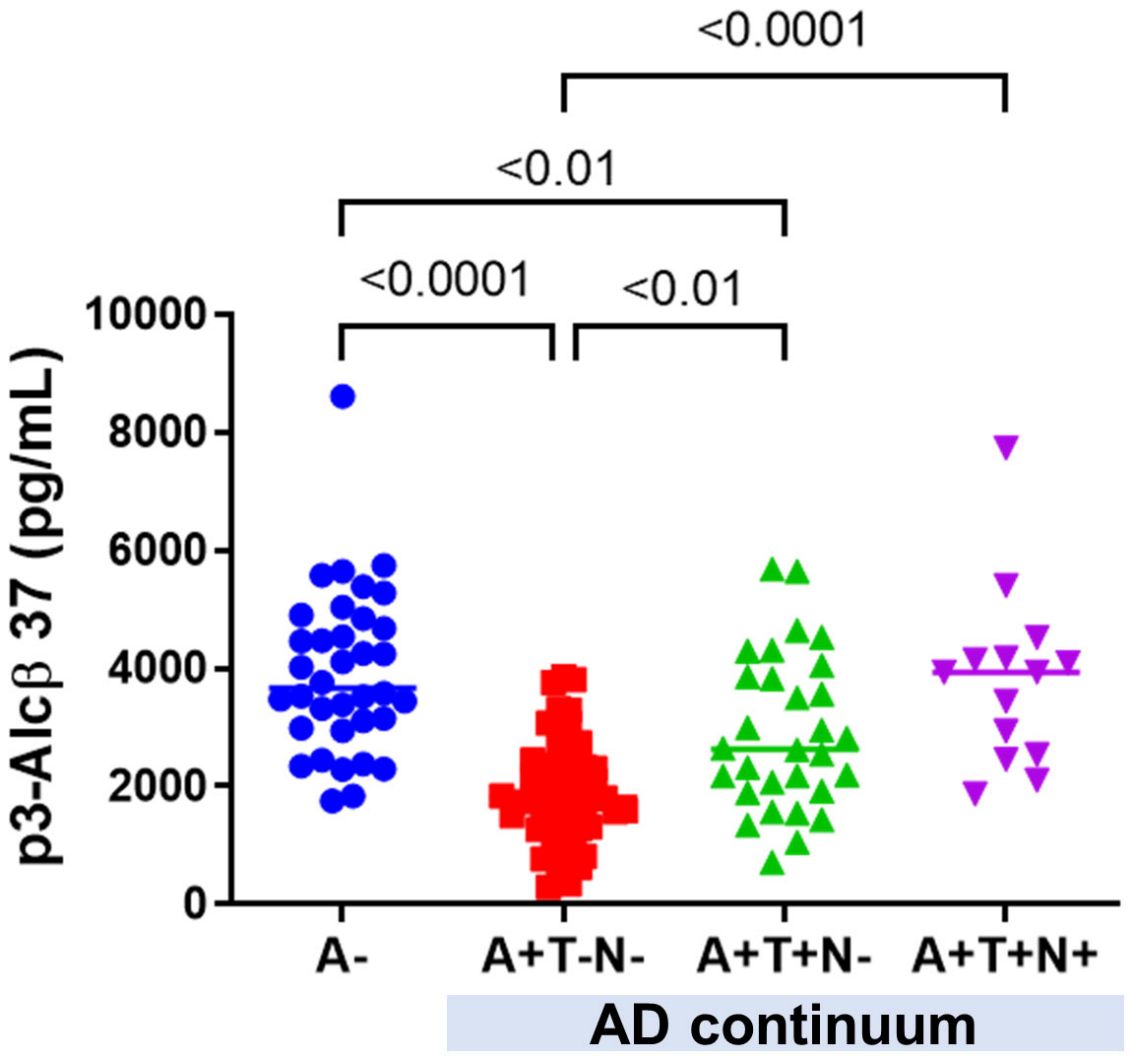
p3‐Alcβ37 levels in the CSF of subjects along the AD continuum CSF p3‐Alcβ37 values in subjects who were categorized along the AD continuum (*n* = 131). Cut‐off values are 359.6 pg/ml for Aβ42 (A+ indicates < 359.6 pg/ml), 30.6 pg/ml for p‐tau (T+ indicates > 30.6 pg/ml), and 105.3 pg/ml for t‐tau (N+ indicates > 105.3 pg/ml), respectively. Statistical significance was determined by a one‐way ANOVA followed by the Tukey's multiple comparisons test (mean ± SEM; A−: *n* = 36, A+T−N−: *n* = 51, A+T+N−: *n* = 30, A+T+N+: *n* = 14), and significant *P*‐values (*P* < 0.01, *P* < 0.0001) are indicated on the graph. The summary of subjects and results is shown in Appendix Table S1. Data information: Experimental numbers indicate biological replicates (subject numbers). Detailed information including the statistical summary is described in Dataset EV1. Source data are available online for this figure.

We previously reported that the p3‐Alcβ levels in CSF significantly reduced in patients who were clinically diagnosed with AD (Hata *et al*, 2019). To further investigate the relationship between AD progression and p3‐Alcβ levels, we examined p3‐Alcβ37 levels in CSF of patients who had been classified into AD biomarker categories (Jack *et al*, 2018). Subjects along the AD continuum (*n* = 131) were categorized according to biomarker‐based criteria (Kasuga *et al*, 2022), and CSF p3‐Alcβ37 values were quantified (Fig 1) (Appendix Table S1). Interestingly, the level of p3‐Alcβ37 in AD patients at an early stage (A+T−N−) was found to be significantly lower (*P* < 0.0001) than that in subjects (A−). The finding raises a hypothesis that a decrease in the level of brain p3‐Alcβ is pathophysiologically important in the development of AD. Hence, we proceeded to analyze the molecular characterization of p3‐Alcβ and its physiological functions on neurons and the brain milieu.

### p3‐Alcβ increases neuronal viability and protects neurons against Aβ42 oligomer‐induced toxicity

**Figure 2:**
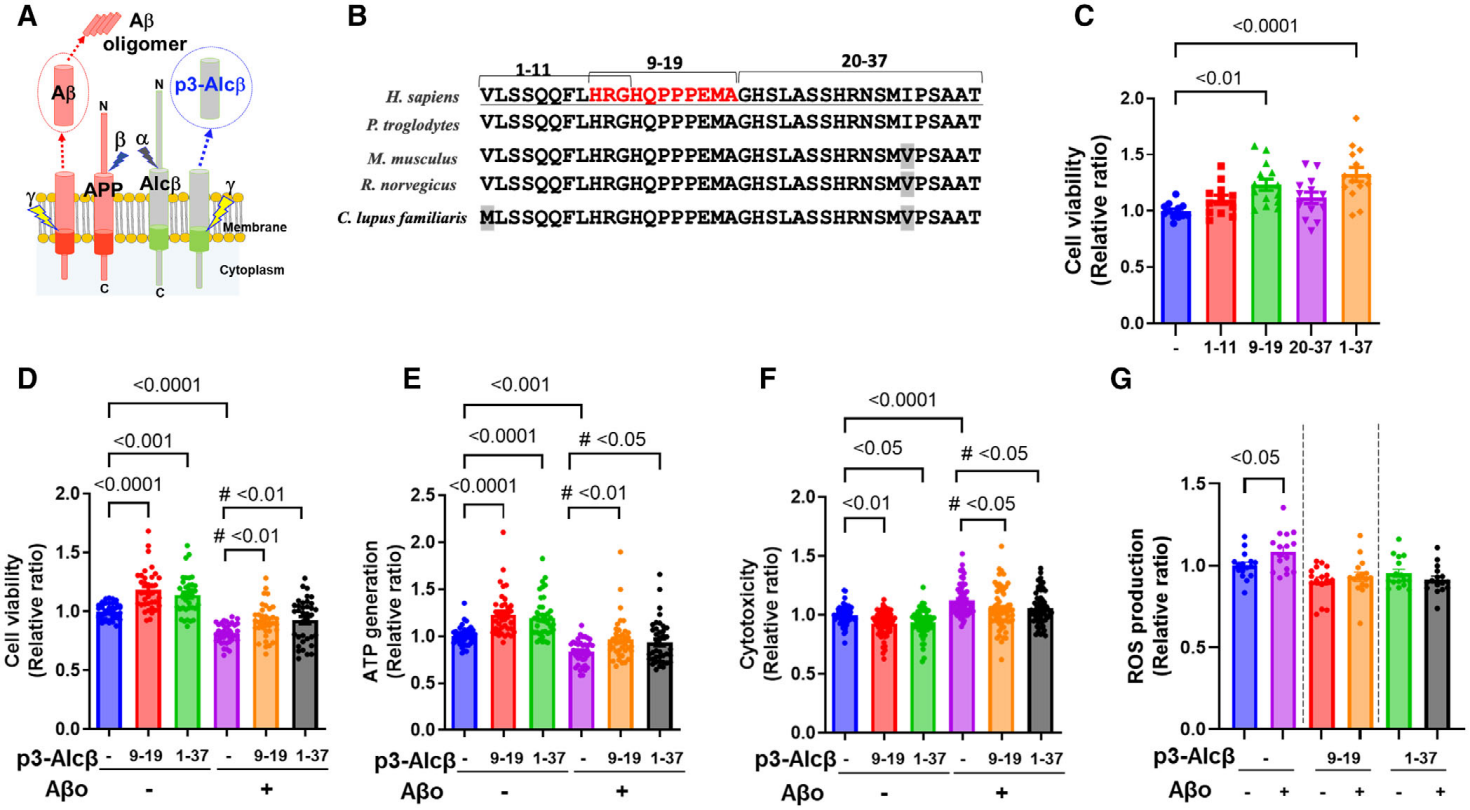
P3‐Alcβ peptides increase neuronal viability and protect neurons against Aβ42 oligomer‐induced toxicity ASchematic diagram of Aβ and p3‐Alcβ generation from their precursors APP and Alcβ. The cleavage site by secretases: α, α‐secretase; β, β‐secretase; γ, γ‐secretase.BThe amino acid sequence of p3‐Alcβ37 in humans, chimpanzees, mice, rats, and dogs. The amino acid sequence of p3‐Alcβ9‐19 is shown in red letters for humans, and gray boxes indicate an amino acid that is different in nonprimates.CEffect of p3‐Alcβ37 and its partial peptides on the viability of mouse primary cultured neurons. Wild‐type (WT) neurons (div 15–20) were incubated for 24 h in the presence (10 μM) or absence (−) of p3‐Alcβ1‐11, p3‐Alcβ9‐19, p3‐Alcβ20‐37, and p3‐Alcβ1‐37. Neuronal viability was evaluated with MTT assays and expressed relative to that of cells cultured in the absence of peptides (assigned a value of 1.0). Statistical analysis was performed using a one‐way ANOVA, followed by the Dunnett's multiple comparisons test (mean ± SEM; *n* = 10–12), and significant *P*‐values (*P* < 0.01, *P* < 0.0001) are indicated on the graph.D–FEffect of p3‐Alcβ on the neuronal toxicity of Aβ42 oligomers. WT neurons (div 15–20) were incubated for 24 h in the presence (10 μM) or absence (−) of p3‐Alcβ9‐19 and p3‐Alcβ1‐37 with (+) or without (−) Aβ42 oligomers (Aβo, 2.5 μM). Neuronal viability was evaluated by MTT (D), ATP generation (E), and LDH release (F) assays and expressed relative to neurons cultured in the absence of p3‐Alcβ and Aβo (assigned a value of 1.0). Statistical significance was determined by Dunnett's multiple comparisons tests (mean ± SEM; MTT: *n* = 36, ATP: *n* = 40, LDH: *n* = 60). The significant *P*‐values (*P* < 0.05, *P* < 0.01, *P* < 0.001, *P* < 0.0001) versus cells incubated in the absence (−) of p3‐Alcβ and Aβo are indicated in the graphs. The significant *P*‐values (^#^ *P* < 0.05, ^#^ *P* < 0.01) versus cells incubated in the presence (+) of Aβo and in the absence (−) of p3‐Alcβ are indicated in the graphs.GEffect of Aβ42 oligomers (Aβo) and p3‐Alcβ peptides on the generation of reactive oxygen species (ROS). WT neurons (div 15–17) were incubated for 24 h with (+) or without (−) Aβo (2.5 μM) and in the presence (+) or absence (−) of p3‐Alcβ (10 μM). ROS generation was assessed and expressed relative to that of neurons incubated in the absence (−) of Αβo, which was assigned a value of 1.0. Statistical significance between samples with or without Aβo was evaluated with a Student's *t*‐test (mean ± SEM; *n* = 10), and the significant *P*‐value (*P* < 0.05) is indicated on the graph. Data information: Experimental numbers indicate biological replicates. Detailed information including the statistical summary is described in Dataset EV1. Source data are available online for this figure.

**Figure EV1:**
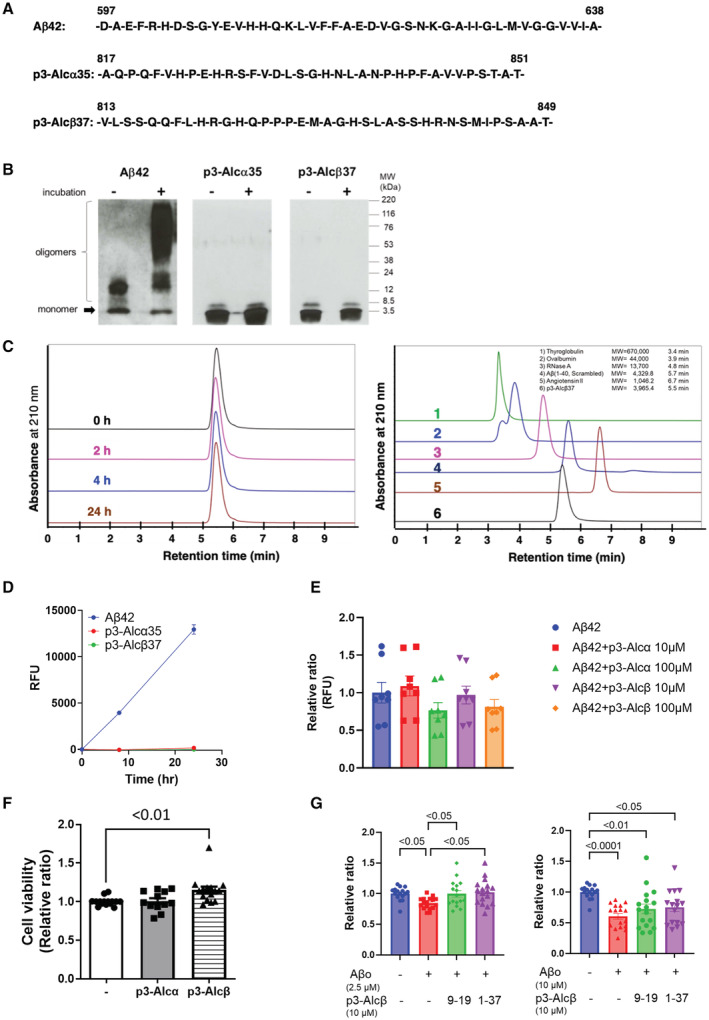
Nonaggregative property of p3‐Alc peptides and the effect of p3‐Alcβ on the viability of mouse primary cultured neurons The amino acid sequences of human Aβ42, p3‐Alcα35, and p3‐Alcβ37 (amino acid numbers of Aβ42 are for the APP695 isoform).p3‐Alc peptides do not aggregate. Synthetic Aβ42, p3‐Alcα35, and p3‐Alcβ37 peptides (10 μM each) were solubilized in PBS with (+) or without (−) incubation at 37°C for 24 h. The peptides were immunoblotted using anti‐Aβ 82E1 (left), anti‐p3‐Alcα UT135 (middle), and anti‐p3‐Alcβ #854 (right) antibodies. Protein size markers are shown (kDa).p3‐Alcβ37 does not form oligomers in PBS. Synthetic p3‐Alcβ37 was dissolved in PBS (50 μM) and then incubated at 37°C for the indicated time (0, 2, 4, 24 h), and aliquots (5 μl) were subjected to size‐exclusion chromatography (left panel) along with molecular size marker proteins (right). The retention time of marker proteins is indicated on the graph on the right.Monitoring of peptide aggregation with Thioflavin T fluorescence. Indicated peptide solutions (10 μl of 10 μM) were incubated for the indicated time (h) at 37°C. Thioflavin T was then added to the peptide solutions, and the fluorescence was measured (Ex. 430 nm/Em. 485 nm). The fluorescence intensity is represented as a relative fluorescent unit (RFU) (mean ± SEM; *n* = 8).Aβ42 aggregation in the presence of p3‐Alc peptides. Aliquots (10 μl) of Aβ42 solution (20 μM) were combined with the aliquots (10 μl) of p3‐Alcα35 or p3‐Alcβ37 solutions (0, 20, 200 μM). The assay solutions were incubated for 12 h at 37°C and Thioflavin T was added to measure the fluorescence (Ex 430 nm/Em 485 nm). The fluorescence intensity (RFU) is expressed relative to that of the sample in the absence of p3‐Alc peptides (assigned a value of 1.0). Statistical significance was determined by a one‐way ANOVA followed by the Tukey's multiple comparisons test (mean ± SEM; *n* = 6).Effect of p3‐Alcα35 and p3‐Alcβ37 on the viability of mouse primary cultured neurons. Wild‐type neurons (div 15–20) were incubated for 24 h in the presence (10 μM) or absence (−) of p3‐Alcα35 and p3‐Alcβ37. Neuronal viability was evaluated using MTT assays and is expressed relative to that of cells cultured in the absence of peptides (assigned a value of 1.0). Statistical analysis was performed using a one‐way ANOVA, followed by the Dunnett's multiple comparisons test (mean ± SEM; *n* = 10), and the significant *P*‐value (*P* < 0.01) is indicated on the graph.Aβ42 oligomer‐induced neurotoxicity and restoration of neuronal viability by p3‐Alcβ9‐19 and p3‐Alcβ37. Wild‐type mouse neurons (DIV 16–17) were incubated for 24 h with (+, 2.5 μM (left) and 10 μM (right)) or without (−) Aβ42 oligomers (Aβo) in the presence (9–19, p3‐Alcβ9‐19; 1–37, p3‐Alcβ37) or absence (−) of p3‐Alcβ peptide (10 μM). Neuronal viability was evaluated with MTT assays and expressed relative to that of cells cultured in the absence of peptides (assigned a value of 1.0). Statistical analysis was performed using a one‐way ANOVA, followed by the Tukey's multiple comparisons test (mean ± SEM; *n* = 16–17), and the significant *P*‐values (*P* < 0.05, *P* < 0.01, *P* < 0.0001) are indicated on the graph. Data information: Experimental numbers indicate biological replicates. Detailed information including the statistical summary is described in Dataset EV2. Source data are available online for this figure.

The p3‐Alcα and p3‐Alcβ peptides are generated through cleavage processing of their precursor molecules, Alcα and Alcβ, by α‐ and γ‐secretases, whereas Aβ is derived from the sequential cleavage of APP by β‐ and γ‐secretases (Fig 2A) (Hata *et al*, 2009). We first examined whether the p3‐Alc peptides would possess aggregation properties as does Aβ. Synthetic peptides, Aβ42, p3‐Alcα35, and p3‐Alcβ37 (the amino acid sequences of the peptides are shown in Fig EV1A), were dissolved in PBS and incubated at 37°C for 24 h (+) or not (−), and the samples were analyzed by immunoblotting with specific antibodies (Fig EV1B). Unlike the aggregation/oligomer formation of Aβ42, p3‐Alcα35, and p3‐Alcβ37 did not aggregate. The oligomerization of p3‐Alcβ37 was examined by size‐exclusion chromatography (Fig EV1C). The p3‐Alcβ37 peptide (50 μM in PBS), which was incubated at 37°C for 24 h, was eluted with a monomer retention time of 5.5 min and yielded an identical recovery of peptides at the indicated time points of 0, 2, 4, and 24 h. These findings indicate that p3‐Alcβ37 does not form oligomers that are labile to SDS electrophoresis. Aβ42 aggregation is easily monitored with the Thioflavin T fluorescence assay (Walsh *et al*, 1999). The fluorescence intensity in the presence of Aβ42 (10 μM) increased linearly with the incubation time, whereas the fluorescence intensities in the presence of p3‐Alcα35 (10 μM) and p3‐Alcβ37 (10 μM) were below the threshold and did not increase until the incubation time point of 24 h (Fig EV1D). These results indicate that p3‐Alc peptides are not prone to aggregation. Furthermore, in an *in vitro* study with Thioflavin T, p3‐Alcβ did not suppress Aβ42 aggregation effectively and specifically (Fig EV1E). Hence, it is likely that p3‐Alcβ is a nonaggregation peptide that does not act directly on Aβ aggregation.

Next, we assessed the effects of p3‐Alc on neurons and whether p3‐Alc peptides are neurotoxic in the same way as Aβ. Mouse primary neurons were cultured for 24 h in the presence of p3‐Alcα35, or p3‐Alcβ37, and their cell viability was then examined with MTT assays (Fig EV1F). Interestingly, neurons showed significantly better viability (*P* < 0.01) in the presence of p3‐Alcβ37 than in the absence of p3‐Alcβ37. This effect was not observed in presence of p3‐Alcα35, and the amino acid sequence of p3‐Alcβ37 is distinct from that of p3‐Alcα35 (Fig EV1A) (Hata *et al*, 2009), indicating that the effect is p3‐Alcβ‐specific.

The amino acid sequence of p3‐Alcβ is highly conserved across five mammalian species (Fig 2B), thus we asked which region serves the increase in neuronal viability. MTT assays to evaluate the effects of several peptides, namely p3‐Alcβ1‐11, p3‐Alcβ9‐19, p3‐Alcβ20‐37 and the original p3‐Alcβ1‐37 (i.e., p3‐Alcβ37), were performed (Fig 2C). Similar to p3‐Alcβ37 (*P* < 0.0001), neurons cultured with p3‐Alcβ9‐19 showed significantly higher viability than neurons cultured in the absence (−) of peptides (*P* < 0.01). The functionally active 11‐amino acid sequence of p3‐Alcβ9‐19 was completely identical across the mammalian species examined (red letters, Fig 2B). Together with MTT assays, the functional properties of p3‐Alcβ37 and p3‐Alcβ9‐19 (Aβo (−)) were further assessed in mouse primary cultured neurons by measuring intracellular ATP levels, an indicator of mitochondrial energy production, and the levels of lactate dehydrogenase (LDH) in culture medium, an indicator of cytotoxicity‐induced membrane impairment (Fig 2D–F). p3‐Alcβ37 and p3‐Alcβ9‐19 significantly increased neuronal viability (*P* < 0.001, *P* < 0.0001) (Fig 2D), as shown by an increase in intracellular ATP generation (*P* < 0.0001) (Fig 2E) and a significant decrease in LDH release into the culture medium (*P* < 0.05, *P* < 0.01) (Fig 2F).

Interestingly, p3‐Alcβ restores neuronal viability impaired by Aβ42 oligomers (Aβo) (Aβo (+)) (Fig 2D–F). In the presence of Aβo, cultured neurons had significantly reduced viability (*P* < 0.0001) and intracellular ATP levels (*P* < 0.001), showing the neurotoxic impact of Aβo on neuron survival (p3‐Alcβ(−)/Aβo(+) versus p3‐Alcβ(−)/Aβo(−) in Fig 2D and E). Treating neurons with Aβo significantly increased LDH levels (*P* < 0.0001) (Fig 2F), demonstrating the deleterious impact of Aβo on membrane integrity. Treatment with p3‐Alcβ37 and p3‐Alcβ9‐19 preserved neuronal viability (^#^ *P* < 0.01, ^#^ *P* < 0.01) (Fig 2D) and ATP generation (^#^ *P* < 0.05, ^#^ *P* < 0.01) (Fig 2E) in the presence of Aβo and suppressed Aβo‐induced LDH release (^#^ *P* < 0.05, ^#^ *P* < 0.05) (Fig 2F). A high concentration of Aβo (10 μM) induces stronger neurotoxicity than the low one (2.5 μM). The same concentration (10 μM) of p3‐Alcβ peptides partially restored neuronal viability that had been beforehand damaged by Aβo (10 μM) (Fig EV1G). These data indicate that p3‐Alcβ37 and p3‐Alcβ9‐19 protect neurons against Aβo‐mediated neurotoxic damage which might be not so severe as it causes cell death. In AD, Aβo triggers the generation of reactive oxygen species (ROS), which causes oxidative stress in neurons (Cheignon *et al*, 2018). Incubating mouse primary cultured neurons with Aβo increased ROS production (*P* < 0.05). However, this increase was completely abolished in the presence of p3‐Alcβ peptides (Fig 2G). These data suggest that p3‐Alcβ can preserve mitochondrial function in neurons compromised by Aβo.

### p3‐Alcβ inhibits anomalous Aβ42 oligomer‐induced Ca^2+^ influx in neurons

**Figure 3:**
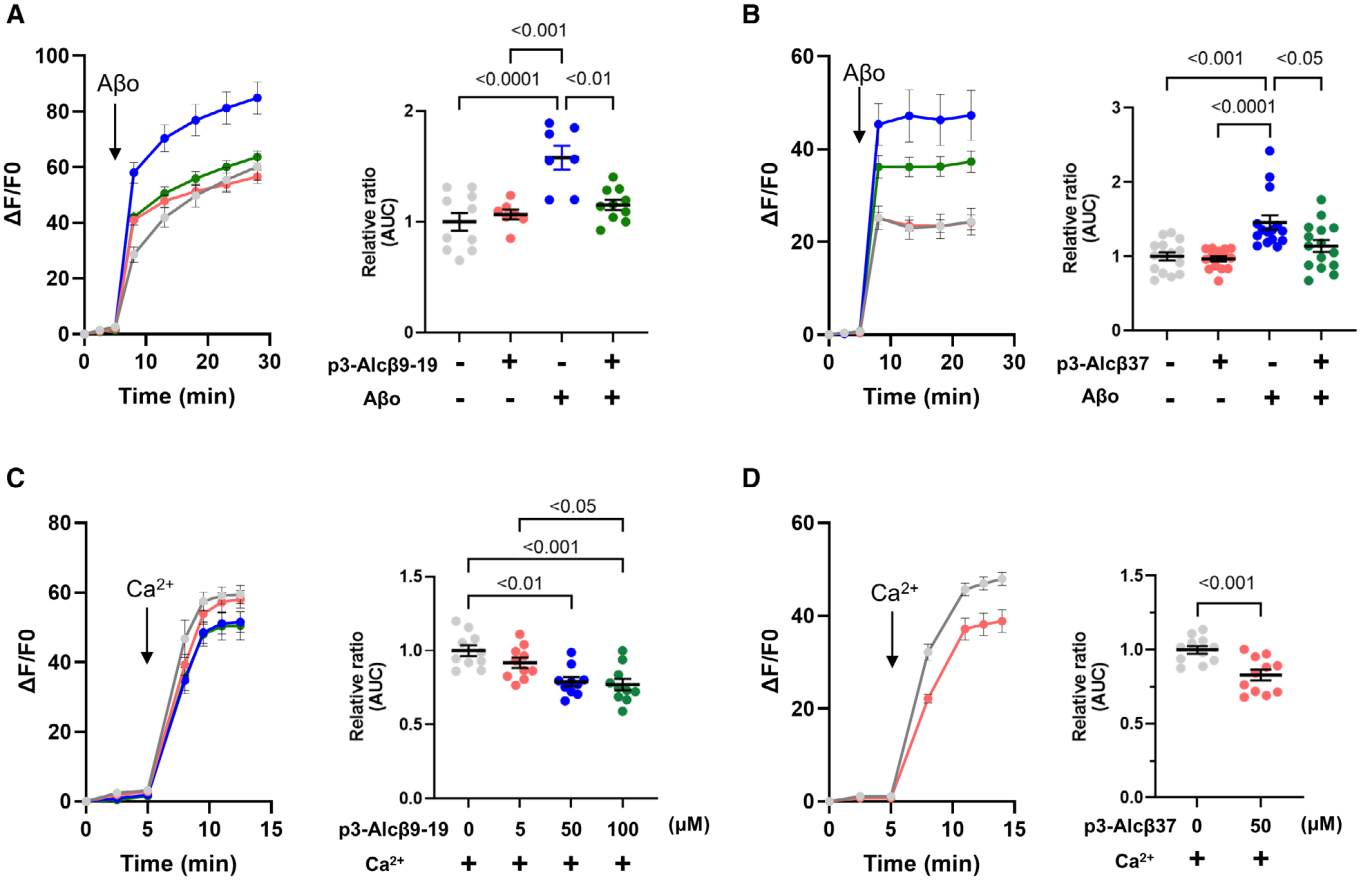
Suppression of Aβ42‐triggered neuronal Ca^2+^ influx by p3‐Alcβ A, BSuppression of Ca^2+^ influx induced by Aβo in neurons treated with p3‐Alcβ9‐19 (A) and p3‐Alcβ37 (B). Mouse neurons (div 11–13) pretreated with Fluo 4‐AM were stimulated at 5 min (arrow) with (+) or without (−) Aβo (5.2 μM) in the presence (+) or absence (−) of p3‐Alcβ (50 μM). The fluorescence intensity was recorded at the indicated time (left) and the fluorescence area intensity for 18 min (5–23 min at time points) is shown (right) as the AUC expressed relative to that of cells cultured in the absence (−) of p3‐Alcβ and Aβo (assigned a value of 1.0).C, DSuppression of Ca^2+^ influx into neurons followed by Ca^2+^ administration in the presence of p3‐Alcβ9‐19 (C) and p3‐Alcβ37 (D). Mouse neurons (div 11–13) pretreated with Fluo 4‐AM in the calcium‐depleted medium were administered Ca^2+^ (2 mM) at 5 min (arrow) in the presence or absence of p3‐Alcβ9‐19 (C) and p3‐Alcβ37 (D). The fluorescence intensity was recorded at the indicated time (left) and the fluorescence area intensity for 7.5 or 9 min (5 to 12.5 min in panel (C) and 5 to 14 min in panel (D) at the indicated time points) is shown (right). Statistical significance was determined by one‐way ANOVA with the Tukey's multiple comparison test (mean ± SE; *n* = 14 (A), *n* = 15 (B), *n* = 10 (C)). The significant *P*‐values (*P* < 0.05, *P* < 0.01, *P* < 0.001, *P* < 0.0001) are indicated on the graphs. Statistical significance was determined with a Student's *t*‐test (mean ± SEM; *n* = 11 (D)), and the significant *P*‐value (*P* < 0.001) is indicated on the graph. Data information: Experimental numbers indicate biological replicates. Detailed information including the statistical summary is described in Dataset EV1. Source data are available online for this figure.

**Figure EV2:**
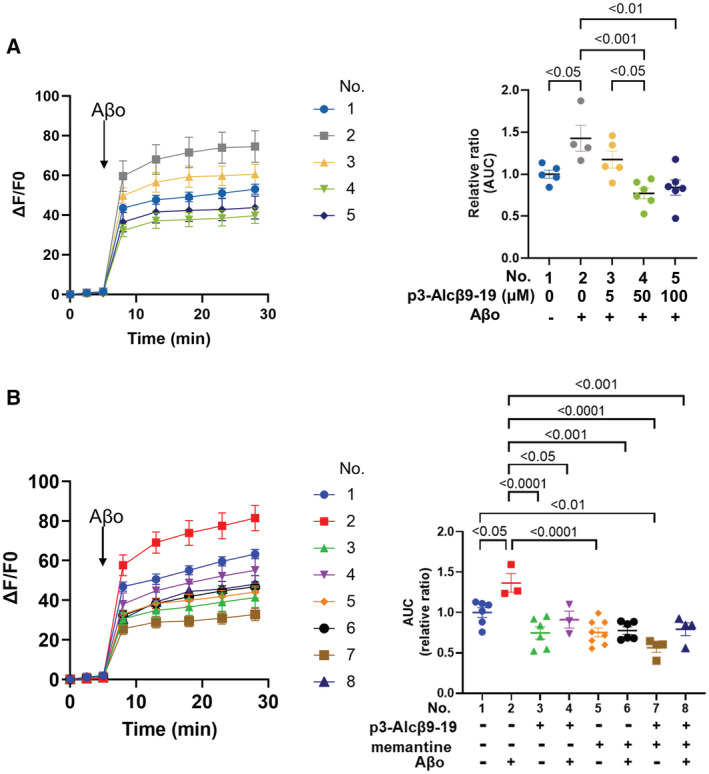
p3‐Alcβ9‐19 dose‐dependently suppresses Aβ42‐triggered neuronal Ca^2+^ influx and nonsynergistic suppression of Ca^2+^ influx induced by Aβ42 oligomers (Aβo) in neurons by p3‐Alcβ and memantine Dose–response effect of p3‐Alcβ9‐19. Mouse neurons (div 15) pretreated with Fluo 4‐AM were stimulated at 5 min (arrow) with (+) or without (−) Aβo (5.2 μM) in the presence of the stipulated amount of p3‐Alcβ9‐19 (μM). The fluorescence intensity was recorded at the indicated time (left) and the fluorescence area intensity for 23 min (5–28 min at the indicated time points) is shown (right) as the AUC expressed relative to that of cells cultured in the absence of p3‐Alcβ and Aβo (assigned a value of 1.0 in No. 1). Statistical significance was determined with a one‐way ANOVA with the Tukey's multiple comparison test (mean ± SEM; *n* = 4–6) and significant *P*‐values (*P* < 0.05, *P* < 0.01, *P* < 0.001) are indicated on the graph.Effects of p3‐Alcβ and memantine. Neurons (div 13) pretreated with Fluo 4‐AM were stimulated with or without Aβo (5.2 μM) at 5 min (arrow) in the presence (+) or absence (−) of p3‐Alcβ9‐19 (50 μM) and memantine (5 μM). Fluorescence intensity was recorded at the indicated time (left), and the fluorescence area intensity for 23 min (5–28 min at the indicated time points) is shown (right) as the AUC expressed relative to that of cells cultured in experiment No. 1 (assigned a value of 1.0). Statistical significance was determined by a one‐way ANOVA with the Tukey's multiple comparison test (mean ± SEM; *n* = 3–8), and significant *P*‐values (*P* < 0.05, *P* < 0.01, *P* < 0.001, *P* < 0.0001) are indicated on the graph. Data information: Experimental numbers indicate biological replicates. Detailed information including the statistical summary is described in Dataset EV2. Source data are available online for this figure.

Aβo promotes neuronal dysfunction by triggering excessive Ca^2+^ entry into neurons (Hardingham & Bading, 2010; Benilova *et al*, 2012). To determine how p3‐Alcβ restores neuronal function and protects neurons against Aβo‐induced toxicity, we examined Ca^2+^ influx, with a labeled calcium indicator Fluo 4‐AM, into mouse primary neurons in culture. Aβo increased Ca^2+^ influx dramatically, and this increase was significantly inhibited in the presence of p3‐Alcβ9‐19 (*P* < 0.01) or p3‐Alcβ37 (*P* < 0.05) (Figs 3A and B, and EV2A). To uncover the role of p3‐Alcβ on Ca^2+^ influx into neurons, mouse primary neurons were first incubated in a Ca^2+^‐depleted medium to reduce their excitability. CaCl_2_ (2 mM), at a concentration equivalent to that found in CSF (Forsberg *et al*, 2019), was then added to the culture medium, and intracellular Ca^2+^ levels were recorded in the presence or absence of p3‐Alcβ9‐19 or p3‐Alcβ37 (Fig 3C and D). Intracellular Ca^2+^ levels rapidly increased after Ca^2+^ administration, but this response was dose‐dependently suppressed by the presence of p3‐Alcβ9‐19. This finding was also observed in the presence of p3‐Alcβ37, indicating that p3‐Alcβ attenuates Ca^2+^ influx.

**Figure EV3:**
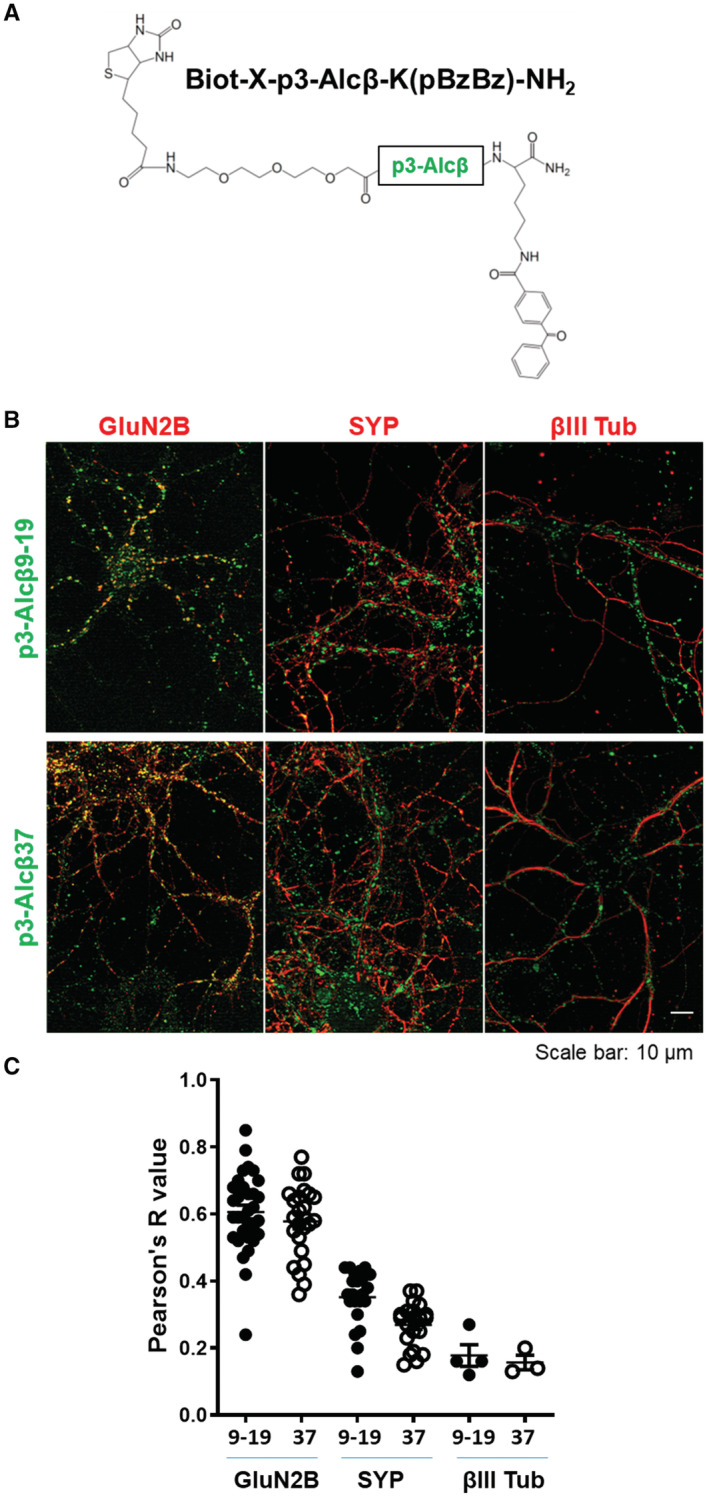
Colocalization of p3‐Alcβ associated to neurons with neuronal proteins Structure of biotin‐X‐p3‐Alcβ‐K(pBzBz)‐NH_2_. Alcβ‐KO mouse neurons (div 14) were incubated with biotin‐X‐p3‐Alcβ9‐19‐K(pBzBz)‐NH_2_ or biotin‐X‐p3‐Alcβ37‐K(pBzBz)‐NH_2_ followed by UV irradiation.Localization of biotin‐X‐p3‐Alcβ‐K(pBzBz)‐NH_2_ probes associated with neurons. The cells were fixed and immunostained with antibodies against GluN2B, synaptophysin (SYP), and βIII‐tubulin (βIII Tub). Localization of p3‐Alcβ was visualized with streptavidin‐Alexa488 (green), while the immunoreactive neuronal proteins were localized with an Alexa546‐conjugated second antibody (red). Scale bar, 10 μm.Colocalization efficiency between p3‐Alcβ and neuronal proteins. Colocalization was calculated using the coloc2 plug‐in. The colocalization rates of proteins and p3‐Alcβ were calculated from each frame of images (15,750 μm^2^) of neurons and are indicated as Pearson's *R* value. Independent cell stainings were performed one to three times per cell preparation, and three to five frames were acquired from each well. All values were combined and subjected to statistical analysis with the indicated number of independent biological repeats (mean ± SEM). Pearson's coefficients are shown (*R* value of 1.0 indicates perfect colocalization while an *R* value of 0 indicates random localization). Data information: Experimental numbers indicate frame numbers. Detailed information including the statistical summary is described in Dataset EV2. Source data are available online for this figure.

To examine the regional localization of p3‐Alcβ, we used immunocytochemistry to investigate the colocalization of p3‐Alcβ with neuronal proteins (Fig EV3). Here, we used the photo‐affinity probes biotin‐X‐p3‐Alcβ9‐19‐K(pBzBz)‐NH_2_ and biotin‐X‐p3‐Alcβ1‐37‐K(pBzBz)‐NH_2_ (Fig EV3A). Primary cultured neurons derived from Alcβ‐KO mice (Gotoh *et al*, 2020), which do not produce endogenous p3‐Alcβ, were incubated with the appropriate probes for 1 h, followed by ultraviolet irradiation and fixation. The neurons were incubated with the designated antibodies (*red*) and streptavidin‐Alexa488 (*green*) to detect p3‐Alcβ. Pearson's *R*‐values revealed that p3‐Alcβ colocalized with neuronal proteins (Fig EV3B and C). The postsynaptic glutamate receptor subunit GluN2B colocalized better with p3‐Alcβ than with presynaptic synaptophysin (SYP) or cytoplasmic βIII‐tubulin. These results suggest that p3‐Alcβ acts on a membrane protein(s) in the postsynaptic region in neurons.

### p3‐Alcβ suppresses the function of NMDARs, which are anomalously activated for Ca^2+^ influx by Aβ42 oligomers

**Figure 4:**
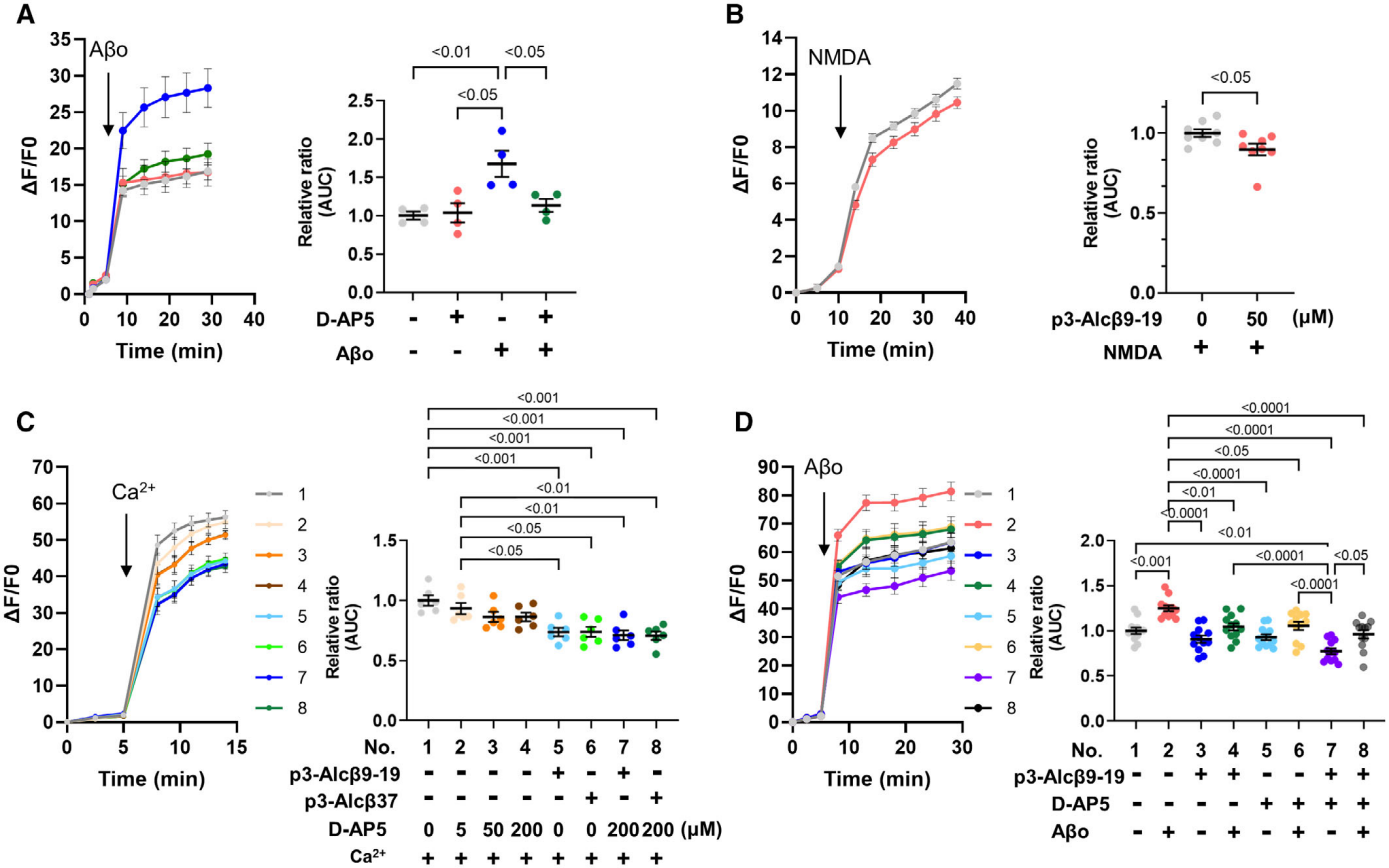
Suppression of Ca^2+^ influx by p3‐Alcβ is distinct from that by an NMDA receptor antagonist Suppression of Ca^2+^ influx induced by Aβ42 oligomers (Aβo) in neurons treated with the NMDA receptor antagonist D‐AP5. Neurons (div 14) pretreated with Fluo 4‐AM were stimulated with or without Aβo (5.2 μM) at 5 min (arrow) in the presence or absence of D‐AP5 (50 μM). The fluorescence intensity was recorded at the indicated time (left) and the fluorescence area intensity for 24 min (5–29 min at the indicated time points) is shown (right) as the AUC expressed relative to that of cells cultured in the absence of D‐AP5 and Aβo (assigned a value of 1.0). Statistical significance was determined by one‐way ANOVA with the Tukey's multiple comparisons test (mean ± SE; *n* = 4), and significant *P*‐values (*P* < 0.05, *P* < 0.01) are indicated on the graph.Suppression of Ca^2+^ influx induced by excessive NMDA in neurons treated with p3‐Alcβ9‐19. Mouse neurons (div 11–13) pretreated with Fluo 4‐AM were stimulated with NMDA (52 μM) at 10 min (arrow) in the presence or absence of p3‐Alcβ9‐19 (50 μM). The fluorescence intensity was recorded at the indicated time (left) and the fluorescence area intensity for 28 min (10–38 min at the indicated time points) is shown (right) as the AUC expressed relative to that of cells cultured in the absence of p3‐Alcβ9‐19 (assigned a value of 1.0). Statistical significance was determined by the Student's *t*‐test (mean ± SEM; *n* = 8), and the significant *P*‐value (*P* < 0.05) is indicated on the graph.Suppression of Ca^2+^ influx into neurons followed by Ca^2+^ administration in the presence or absence of D‐AP5 and p3‐Alcβ. Neurons (div 11–14) pretreated with Fluo 4‐AM in Ca^2+^‐depleted medium were administered Ca^2+^ (final 2 mM) at 5 min (arrow) in the presence (5, 50, 200 μM) or absence (0 μM) of D‐AP5, p3‐Alcβ9‐19 (+, 50 μM) and p3‐Alcβ37 (+, 50 μM). The fluorescence intensity was recorded at the indicated time (left) and the fluorescence area intensity for 9 min (5–14 min at time points) is shown (right) as the AUC expressed relative to that of cells cultured in experiment No. 1 (assigned a value of 1.0). Statistical significance was determined with a one‐way ANOVA with the Tukey's multiple comparisons test (mean ± SEM; *n* = 6), and significant *P*‐values (*P* < 0.05, *P* < 0.01, *P* < 0.001) are indicated on the graph.Nonsynergistic suppression of Ca^2+^ influx induced by Aβ42 oligomers (Aβo) in neurons by p3‐Alcβ and D‐AP5. Neurons (div 14) pretreated with Fluo 4‐AM were stimulated with or without Aβo (5.2 μM) at 5 min (arrow) in the presence (+) or absence (−) of p3‐Alcβ9‐19 (50 μM) and D‐AP5 (50 μM). The fluorescence intensity was recorded at the indicated time (left) and the fluorescence area intensity for 23 min (5–28 min at the indicated time points) is shown (right) as the AUC expressed relative to that of cells cultured in experiment No. 1 (assigned a value of 1.0). Statistical significance was determined with a one‐way ANOVA with the Tukey's multiple comparison test (mean ± SEM; *n* = 12), and significant *P*‐values (*P* < 0.05, *P* < 0.01, *P* < 0.001, *P* < 0.0001) are indicated on the graph. Data information: Experimental numbers indicate biological replicates. Detailed information including the statistical summary is described in Dataset EV1. Source data are available online for this figure.

Aβo preferentially targets neuronal glutamate receptors. The stimulation of postsynaptic N‐methyl‐D‐aspartate receptors (NMDARs) by Aβo induces severe neuronal degeneration via intracellular Ca^2+^ overload (Zhang *et al*, 2007; Hardingham & Bading, 2010), which is more detrimental to neurons than Ca^2+^ influx mediated by other channels (Tymianski *et al*, 1993). In mouse primary cultured neurons, Aβo‐induced Ca^2+^ entry increased and was suppressed in the presence of the NMDA antagonist D‐AP5 (Fig 4A). This finding reiterates previous data that Aβo‐mediated neurotoxicity is largely mediated by NMDARs (Zhang *et al*, 2007; Hardingham & Bading, 2010). NMDARs are the major Ca^2+^ channels in the postsynaptic region in neurons. These receptors are usually inactivated by Mg^2+^ binding, and membrane depolarization via Na^+^ influx is required to activate NMDARs. Moreover, Aβos are thought to activate NMDARs in an unusual manner (Hardingham & Bading, 2010; Benilova *et al*, 2012). In fact, NMDA administration resulted in a very smaller Ca^2+^ influx (See the difference between the magnitude of the vertical axis in panel B and that in panel A) into neurons in the absence of p3‐Alcβ9‐19 than into neurons in the presence of p3‐Alcβ9‐19 (Fig 4B). This indicated that most NMDARs are largely inactive in mouse primary neurons and that p3‐Alcβ regulates Ca^2+^ influx weakly even in the presence of NMDA. This suggests that p3‐Alcβ does not directly act on NMDARs, which remain mostly silent in the absence of Aβo but regulates Ca^2+^ influx by targeting other molecules in a milder manner than when NMDARs are activated by Aβo.

To determine whether p3‐Alcβ targets protein(s) other than NMDARs to control Ca^2+^influx, mouse primary cultured neurons were first incubated in a Ca^2+^‐depleted medium to reduce their excitability and were then administered CaCl_2_ (2 mM) in the presence or absence of D‐AP5 and p3‐Alcβ (Fig 4C). Ca^2+^ influx was slightly, but not significantly, reduced by D‐AP5 (5, 50, and 200 μM), indicating that NMDARs were not main contributors to Ca^2+^ influx through extracellular Ca^2+^ administration in mouse primary neurons (compare experiment numbers/No. 2 to No. 4 with No. 1 in Fig 4C). Interestingly, this Ca^2+^ influx was significantly inhibited by p3‐Alcβ9‐19 and p3‐Alcβ37 (*P* < 0.001) (compare No. 5 and No. 6 with No. 1). Furthermore, when D‐AP5 (200 μM) was combined with p3‐Alcβ9‐19 or p3‐Alcβ37, the intracellular Ca^2+^ levels were the same as that of p3‐Alcβ alone (compare No. 7 and No. 8 with No. 5 and No. 6). These results clearly show that p3‐Alcβ does not target NMDARs directly but regulates Ca^2+^ influx through a different molecular mechanism.

It is intriguing if p3‐Alcβ is capable of regulating a significant amount of Ca^2+^ influx under the Aβo‐induced NMDARs activation condition. To this aim, we further investigated whether the unique Aβo‐mediated Ca^2+^ influx into neurons was synergistically suppressed by p3‐Alcβ and D‐AP5 (Fig 4D). Mouse primary neurons were stimulated with (+) or without (−) Aβo in the presence or absence of p3‐Alcβ9‐19 and D‐AP5. Aβo‐triggered Ca^2+^ influx was suppressed significantly with either p3‐Alcβ9‐19 (*P* < 0.01) or D‐AP5 (*P* < 0.05) (compare No. 4 or No. 6 with No. 2). The intracellular Ca^2+^ load in the presence of both p3‐Alcβ9‐19 and D‐AP5 was the same level as that in the presence of p3‐Alcβ9‐19 or D‐AP5 alone (compare No. 4 or No. 6 with No. 8), and no synergistic effect was observed. This finding strongly suggests that p3‐Alcβ inhibits Aβo‐induced NMDAR‐mediated excessive Ca^2+^ influx indirectly via other protein(s). Similar results were obtained with memantine, an NMDAR antagonist that is used to treat patients with moderate‐to‐severe AD (Fig EV2B).

### Peripheral administration of p3‐Alcβ restores neuronal viability impaired by Aβ accumulation in the brain of the AD mouse model

**Figure 5:**
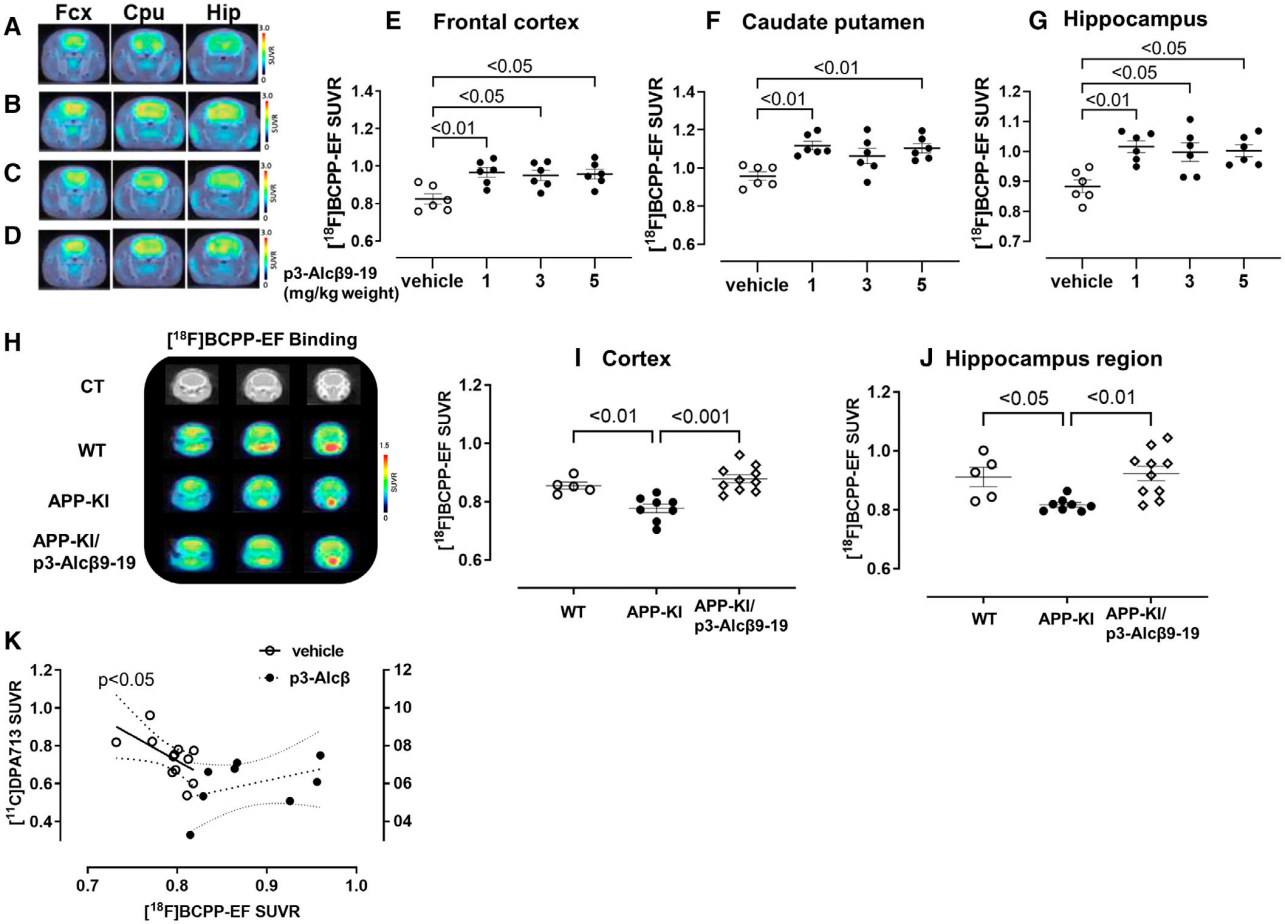
Coronal parametric PET images of [^18^F]BCPP‐EF SUVR in rats and AD mouse model following the subcutaneous injection of p3‐Alcβ9‐19 A–DThe SUVRs of [^18^F]BCPP‐EF in the frontal cortex (Fcx), caudate putamen (Cpu), and hippocampus (Hip) of rats with (1 mg (B), 3 mg (C), and 5 mg (D) per kg body weight) or without (vehicle (A)) subcutaneous administration of p3‐Alcβ9‐19. The PET data are superimposed on X‐ray CT images, and the color bar denotes the SUVR.E–GThe SUVRs of groups administered p3‐Alcβ9‐19 versus the vehicle‐treated group. Statistical analysis was performed using one‐way ANOVA, followed by Bonferroni correction for multiple comparisons (mean ± SEM; *n* = 6), and significant *P*‐values (*P* < 0.05, *P* < 0.01) are indicated on the graphs.HThe SUVRs of [^18^F]BCPP‐EF in the cortex and hippocampus of wild‐type (WT) and AD mice (*App* ^*NL‐F/NL‐F*^) with (APP‐KI/p3‐Alcβ9‐19) or without (APP‐KI) a subcutaneous administration of p3‐Alcβ9‐19 (1 mg/kg body weight). The PET data are superimposed on X‐ray CT images, and the color bar denotes the SUVR.I, JThe SUVRs of [^18^F]BCPP‐EF in the cortex (I) and hippocampus (J) are compared across the three groups. Statistical analysis was performed using one‐way ANOVA, followed by Bonferroni correction for multiple comparisons (mean ± SEM; *n* = 6–10), and significant *P*‐values (*P* < 0.05, *P* < 0.01, *P* < 0.001) are indicated on the graphs.KImprovement of the inverse SUVR correlation between [^18^F]BCPP‐EF and [^11^C]DPA713 in AD mice following administration of p3‐Alcβ9‐19. AD mice with (closed circle) or without (open circle) subcutaneous injection of p3‐Alcβ9‐19 peptide (1 mg/kg body weight) are also analyzed with [^11^C]DPA713. A solid linear line shows a significant inverse correlation (Pearson's correlation, *r* = 0.594, *P* < 0.05) in AD mice, whereas a dotted linear line shows a positive correlation tendency in AD mice in response to p3‐Alcβ9‐19 administration. Data information: Experimental numbers indicate biological replicates. Detailed information including the statistical summary is described in Dataset EV1. Source data are available online for this figure.

**Figure EV4:**
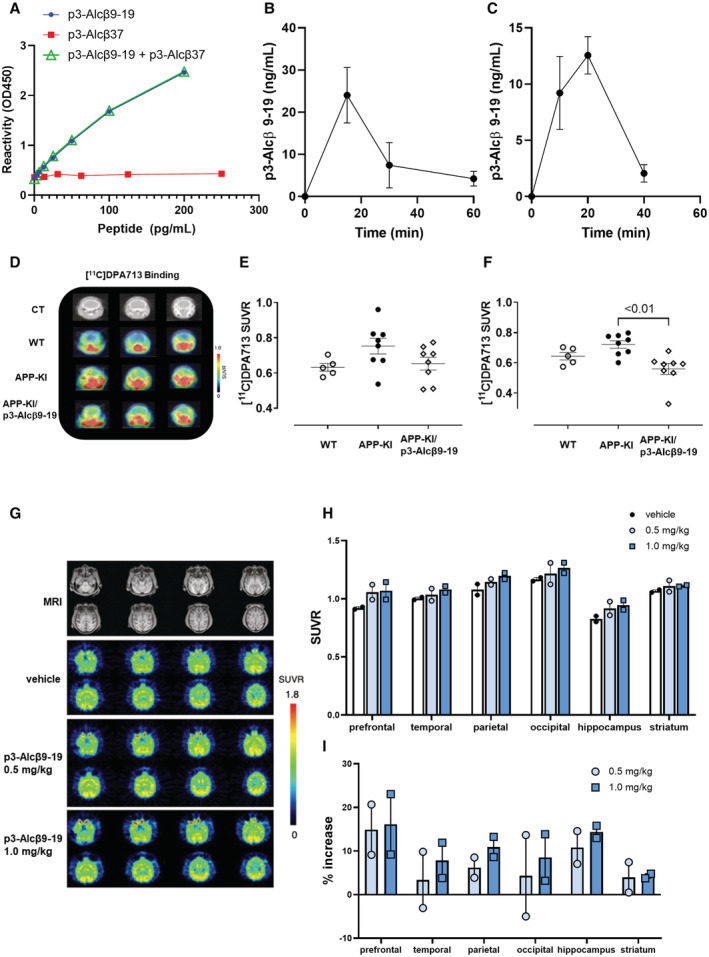
Specific sELISA system for the detection of p3‐Alcβ9‐19 and transport of subcutaneously administered p3‐Alcβ into blood and CSF, and coronal parametric PET images of [^11^C]DPA713 SUVR in wild‐type and AD mouse model following subcutaneous injection of p3‐Alcβ9‐19, and PET images of [^18^F]BCPP‐EF SUVR in monkeys following transdermal administration of vehicle and p3‐Alcβ9‐19 A–CSandwich ELISA for the quantification of p3‐Alcβ9‐19 and the determination of the pharmacokinetic profile of p3‐Alcβ9‐19. (A) Specific reactivity of the sELISA system with p3‐Alcβ9‐19. Indicated amounts of synthetic p3‐Alcβ9‐19 (closed circles), p3‐Alcβ37 (closed squares), and the mixtures of the indicated amounts of p3‐Alcβ9‐19 with 1,000 pg/ml p3‐Alcβ37 (open triangles) were dissolved in buffer A (PBS containing 1% bovine serum albumin and 0.05% Tween‐20) and assayed in duplicate by sELISA. Immunoreactivities were detected as described in “Materials and Methods.” Results are reported as mean ± SEM (*n* = 2). (B) Transport of p3‐Alcβ9‐19 into the blood. p3‐Alcβ9‐19 (1 mg/kg of body weight, *n* = 4 per time point) was administered subcutaneously to wild‐type (WT) mice (9‐month‐old). Blood was collected from their tail veins at the indicated time points, the plasma was diluted 200‐fold, and p3‐Alcβ9‐19 concentrations were quantified by sELISA. Results are reported as mean ± SEM (C) Transport of p3‐Alcβ9‐19 into CSF. P3‐Alcβ9‐19 (5 mg/kg body weight, *n* = 4 per time point) was administered subcutaneously to WT mice (5‐month‐old). CSF was collected from four mice, respectively, at the indicated time points and diluted 20‐fold, and p3‐Alcβ9‐19 concentrations were quantified by sELISA. Results are reported as mean ± SEM.D–FPET imaging with [^11^C]DPA713. (D) The SUVRs of [^11^C]DPA713 in the cortex and hippocampus of wild‐type (WT) and AD mice (APP‐KI) with (APP‐KI/p3‐Alcβ9‐19) or without (APP‐KI) subcutaneous injection of p3‐Alcβ9‐19 (1 mg/kg body weight). The PET data are superimposed on X‐ray CT images, and the color bar denotes the SUVR. (E, F) The SUVRs of [^18^F]BCPP‐EF in the cortex (E) and hippocampus (F) are compared. Statistical analysis was performed using a one‐way ANOVA, followed by Bonferroni correction for multiple comparisons (mean ± SEM; *n* = 5–7), and the significant *P*‐value (*P* < 0.01) is indicated on the graph.G–IIncreased mitochondrial activity after administration of p3‐Alcβ9‐19 to monkeys. Three consecutive PET scans in the same *Rhesus* monkeys (*n* = 2) were performed after the transdermal administration of vehicle and p3‐Alcβ9‐19 (0.5 mg/kg and 1 mg/kg). The PET images of [^18^F]BCPP‐EF SUVR were displayed in parallel with magnetic resonance (MR) images (G), and the color bar denotes the SUVR (H). The bar graph indicates the percentile increase in binding (I). Results are reported as mean ± SEM (*n* = 2). Data information: Experimental numbers indicate biological replicates with two monkeys. Detailed information including the statistical summary is described in Dataset EV2. Source data are available online for this figure.

Next, we examined the ability of p3‐Alcβ9‐19 to promote and preserve neuronal health against Aβ toxicity *in vivo*. Rodents were peripherally administered p3‐Alcβ9‐19 and neuronal viabilities were then analyzed with positron emission tomography (PET) imaging. In a separate study in mice, successful transfer of p3‐Alcβ9‐19 into the brain following subcutaneous injection was confirmed by sELISA by quantifying p3‐Alcβ9‐19 levels in the CSF (Fig EV4A–C). An increase in neuronal viability caused by p3‐Alcβ was confirmed *in vivo* by monitoring brain mitochondrial function using PET imaging with a [^18^F]BCPP‐EF probe. This probe detects mitochondrial complex I activity, which reflects neuronal viability in the living brain (Tsukada *et al*, 2014). Rats (8‐week‐old) that received p3‐Alcβ9‐19 (0, 1, 3, 5 mg/kg body weight) subcutaneously were scanned for 90 min after intravenous injection of [^18^F]BCPP‐EF through a tail vein. Parametric PET images of [^18^F]BCPP‐EF standard uptake value ratios (SUVRs) were superimposed on the CT images of all rats (Fig 5A–D). As described elsewhere (Hosoya *et al*, 2017), elliptical regions of interest (ROIs) ranging from 12 to 24 mm^2^ were placed over the frontal cortex (Fcx), caudate putamen (Cpu), and hippocampus (Hip) by referring to the X‐ray CT images (Yamagishi *et al*, 2019). SUVR levels were compared among brain regions (Fig 5E–G). The SUVR in p3‐Alcβ9‐19‐treated rats was significantly higher in all brain regions than in vehicle‐treated rats, with the exception of the caudate putamen in animals that received 3 mg/kg p3‐Alcβ9‐19. This finding clearly shows that peripherally administered p3‐Alcβ9‐19, at a dose of 1 mg/kg body weight, increases neuronal survival while also activating mitochondrial function in the brain.

Mitochondrial dysfunction is common in the brain of AD patients (Ridge & Kauwe, 2018), and decreased viability of vulnerable brain regions can be detected by PET imaging with [^18^F]BCPP‐EF (Tsukada *et al*, 2014). Therefore, we next investigated the reduction in [^18^F]BCPP‐EF SUVR in the brain of AD mice (*App* ^*NL‐F/NL‐F*^). These animals generate predominantly human Aβ42 and exhibit amyloid accumulation, a hallmark of AD pathology (Saito *et al*, 2014). We tested whether p3‐Alcβ9‐19 could restore neuronal activity (Fig 5H–J). AD mice (APP‐KI) showed a significant decrease in [^18^F]BCPP‐EF SUVR in the cortex and hippocampus compared with age‐matched (12‐month‐old) wild‐type (WT) mice. SUVR values in AD mice were restored to levels similar to those in WT mice after a single subcutaneous injection of p3‐Alcβ9‐19 (1 mg/kg) (APP‐KI/p3‐Alcβ9‐19 in Fig 5H–J). This indicates that peripheral administration of p3‐Alcβ restores the viability of mouse neurons impaired by the accumulation of human Aβ42.

The level of neuroinflammation was assessed in AD mice (APP‐KI), in parallel with PET imaging using [^11^C]DPA713, a translocator protein (TSPO) PET radiotracer (Hosoya *et al*, 2017). The parametric PET images of [^11^C]DPA713 SUVR were superimposed on CT images (Fig EV4D–F). In these AD mice, SUVRs of [^11^C]DPA713 showed a significant inverse correlation with those of [^18^F]BCPP‐EF (vehicle) (*P* < 0.05), demonstrating that the decreased survival and mitochondrial impairment in neurons are associated with an increase in neuroinflammation, which may be evoked by Aβ burden (vehicle in Fig 5K). This inverse correlation tended to change to a positive correlation in p3‐Alcβ9‐19 (p3‐Alcβ, Fig 5K)‐injected AD mice, suggesting that the restoration of neuronal viability through p3‐Alcβ9‐19 treatment may be associated with its neuroprotective property.

We further investigated the effect of p3‐Alcβ9‐19 on mitochondrial activity in monkeys (Fig EV4G–I). Three consecutive PET scans in *Rhesus* monkeys (*n* = 2) were performed after the transdermal administration of vehicle and p3‐Alcβ9‐19 (0.5 and 1 mg/kg) with PassPort System (Ono *et al*, 2022). The PET images of [^18^F]BCPP‐EF SUVR, which reflect the degree of mitochondrial activity, show a marked increase in [^18^F]BCPP‐EF binding in monkeys that were treated with 1 mg/kg of p3‐Alcβ9‐19 (Fig EV4H, dark blue bar). The dose (1 mg/kg) was also used in the current *in vivo* study in rodents. As shown in the bar graph (Fig EV4I), the percentile increase in binding is observed in the cerebral cortex and hippocampus. The results suggest that experiments in monkeys with transdermal administration of the p3‐Alcβ9‐19 pharmaceutical formulation are sufficient for delivering the peptide into the brain. Although the number of monkeys used in this study was small, the administration of p3‐Alcβ9‐19 (especially in 1.0 mg/kg body weight) indeed raised mitochondrial activity in the brain of monkeys.

### p3‐Alcβ level in the central nervous system decreases with age in monkeys

**Figure EV5:**
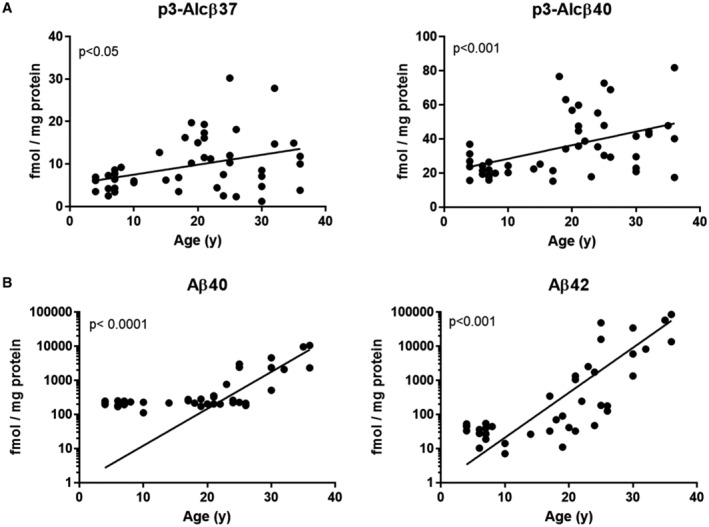
Age‐dependent changes of p3‐Alcβ and Aβ in monkey brain parenchyma A, BTemporal cortex tissue from *Cynomolgus* monkeys of various ages (y, years old, *n* = 47) was used. Changes in p3‐Alcβ37 and p3‐Alcβ40 levels (A), and Aβ40 and Aβ42 levels (B) in the TBS‐insoluble fraction are shown as a function of age. Statistical analysis was performed using Pearson's correction (A left, *r* ^2^ = 0.1379; A right *r* ^2^ = 0.216; B left, *r* ^2^ = 0.3347; B right, *r* ^2^ = 0.2873); *P*‐values < 0.05 were considered statistically significant. Data information: Experimental numbers indicate biological replicates. Detailed information including the statistical summary is described in Dataset EV2. Source data are available online for this figure.

We previously reported that endogenous p3‐Alcβ levels in CSF of monkeys decrease with age (Hata *et al*, 2019). We further analyzed age‐related changes in brain p3‐Alcβ and Aβ levels using the monkey brain (Fig EV5). Similar to humans, the monkey brain parenchyma showed increased Aβ accumulation with age. For Aβ42, the brain Aβ level in monkeys was 10^1^ to 10^2^ fmol/mg protein under 20 years old and 10^3^ to 10^4^ fmol/mg protein over 30 years old as described previously (Nishimura *et al*, 2012). By contrast, p3‐Alcβ levels remain very low (below 10^2^ fmol/mg protein) throughout the aging process, eventually falling below a centesimal to that of Aβ in aged individuals. Endogenous p3‐Alcβ levels may be too low to protect neurons against neurotoxic Aβo that accumulates in the brain of aged individuals.

## Discussion

**Figure 6:**
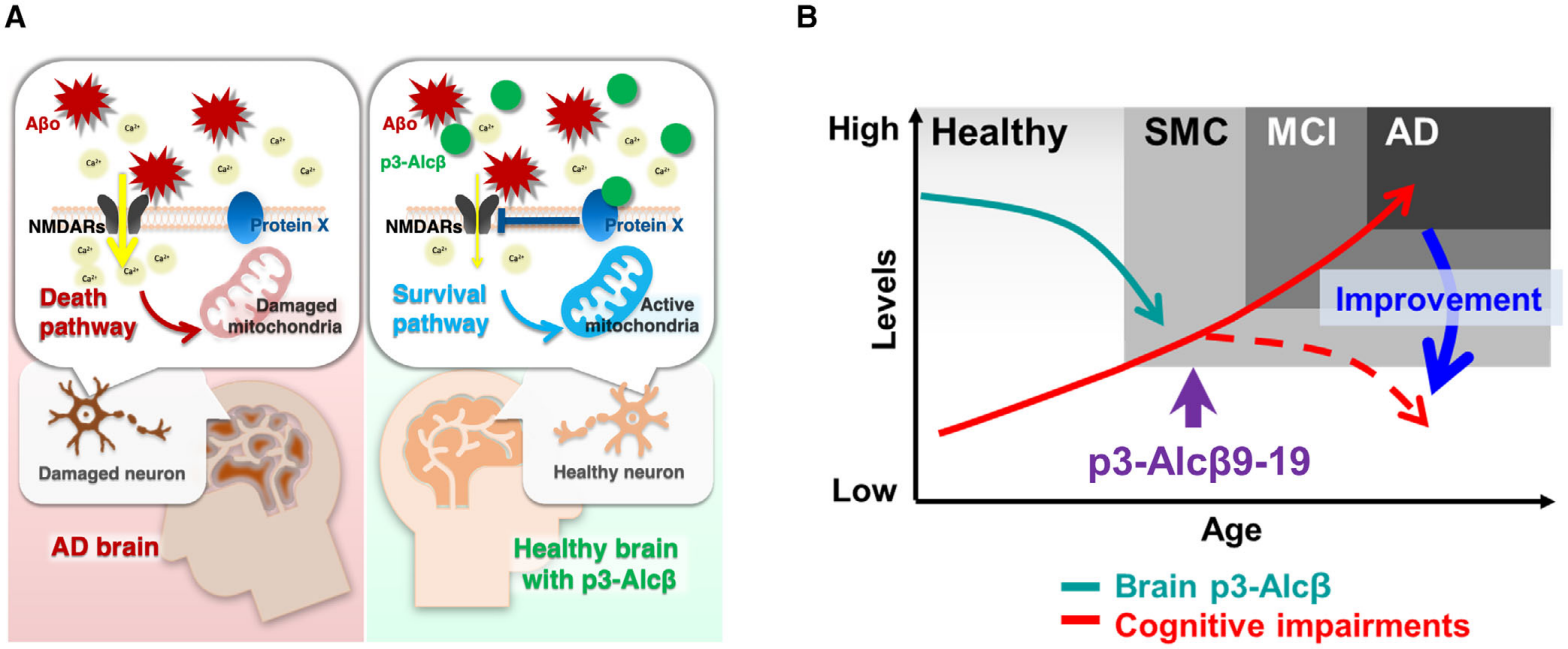
Possible mechanism of p3‐Alcβ for suppressing Aβ oligomer‐induced neurotoxicity and therapeutic strategy Schematic mechanism of p3‐Alcβ to suppress the neurotoxicity by Aβo. One of the major targets of Aβ oligomers (Aβo) is NMDA receptors (NMDARs). Aβo triggers unregulated intracellular Ca^2+^ influx into neurons, activating the cell death pathway (left). p3‐Alcβ regulates an unidentified membrane protein X to attenuate intracellular Ca^2+^ influx, which inhibits NMDARs that are unusually activated by Aβo. This may activate the survival pathway and increases mitochondrial activity in neurons (right). Since p3‐Alcβ is an endogenous brain peptide, it may help to maintain a healthy brain by reversibly regulating NMDAR‐ and Aβo‐mediated Ca^2+^ influx.Therapeutic strategy with p3‐Alcβ9‐19 pharmaceutical formulation. Brain p3‐Alcβ level decreases and cognitive impairments increase with age. Administration of p3‐Alcβ9‐19 in the early stage of dementia is expected to restore brain function. SMC, subjective memory complaints; MCI, mild cognitive impairment; AD, Alzheimer's disease.

In this study, we found the neuroprotective function of p3‐Alcβ, which is generated from Alcβ by a metabolism similar to APP (Araki *et al*, 2004; Hata *et al*, 2009), and propose a neuroprotective mechanism mediated by p3‐Alcβ (Fig 6A). Endogenously‐derived neuronal p3‐Acβ suppresses Ca^2+^ influx enhanced by Aβo‐induced NMDAR activation, causing to restore neurons to the healthy state presumably through suppressing the cell death pathway. The detailed molecular mechanisms as to how p3‐Alcβ regulates Aβo‐triggered toxic Ca^2+^ influx in neurons and upregulates mitochondrial function remain elusive. However, our results suggested that peripheral administration of p3‐Alcβ9‐19 clearly activated brain neurons as shown in PET imaging. Interestingly, a single subcutaneous injection of p3‐Alcβ9‐19 at the dose of 1 mg/kg body weight dramatically restored the viability of AD mouse model neurons. Furthermore, administration of p3‐Alcβ9‐19 (1 mg/kg body weight) improved neuroinflammation triggered by increasing Aβo burden in the brain of the AD mouse model. *In vivo* experiments have confirmed that not only a subcutaneous injection of p3‐Alcβ9‐19 in rodents (Fig EV4A–C) but also a transdermal administration of p3‐Alcβ9‐19 pharmaceutical formulation applied in monkeys (Fig EV4G–I) are sufficient to deliver the peptide into the brain to operate.

**Figure EV6:**
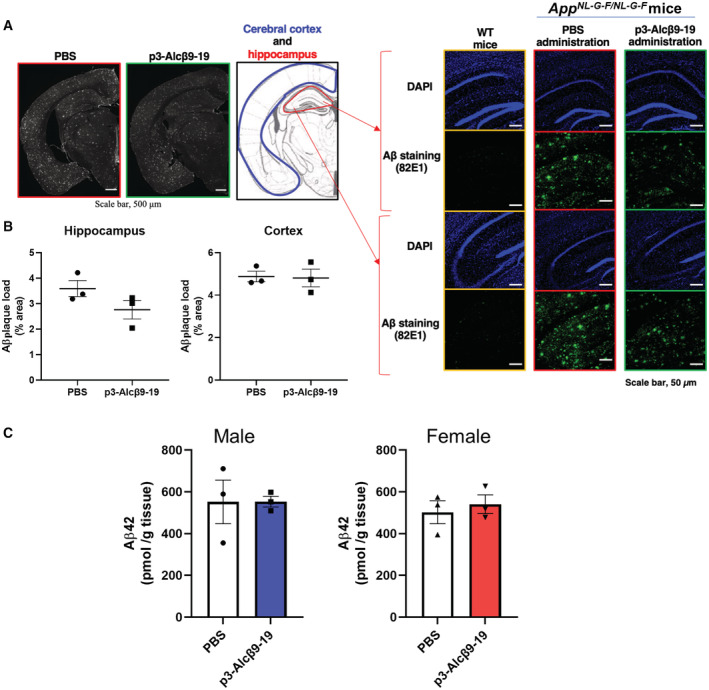
Aβ accumulation and Aβ42 levels in the cerebral cortex and hippocampus in a mouse model of AD with or without subcutaneous administration of p3‐Alcβ9‐19 *App* ^*NL‐G‐F/NL‐G‐F*^ mice (9‐month‐old females) were subcutaneously administered p3‐Alcβ9‐19 (1 mg/kg body weight) or PBS daily for 30 days. The brain sections (20‐μm‐thick) were immunostained with anti‐human Aβ antibody (green) along with DAPI staining (blue). Magnified views of the hippocampus of *APP* ^*NL‐G‐F*^ mice are shown in the panels on the right together with the corresponding area in wild‐type (WT) mice.Aβ plaque‐occupied areas within the regions bordered by blue (cerebral cortex) and red (hippocampus) lines in the hemisphere of the brain (panel A) were quantified by NIH Image J software. After adjusting for the threshold within the indicated area, the percent area above the threshold was measured to obtain the total area that was occupied by Aβ (as displayed in percentage). Slices from three mice with p3‐Alcβ9‐19 or PBS administration were quantified (*n* = 3, one slice per mouse) and statistically analyzed with the unpaired Student's *t*‐test (mean ± SEM, *n* = 3).
*App* ^*NL‐G‐F/NL‐G‐F*^ mice (9‐month‐old males and females) were subcutaneously administered p3‐Alcβ9‐19 (1 mg/kg body weight) or PBS daily for 30 days as described above. The cerebral cortex and hippocampus were dissected and then homogenized. Human Aβ42 levels in the lysates were quantified and the amounts of Aβ42 were compared between AD mice that were or were not administered p3‐Alcβ9‐19. Statistical significance was determined by the unpaired Student's *t*‐test (mean ± SEM, *n* = 3 [3 male (left) or female (right) mice per experimental group]). Data information: Experimental numbers indicate biological replicates. Detailed information including the statistical summary is described in Dataset EV2. Source data are available online for this figure.

In our preliminary study, subcutaneous injection of p3‐Alcβ9‐19 (1 mg/kg body weight) into a mouse model of AD, daily for 30 days, did not cause a significant decrease in brain Aβ load (Fig EV6). Therefore, we would like to re‐emphasize that the function of p3‐Alcβ is to increase neuronal viability and to protect neurons against Aβ‐induced toxicity, and that the target of p3‐Alcβ may not directly be Aβ peptide and Aβ aggregates/plaques. This property of p3‐Alcβ as a druggable candidate for AD therapy is likely to be distinct from immunotherapies that use anti‐Aβ antibodies. Furthermore, the function of p3‐Alcβ to suppress aberrant, Aβo‐induced, Ca^2+^ influx into neurons and to increase neuron mitochondrial activity are novel mechanisms to protect neurons against Aβo‐induced toxicity, which clearly differs from the therapeutic actions of current drugs such as memantine which does not increase mitochondrial activity (Singh *et al*, 2017).

Since endogenous p3‐Alcβ levels may be too low to protect neurons against the increased neurotoxic Aβo burden in the brain of aged individuals, increasing p3‐Alcβ9‐19 levels via peripheral administration has the potential to increase neuronal viability in aged individuals. Furthermore, our recent analysis shows that endogenous p3‐Alcβ levels in the CSF of AD patients are significantly lower than in age‐matched nondemented subjects (Hata *et al*, 2019), which is more obvious in early AD patients (Fig 1). Elderly subjects with low levels of p3‐Alcβ are likely to experience the greater acceleration of AD pathology. Therefore, present results strongly suggest that peripheral administration of p3‐Alcβ9‐19 to AD patients at an early stage, during which endogenous p3‐Alcβ likely starts to decrease, could constitute a promising therapeutic strategy for the restoration of brain function (Fig 6B).

## Materials and Methods

### Animals and mouse primary cultured neurons

All animal studies were conducted in compliance with the ARRIVE guidelines. The mouse study was approved by the Animal Studies Committee of Hokkaido University (#18‐0168). WT C57BL/6J (CLEA, Japan, Inc), Alcβ‐KO (RBRC11514) (Gotoh *et al*, 2020), *App* ^*NL‐F/NL‐F*^ (RBRC06343), and *App* ^*NL‐G‐F/NL‐G‐F*^ (RBRC06344) (Saito *et al*, 2014) mice were housed in specific pathogen‐free (SPF) conditions with a microenvironment vent system (Allentown Inc., Allentown, NJ, USA) under a 12 h/12 h light/dark cycle with free access to food and water. Three to five male or female siblings were housed in each cage; cages were equipped with microbarrier tops. All experimental procedures with *Cynomolgus* monkeys were approved by the Animal Care and Use Committee of Shiga University of Medical Science and were carried out in accordance with approved guidelines (Nishimura *et al*, 2012). Stored frozen pieces of the cerebral cortex of monkeys were used. Sprague–Dawley rats (8‐week‐old) were purchased from Japan SLC Inc. (Hamamatsu, Japan) and housed with their littermates. Each cage had a maximum of three animals, and food and water were available *ad libitum*. All animal protocols and related experiments were approved by the Ethics Committees of the Central Research Laboratory at Hamamatsu Photonics and Hamamatsu University School of Medicine. Mixed mouse cortical and hippocampal neurons were cultured for the indicated day (days *in vitro* in culture/div) using a modification of a previous method (Chiba *et al*, 2014). Briefly, the cortex and hippocampus of mice at embryonic day 15.5 were removed and neurons dissociated in a buffer containing papain (Cat #LS003119, Worthington, Lakewood, NJ, USA) The cells were then cultured at 5 × 10^4^ cells/cm^2^ in Neurobasal Medium (Cat #21103049, Gibco/Thermo Fisher Scientific, Waltham, MA) containing 2% (v/v) B‐27 Supplement (Cat #17504044 Invitrogen, South San Francisco, CA, USA), Glutamax I (4 mM, Cat #35050061, Gibco/Thermo Fisher Scientific), heat‐inactivated horse serum (5% v/v, Cat #26050088, Gibco/Thermo Fisher Scientific), and penicillin plus streptomycin (Cat #35050061, Invitrogen/Thermo Fisher Scientific) in 96‐well microplates (Cat #3595 or Cat #3917, Corning, NY, USA) or Nunc Lab‐Tek II Chambered Coverglass (Cat #Z734853, Nalgene Nunc/Thermo Fisher Scientific) coated with poly‐L‐lysine hydrobromide (Cat #P2636, Sigma‐Aldrich). A half volume of the culture medium was removed and replaced with fresh medium twice a week. To prevent the growth of glial cells, 5‐Fluoro‐2′‐deoxyuridine (Cat #F0503, Sigma‐Aldrich, St. Louis, MO, USA) was added to the cultured medium for the first 3–4 days *in vitro* (div) (Hui *et al*, 2016).

### Synthetic peptides and antibodies

Human p3‐Alcβ37 peptide, which includes the sequence from Val813 to Thr849 of Alcβ (Hata *et al*, 2009), and its partial peptides, p3‐Alcβ1‐11, p3‐Alcβ9‐19, and p3‐Alcβ20‐37, were synthesized and purified to more than 95% purity. Their predicted molecular weights were confirmed by mass spectroscopy, performed at the Peptide Institute (Osaka, Japan). Human p3‐Alcα35 peptide, which includes the sequence from Ala817 to Thr851 of Alcα, was synthesized and purified as described above (Hata *et al*, 2009). Human Aβ42 peptide was synthesized with over 95% purity and purchased from Peptide Institute (Cat #4349‐v). The anti‐p3‐Alcα UT135 antibody has been described previously (Hata *et al*, 2009). A polyclonal rabbit anti‐p3‐Alcβ #854 antibody was raised against an antigen composed of Cys plus the sequence between positions 841 and 849 (C+NSMIPSAAT). This antibody specifically recognizes p3‐Alcβ and does not cross‐react with p3‐Alcα. Commercially available antibodies used in this study are listed in Appendix Table S2.

### Preparation of Aβ42 oligomers and detection by immunoblotting

Aβ42 oligomers were prepared as described previously (Dahlgren *et al*, 2002). Briefly, Aβ42 peptide was dissolved in hexafluoroisopropanol (FUJIFILM Wako Pure Chemicals Corp., Osaka, Japan) to a concentration of 1 mM. After removing hexafluoroisopropanol under vacuum with a SpeedVac system, the peptide was resuspended in dimethyl sulfoxide Hybri‐Max (DMSO) (Sigma‐Aldrich, St. Louis, MO, USA) to a concentration of 5 mM. Ham's F‐12 (phenol red‐free, FUJIFILM Wako Pure Chemicals Corp.) was then added to adjust the final concentration of the peptide to 100 μM. The peptide was then incubated at 4°C for 24 h. Stock solutions (5 mM) of p3‐Alcα and p3‐Alcβ peptides were prepared by dissolving each in DMSO. For the detection of Aβ42, p3‐Alcβ37, and p3‐Alcα35 by immunoblotting, the respective DMSO‐containing peptide solutions were diluted in PBS to 10 μM and incubated at 37°C for 24 h. The peptide solutions were then centrifuged at 20,400 *g* for 10 min at 4°C and the resultant supernatants analyzed with Tris–Tricine polyacrylamide gel SDS electrophoresis followed by immunoblotting with the indicated antibodies.

### Size‐exclusion chromatography (SEC) of p3‐Alcβ37 peptide

p3‐Alcβ37 was dissolved in PBS (10 and 50 μM) and incubated at 37°C. An aliquot (25 or 5 μl, 0.25 nmol each) was directly applied to SEC at the indicated time points (0, 2, 4, and 24 h). SEC was performed on a Shimadzu liquid chromatograph Model LC‐20AT (Kyoto, Japan) using Develosil 100 Diol‐5 (4.6 × 250 mm, Nomura Chemical, Japan) with an isocratic solvent system: 20 mM phosphate buffer containing 0.2 M NaCl (pH 7.0) at a flow rate of 0.5 ml/min, detected at 210 nm. The column was pretreated with excess bovine serum albumin (BSA, Sigma‐Aldrich) to block the nonspecific binding of peptides. Molecular mass was estimated with the marker proteins: thyroglobulin (MW 670,000, Cat #T1001, Sigma‐Aldrich), ovalbumin (MW 44,000, Cat #A5503, Sigma‐Aldrich), RNase A (MW 13,700, Cat #R4875, Sigma‐Aldrich), amyloid β‐protein 1–40 scrambled (MW 4,329.8; Cat #4513‐s, Peptide Institute), and angiotensin II (MW 1,046.2; Cat #4001‐v, Peptide Institute).

### Monitoring of peptide aggregation with Thioflavin T fluorescence

Aβ42 prepared from a stock solution (1 mg/ml Aβ42 in hexafluoroisopropanol) was distributed in aliquots into tubes, followed by removal of hexafluoroisopropanol as described above. Then, Aβ42 was dissolved in DMSO and diluted with PBS (the final concentration of DMSO was 2%). Similarly, the p3‐Alc peptides dissolved in DMSO were diluted with PBS (the final concentration of DMSO was 2%). These peptide solutions (10 μl of 10 μM) were incubated for the indicated time (h) at 37°C. After a 90 μl volume of Thioflavin T (3 μM solution in 100 mM glycine‐NaOH buffer, pH 8.5) was added to the peptide solutions, the fluorescence was measured using EnSpire (PerkinElmer, Waltham, MA, USA) with excitation (430 nm) and emission (485 nm) wavelengths (Ex. 430 nm/Em. 485 nm). In a separate study, we also measured the fluorescence (Ex. 430 nm/Em. 485 nm) of the solutions with a 180 μl volume of Thioflavin T incubated for 12 h at 37°C, which were a mixture of a 10 μl aliquot of the Aβ42 solution (20 μM) with each of a 10 μl aliquot of p3‐Alcα35 or p3‐Alcβ37 solutions with different concentrations (0, 20, 200 μM).

### Effect of p3‐Alcβ peptides on Aβ42‐induced neuronal toxicity and viability

Mouse primary cultured neurons (3 × 10^6^ cells, div 15–20) were treated with Aβ42 oligomers (Aβo) for 24 h in the presence or absence of p3‐Alcβ peptide. Neuronal toxicity and viability were evaluated by MTT (Cat #347‐07621, Dojindo Molecular Technologies, Inc., Kumamoto, Japan), ATP detection (Cat #G9241, CellTiter‐Glo Luminescent Cell Viability Assay; Promega, Madison, WI, USA), and LDH assays (Cat #MK401, LDH Cytotoxicity Detection Kit; Takara Bio, Shiga, Japan). The LDH assay quantifies the level of LDH released into the culture medium, which is a reflection of cellular damage. Generation of ROS was assayed with a Mitochondrial ROS Detection Kit (Cat #701600, Cayman Chemical, Ann Arbor, USA).

### Quantification of intraneuronal Ca^2+^ influx with Fluo 4‐AM

Mouse primary neurons (div 11–14) cultured in Costar 96‐well plates (Cat #3917, Corning) were gently washed with PBS warmed to 37°C and incubated in recording buffer (10 mM HEPES [pH 7.4], 140 mM NaCl, 5.3 mM KCl, 1 mM MgCl_2_, 2 mM CaCl_2_, 30 mM glucose, 0.5 mM sodium pyruvate, 1 mM probenecid) containing 22.8 nM Fluo 4‐AM (Cat #F311, Dojindo Molecular Technologies, Inc. Kumamoto, Japan) and 0.03% Pluronic F‐127 (Cat #59000, Biotium Inc, Fremont, CA, USA) for 30 min, gently washed with warmed PBS, and further incubated for 5 min in the recording buffer (200 μl) with (for the study of Aβo‐ and NMDA administration) or without (for the study of CaCl_2_ administration) 2 mM CaCl_2_. The neurons were administered 5 μl of 82 mM CaCl_2_ (final concentration, 2 mM) when studying CaCl_2_ administration, 11 μl of 100 μM Aβo (final concentration 5.2 μM) in DMEM without L‐glutamate including DMSO (2%) when studying Aβo administration, or 11 μl of 10 mM NMDA (final concentration 52 μM) (Cat #0114, Tocris Bioscience, Bristol UK) in DMEM without L‐glutamate when studying NMDA administration. Continuous fluorescence (excitation 485 nm/emission 518 nm) was measured with EnSpire (PerkinElmer, Waltham, MA, USA). Fluorescence intensity is indicated as a percentage of Δ*F* (*F*_*t*_−*F*_0_)/*F*_0_ × 100 and the area under the curve was subjected to statistical analysis with the Tukey's multiple comparison test or Student's *t*‐test. D‐AP5 and memantine were purchased from Tocris Bioscience (Cat #0106) and FUJIFILM Wako Pure Chemicals Corp (Cat #135‐18311), respectively.

### Photo‐affinity labeling of neuronal proteins in cultured neurons with p3‐Alcβ9‐19 and p3‐Alcβ37, and colocalization with neuronal proteins

The photo‐affinity probe biotin‐X‐p3‐Alcβ9‐19‐K(pBzBz)‐NH_2_, consisting of amino‐terminal biotin plus MiniPEG3 (X)‐conjugated [His‐Arg‐Gly‐His‐Gln‐Pro‐Pro‐Pro‐Glu‐Met‐Ala]‐Lys modified by benzophenone along with C‐terminal amidation (M.W. 2007.3), was synthesized and purified (> 99%) by high‐performance liquid chromatography (HPLC). The biotin‐X‐p3‐Alcβ1‐37‐K(pBzBz)‐KH2 consisting of 37 amino acids of p3‐Alcβ37 was similarly synthesized. Mouse primary neurons (div 23) were incubated with 1 μM of biotin‐X‐p3‐Alcβ9‐19‐K(pBzBz)‐NH_2_ or biotin‐X‐p3‐Alcβ1‐37‐K(pBzBz)‐NH_2_ for 1 h, followed by UV irradiation for 5 min. The neurons were then fixed with 4% paraformaldehyde in PBS, treated with 0.2% Triton X‐100 in PBS, blocked with 4% BSA in PBS, and incubated in primary antibodies for 12 h. This was followed by incubation with an Alexa546‐conjugated anti‐rabbit or anti‐mouse IgG antibody, and streptavidin‐Alexa488 to detect biotin‐X‐p3‐Alcβ‐K(pBzBz)‐NH_2_. Fluorescent images were obtained using an all‐in‐one fluorescence microscope (BZ‐X710, KEYENCE, Osaka, Japan) equipped with a Plan Apochromat 100× oil‐immersion objective (1.4 numerical aperture (NA), Nikon, Tokyo, Japan). The colocalization rates of proteins and p3‐Alcβ were calculated from each frame of images (15,750 μm^2^) of neurons and are indicated as Pearson's *R* value. Independent cell stainings were performed one to three times per cell preparation, and three to five frames were acquired from each well. All values were combined and subjected to statistical analysis with the indicated number of independent biological repeats.

### PET imaging with [^18^F]BCPP‐EF and [^11^C]DPA713

In the rat study, 24 rats (8‐week‐old) were divided into four groups with six animals per group: one group (control) was injected subcutaneously with vehicle (saline), and the three other groups were injected subcutaneously with 1, 3, and 5 mg/kg of p3‐Alcβ9‐19. In the mouse study, 12–14‐month‐old C57BL/6 WT and *App* ^*NL‐F/NL‐F*^ mice were injected subcutaneously with p3‐Alcβ9‐19 (1 mg/kg) or vehicle (saline). The vehicle and peptide were administered just before PET measurements. The [^18^F]BCPP‐EF radiotracer was synthesized using a modified CUPID system (Sumitomo Heavy Industry, Tokyo, Japan), and analyzed by HPLC on a GL‐7400 low‐pressure‐gradient HPLC system (GL Sciences, Inc., Tokyo, Japan) as reported previously (Harada *et al*, 2013). Radioactivity yields, radiochemical purities, and specific radio‐activities of [^18^F]‐BCPP‐EF were 5.1 ± 0.9 (mean ± SD), 99.1 ± 0.7%, and 139.6 ± 37.0 GBq/μmol. The [^11^C]DPA713 radiotracer was synthesized by N‐methylation of the nor‐compound N‐desmethyl‐DPA with ^11^C‐methyl triflates, as reported elsewhere (Boutin *et al*, 2007). Radioactivity yields, the radiochemical purity, and specific radioactivity of [^11^C]DPA713 were 3.5 ± 0.8, more than 99.1 ± 0.9%, and 99.3 ± 32.2 GBq/μmol, respectively. PET measurements were acquired on a high‐resolution animal PET scanner (SHR‐38000, Hamamatsu Photonics, Hamamatsu, Japan) with an axial field of view (FOV) of 330 mm, a transaxial FOV of 108 mm, and a transaxial spatial resolution of 2.3 mm in the center as reported elsewhere (Yamagishi *et al*, 2019). All animals were anesthetized with 1.5–2.0% isoflurane in O_2_ for the duration of the entire imaging experiment. A heat pad was used to control body temperature during PET measurements. The animals were placed in the prone position on a fixation plate and then placed within the gantry hole of the PET scanner. After a 15 min transmission scan utilizing an external ^68^Ge/^68^Ga rod source (67 MBq) for attenuation correction, an 80 min serial emission scan was performed immediately after each injection of [^18^F]BCPP‐EF at a dose of 5 MBq. The tracers were injected intravenously through a cannula inserted into the tail vein. The molar activity of each tracer was above 50 GBq/μmol. No arterial sampling was conducted. The PET data were reconstructed using 3D DRAMA (iteration 2, gamma 0.1) with a Gaussian filter of 1.0 mm full width at half maximum (FWHM), yielding a voxel size of 0.65 × 0.65 × 1.0167 mm for the reconstructed images. To obtain anatomical information, X‐ray CT scans were performed before the PET measurement, using a ClairvivoCT (Shimadzu Corporation, Kyoto, Japan) (Yamagishi *et al*, 2019). Using PMOD image analysis software (version 3.7; PMOD Technologies Ltd, Zurich, Switzerland), the SUVR for [^18^F]BCPP‐EF binding was estimated by dividing the target SUV by the cerebellar SUV (Tsukada *et al*, 2014). The SUV was calculated as the measured radioactivity divided by the ratio of the total injected dose to the mouse body weight. As described elsewhere (Hosoya *et al*, 2017), elliptical ROIs ranging from 12 to 24 mm^2^ were placed over the frontal cortex, caudate putamen, and hippocampus by referring to the X‐ray CT images (Yamagishi *et al*, 2019). A one‐way analysis of variance (ANOVA) was applied to compare SUVR levels in brain regions among the groups. The significance level was set at *P* < 0.05 with Bonferroni correction for multiple comparisons.

### Transdermal microporation of p3‐Alcβ in *Rhesus* monkey

The transdermal delivery device, PassPort System (PS), was provided by PassPort Technologies, Inc (San Diego, CA, USA). The patches to stick on the skin contained different doses of p3‐Alcβ9‐19 peptide and vehicle (saline) within the matrix were prepared in PassPort Technologies, Inc, and each patch in the aluminum laminated pouch was packed with a desiccant until use. The skin for the patch was exposed by shaving the hair of the monkey using an electric shaver 1 day before the p3‐Alcβ9‐19 administration. Transdermal microporation was applied with a condition at 400 density and 4 mJ/filament, followed by the application of the patch on the skin with a transdermal area of 0.5 cm^2^. Immediately after the patch application, the PET scans with [^18^F]BCPP‐EF were conducted during 90 min following intravenous injection of [^18^F]BCPP‐EF as described above.

### ELISA for p3‐Alcβ9‐19 quantification

A polyclonal rabbit antibody was raised against p3‐Alcβ9‐19 containing an amino‐terminal Cys residue (C+HRGHQPPPEMA) and was conjugated to bovine thyroglobulin. IgG was purified with antigen‐coupled resin and conjugated to biotin. Horseradish peroxidase‐conjugated streptavidin was purchased from Amersham/GE Healthcare (Cat #RPN1051, Little Chalfont, UK), and the tetramethyl benzidine (TMB) microwell peroxidase substrate system was obtained from SeraCare Life Sciences Inc. (Cat #5120‐0075, Milford, MA, USA). Mice were then anesthetized with 1% isoflurane, CSF was collected from the cisterna magna as described previously (Liu & Duff, 2008), and mice were then sacrificed. To quantify p3‐Alcβ9‐19 levels in mouse CSF and plasma, the samples were diluted in buffer A (PBS containing 1% BSA and 0.05% Tween‐20). Using this polyclonal antibody, we developed a sELISA system to quantify p3‐Alcβ9‐19 levels in the range of 25–200 pg/ml. The sensitivity of this ELISA is equivalent to that of other sELISA systems used to quantify p3‐Alcβ37 and p3‐Alcβ40 (Hata *et al*, 2019). Antiserum diluted 1:10,000 was used as a capture antibody in the sELISA. The antibody was affinity‐purified with antigen‐coupled resin, and biotin‐labeled IgG was then used as the detection antibody. No reaction occurred with p3‐Alcβ37 (Fig EV4A), and the addition of 1,000 pg/ml p3‐Alcβ37 did not compete with antibody binding to 0–200 pg/ml p3‐Alcβ9‐19, indicating that this sELISA could quantitatively measure p3‐Alcβ9‐19 in body fluids, even in the presence of endogenous p3‐Alcβ.

### Extraction and quantification of Aβ and p3‐Alcβ from monkey brain

Quantifications of Aβ40 and Αβ42 in temporal cortex tissue from *Cynomolgus* monkeys were performed with sELISA kits specific for Aβ40 (Cat #292‐62301, FUJIFILM Wako Pure Chemicals Corp.) and Aβ42 (Cat #296‐62401, FUJIFILM Waco Pure Chemicals Corp.) as described previously (Nishimura *et al*, 2012). In a separate study, temporal cortex tissue was homogenized in eight volumes of Tris‐buffered saline (20 mM Tris–HCl [pH 7.6], 137 mM NaCl) containing a protease inhibitor cocktail (5 μg/ml chymostatin, 5 μg/ml leupeptin, and 5 μg/ml pepstatin A) with 30 strokes of a Dounce homogenizer and centrifuged at 180,000 × *g* for 20 min at 4°C. Since the supernatant contained a low level of p3‐Alcβ peptides that were below the limit of detection, the precipitate was further homogenized in one volume of 6 M guanidine chloride, sonicated twice for 10 s each, and allowed to stand for 1 h at room temperature. The samples were then centrifuged at 180,000 × *g* for 20 min at 4°C. The supernatant was diluted 12‐times in PBS containing 1% (w/v) BSA and 0.05% (v/v) Tween‐20 and then assayed with a sELISA specific for p3‐Alcβ37 and p3‐Alcβ40, as described previously (Hata *et al*, 2019).

### Detection and measurement of amyloid plaques in mouse brain

*App* ^*NL‐G‐F/NL‐G‐F*^ mice (9‐month‐old females) were subcutaneously administered p3‐Alcβ9‐19 (1 mg/kg body weight) or PBS daily for 30 days. The mice brains were fixed and cut into 20‐μm‐thick coronal slices. The brain sections were immunostained with a mouse monoclonal anti‐human Aβ antibody (82E1) (IBL), and the localization of Aβ was visualized with an Alexa Fluor 488‐conjugated donkey anti‐mouse IgG secondary antibody (green) (Invitrogen). Nuclei were counter‐stained with DAPI (blue). The sections were viewed with a BZ‐X710 microscope (Keyence).

### Extraction and quantification of Aβ from mouse brain

*App* ^*NL‐G‐F/NL‐G‐F*^ mice (9‐month‐old males and females) were subcutaneously administered p3‐Alcβ9‐19 (1 mg/kg body weight) or PBS daily for 30 days. The cerebral cortex and hippocampus were dissected and then homogenized on ice for 30 strokes with a Dounce homogenizer in a 4‐fold volume of TBS (20 mM Tris–HCl, pH 7.4 containing 137 mM NaCl) and protease inhibitor cocktail (PIC) (5 μg/ml chymostatin, 5 μg/ml leupeptin, and 5 μg/ml pepstatin). The lysate was centrifuged at 200,000 × *g* for 20 min at 4°C with a TLA 100.4 rotor (Beckman Coulter Life Science). The resultant precipitate was further homogenized in a 9‐fold volume of TBS with a Dounce homogenizer for 30 strokes and was then centrifuged at 100,000 × *g* for 20 min at 4°C with a TLA 55 rotor (Beckman Coulter Life Science). The pellet was dissolved in an equal volume of 6 M guanidine‐HCl solution in 50 mM Tris–HCl (pH 7.6) with sonication (1 s with a 1 s interval of 17 cycles) and left to stand for 1 h at room temperature. The sample was then centrifuged at 130,000 × *g* for 20 min, and the supernatant was used for Aβ42 assays. Human Aβ42 levels were quantified by sandwich ELISA (sELISA) as previously described (Honda *et al*, 2023).

### Cohort information and quantification of CSF biomarkers

We stratified 131 patients whose CSF was collected for diagnostic purposes at Niigata University and related facilities by AT (N) classification according to CSF biomarkers independent of clinical diagnosis. The CSF concentration of Aβ42, p‐tau181, and total tau (t‐tau) was examined at Niigata University, and the cut‐off value for Aβ42, p‐tau181, and t‐tau was described previously (Kasuga *et al*, 2022). The Ethics Committee of Niigata University approved this study (2018‐0409). Participants gave informed consent to participate in the study before taking part. The CSF concentration of p3‐Alcβ37 was quantified with ELISA system as described previously (Hata *et al*, 2019). The Ethics Committee of Hokkaido University approved this study (2021‐003).

### Statistical analysis

Statistical differences were assessed using the Student's *t*‐test or one‐way ANOVAs combined with the Tukey–Kramer *post hoc* test and Dunnett's test or Bonferroni's test for multiple comparisons (GraphPad Prism software, version 9.4.0). *P*‐values < 0.05 were considered significant.

## Author contributions

**Saori Hata:** Formal analysis; funding acquisition; validation; investigation; visualization; writing – original draft; project administration; writing – review and editing. **Haruka Saito:** Formal analysis; validation; investigation; visualization; writing – original draft; project administration; writing – review and editing. **Takeharu Kakiuchi:** Formal analysis; investigation; visualization. **Dai Fukumoto:** Formal analysis; investigation; visualization. **Shigeyuki Yamamoto:** Formal analysis; investigation; visualization. **Kensaku Kasuga:** Resources; formal analysis. **Ayano Kimura:** Formal analysis; investigation; visualization; project administration. **Koichi Moteki:** Investigation. **Ruriko Abe:** Resources; formal analysis; investigation. **Shungo Adachi:** Formal analysis; investigation. **Shoich Kinoshita:** Investigation. **Kumiko Yoshizawa‐Kumagaye:** Resources; supervision; methodology; writing – review and editing. **Hideki Nishio:** Resources. **Takashi Saito:** Resources. **Takaomi C Saido:** Resources. **Tohru Yamamoto:** Resources; project administration. **Masaki Nishimura:** Resources; methodology. **Hidenori Taru:** Project administration; writing – review and editing. **Yuriko Sobu:** Validation; project administration; writing – review and editing. **Hiroyuki Ohba:** Formal analysis; investigation. **Shingo Nishiyama:** Formal analysis; investigation. **Norihiro Harada:** Formal analysis; investigation. **Takeshi Ikeuchi:** Resources; formal analysis. **Hideo Tsukada:** Resources; supervision; methodology. **Yasuomi Ouchi:** Conceptualization; data curation; formal analysis; supervision; funding acquisition; investigation; visualization; writing – original draft; project administration; writing – review and editing. **Toshiharu Suzuki:** Conceptualization; data curation; supervision; funding acquisition; validation; visualization; writing – original draft; project administration; writing – review and editing.

## Disclosure and competing interests statement

The authors declare that they have no conflict of interest.

## For more information

1. https://www.alzint.org/resource/world‐alzheimer‐report‐2018/ is a link to the World Alzheimer's Report (2018). The World Alzheimer's Report (2018) highlights an urgent need for increased and sustainable funding for dementia research while also addressing the complexities around dementia research.
2. https://www.alzforum.org is a website to accelerate research into Alzheimer's disease and related disorders and to promote networking between researchers.

## Supporting information



Appendix

Expanded View Figures PDF

Dataset EV1

Dataset EV2

Source Data for Expanded View

PDF+

Source Data for Figure 1

Source Data for Figure 2

Source Data for Figure 3

Source Data for Figure 4

Source Data for Figure 5

## Data Availability

Data in this article will be shared on reasonable request from any qualified investigator.

## References

[emmm202217052-bib-0001] Araki Y , Tomita S , Yamaguchi H , Miyagi N , Sumioka A , Kirino Y , Suzuki T (2003) Novel cadherin‐related membrane proteins, Alcadeins, enhance the X11‐like protein‐mediated stabilization of amyloid β‐protein precursor metabolism. J Biol Chem 278: 49448–49458 12972431 10.1074/jbc.M306024200

[emmm202217052-bib-0002] Araki Y , Miyagi N , Kato N , Yoshida T , Wada S , Nishimura M , Komano H , Yamamoto T , De Strooper B , Yamamoto K *et al* (2004) Coordinated metabolism of Alcadein and amyloid β‐protein precursor regulates FE65‐dependent gene transactivation. J Biol Chem 279: 24343–24354 15037614 10.1074/jbc.M401925200

[emmm202217052-bib-0003] Araki Y , Kawano T , Taru H , Saito Y , Wada S , Miyamoto K , Kobayashi H , Ishikawa HO , Ohsugi Y , Yamamoto T *et al* (2007) The novel cargo Alcadein induces vesicle association of kinesin‐1 motor components and activates axonal transport. EMBO J 26: 1475–1486 17332754 10.1038/sj.emboj.7601609 PMC1829376

[emmm202217052-bib-0004] Benilova I , Karran E , De Strooper B (2012) The toxic Aβ oligomer and Alzheimer's disease: an emperor in need of clothes. Nat Neurosci 15: 349–357 22286176 10.1038/nn.3028

[emmm202217052-bib-0005] Boraxbekk CJ , Ames D , Kochan NA , Lee T , Thalamuthu A , Wen W , Armstrong NJ , Kwok JBJ , Schofield PR , Reppermund S *et al* (2015) Investigating the influence of KIBRA and CLSTN2 genetic polymorphisms on cross‐sectional and longitudinal measures of memory performance and hippocampal volume in older individuals. Neuropsychologia 78: 10–17 26415670 10.1016/j.neuropsychologia.2015.09.031

[emmm202217052-bib-0006] Boutin H , Chauveau F , Thominiaux C , Grégoire M‐C , James ML , Trebossen R , Hantraye P , Dollé F , Tavitian B , Kassiou M (2007) 11C‐DPA‐713: a novel peripheral benzodiazepine receptor PET ligand for *in vivo* imaging of neuroinflammation. J Nucl Med 48: 573–581 17401094 10.2967/jnumed.106.036764

[emmm202217052-bib-0007] Cheignon C , Tomas M , Bonnefont‐Rousselot D , Faller P , Hureau C , Collin F (2018) Oxidative stress and the amyloid beta peptide in Alzheimer's disease. Redox Biol 14: 450–464 29080524 10.1016/j.redox.2017.10.014 PMC5680523

[emmm202217052-bib-0008] Chiba K , Araseki M , Nozawa K , Furukori K , Araki Y , Matsushima T , Nakaya T , Hata S , Saito Y , Uchida S *et al* (2014) Quantitative analysis of APP axonal transport in neurons: role of JIP1 in enhanced APP anterograde transport. Mol Biol Cell 25: 3569–3580 25165140 10.1091/mbc.E14-06-1111 PMC4230617

[emmm202217052-bib-0009] Dahlgren KN , Manelli AM , Stine WBL Jr , Baker LK , Krafft GA , Jo LaDu M (2002) Oligomeric and fibrillar species of amyloid‐β peptides differentially affect neuronal viability. J Biol Chem 277: 32046–32053 12058030 10.1074/jbc.M201750200

[emmm202217052-bib-0010] De Strooper B , Saftig P , Craessaerts K , Vanderstichele H , Guhde G , Annaert W , Von Figura K , Van Leuven F (1998) Deficiency of presenilin‐1 inhibits the normal cleavage of amyloid precursor protein. Nature 391: 387–390 9450754 10.1038/34910

[emmm202217052-bib-0011] Forsberg M , Seth H , Björefeldt A , Lyckenvik T , Andersson M , Wasling P , Zetterberg H , Hanse E (2019) Ionized calcium in human cerebrospinal fluid and its influence on intrinsic and synaptic excitability of hippocampal pyramidal neurons in the rat. J Neurochem 149: 452–470 30851210 10.1111/jnc.14693

[emmm202217052-bib-0012] Gotoh N , Saito Y , Hata S , Saito H , Ojima D , Murayama C , Shigeta M , Abe T , Konno D , Matsuzaki F *et al* (2020) Amyloidogenic processing of amyloid β protein precursor (APP) is enhanced in the brains of alcadein α‐deficient mice. J Biol Chem 295: 9650–9662 32467230 10.1074/jbc.RA119.012386 PMC7363152

[emmm202217052-bib-0013] Harada N , Nishiyama S , Kanazawa M , Tsukada H (2013) Development of novel PET probes, [^18^F]BCPP EF, [^18^F]BCPP‐BF, and [^11^C]BCPP‐EM for mitochondrial complex 1 imaging in the living brain. J Labelled Comp Radiopharm 56: 553–561 24285187 10.1002/jlcr.3056

[emmm202217052-bib-0014] Hardingham GE , Bading H (2010) Synaptic versus extrasynaptic NMDA receptor signaling: implications for neurodegenerative disorders. Nat Rev Neurosci 11: 682–696 20842175 10.1038/nrn2911 PMC2948541

[emmm202217052-bib-0015] Hardy J , Selkoe DJ (2002) The amyloid hypothesis of Alzheimer's disease: progress and problems on the road to therapeutics. Science 297: 353–356 12130773 10.1126/science.1072994

[emmm202217052-bib-0016] Hata S , Fujishige S , Araki Y , Kato N , Araseki M , Nishimura M , Hartmann D , Saftig P , Fahrenholz F , Taniguchi M *et al* (2009) Alcadein cleavages by amtloid β‐precursor protein (APP) α‐ and γ‐secretases generate small peptides, p3‐Alcs, indicating Alzheimer disease‐related γ‐secretase dysfunction. J Biol Chem 284: 36024–36033 19864413 10.1074/jbc.M109.057497 PMC2794718

[emmm202217052-bib-0017] Hata S , Fujishige S , Araki Y , Taniguchi M , Urakami K , Peskind E , Akatsu H , Araseki M , Yamamoto K , Martins RN *et al* (2011) Alternative processing of γ‐secretase substrates in common forms of mild cognitive impairment and Alzheimer's disease: evidence for γ‐secretase dysfunction. Ann Neurol 69: 1026–1031 21681798 10.1002/ana.22343 PMC3306841

[emmm202217052-bib-0018] Hata S , Omori C , Kimura A , Saito H , Kimura N , Gupta V , Pedrini S , Hone E , Chatterjee P , Taddei K *et al* (2019) Decrease in p3‐Alcβ37 and p3‐Alcβ40, products of Alcadein β generated by γ‐secretase cleavages, in aged monkeys and patients with Alzheimer's disease. Alzheimers Dement (NY) 5: 740–750 10.1016/j.trci.2019.09.015 PMC6854065 31754625

[emmm202217052-bib-0019] Hintsch G , Zurlinden A , Meskenaite V , Steuble M , Fink‐Widmer K , Kinter J , Sonderegger P (2002) The calsyntenins—a family of postsynaptic membrane proteins with distinct neuronal expression patterns. Mol Cell Neurosci 21: 393–409 12498782 10.1006/mcne.2002.1181

[emmm202217052-bib-0020] Honda K , Saito Y , Saito H , Toyooda M , Abe R , Saito T , Saido TC , Michikawa M , Taru H , Sobu Y *et al* (2023) Accumulation of amyloid‐β in the brain of mouse models of Alzheimer's disease is modified by altered gene expression in the presence of human apoE isoforms during aging. Neurobiol Aging 123: 63–74 36638682 10.1016/j.neurobiolaging.2022.12.003

[emmm202217052-bib-0021] Hosoya T , Fukumoto D , Kakiuchi T , Nishiyama S , Yamamoto S , Ohba H , Tsukada H , Ueki T , Sato K , Ouchi Y (2017) *In vivo* TSPO and cannabinoid receptor type 2 availability early in post‐stroke neuroinflammation in rats: a positron emission tomography study. J Neuroinflammation 14: 69 28356120 10.1186/s12974-017-0851-4 PMC5372312

[emmm202217052-bib-0022] Hui CW , Zhang Y , Herrup K (2016) Non‐neuronal cells are required to mediate the effects of neuroinflammation: results from a neuron‐enriched culture system. PLoS One 11: e0147134 26788729 10.1371/journal.pone.0147134 PMC4720438

[emmm202217052-bib-0023] Jack CR Jr , Bennett DA , Blennow K , Carrillo MC , Dunn B , Haeberlein SB , Holtzman DM , Jagust W , Jessen F , Karlawish J *et al* (2018) NIA‐AA research framework: toward a biological definition of Alzheimer's disease. Alzheimers Dement 14: 535–562 29653606 10.1016/j.jalz.2018.02.018 PMC5958625

[emmm202217052-bib-0024] Kasuga K , Kikuchi M , Tsukie T , Suzuki K , Ihara R , Iwata A , Hara N , Miyashita A , Kuwano R , Iwatsubo T *et al* (2022) Different AT(N) profiles and clinical progression classified by two different N markers using total tau and neurofilament light chain in cerebrospinal fluid. BMJ Neurol Open 4: e000321 10.1136/bmjno-2022-000321 PMC9379489 36046332

[emmm202217052-bib-0025] Kawano T , Araseki M , Araki Y , Kinjo M , Yamamoto T , Suzuki T (2012) A small peptide sequence is sufficient for initiating kinesin‐1 activation through part of TPR region of KLC1. Traffic 13: 834–848 22404616 10.1111/j.1600-0854.2012.01350.x

[emmm202217052-bib-0026] Konecna A , Frischknecht R , Kinter J , Ludwig A , Steuble M , Meskenaite V , Indermühle M , Engel M , Cen C , Mateos J‐M *et al* (2006) Calsyntenin‐1 docks vesicular cargo to kinesin‐1. Mol Biol Cell 17: 3651–3663 16760430 10.1091/mbc.E06-02-0112 PMC1525238

[emmm202217052-bib-0027] Lipina TV , Prasad T , Yokomaku D , Luo L , Connor SA , Kawabe H , Wang YT , Brose N , Roder JC , Craig AM (2016) Cognitive deficits in calsyntenin‐2‐deficient mice associated with reduced GABAergic transmission. Neuropsychopharmacology 41: 802–810 26171716 10.1038/npp.2015.206 PMC4707826

[emmm202217052-bib-0028] Liu L , Duff K (2008) A technique for serial collection of cerebrospinal fluid from the cisterna magna in mouse. J Vis Exp 21: 960 10.3791/960 PMC2762909 19066529

[emmm202217052-bib-0029] Lu Z , Wang Y , Chen F , Tong H , Reddy MVVVS , Luo L , Seshadrinathan S , Zhang L , Holthauzen LMF , Craig AM *et al* (2014) Calsyntenin‐3 molecular architecture and interaction with neurexin 1α. J Biol Chem 289: 34530–34542 25352602 10.1074/jbc.M114.606806 PMC4263861

[emmm202217052-bib-0030] Ludwig A , Blume J , Diep T‐M , Yuan J , Mateos JM , Leuthäuser K , Steuble M , Streit P , Sonderegger P (2009) Calsyntenins mediate TGN exit of APP in a kinesin‐1‐dependent manner. Traffic 10: 572–589 19192245 10.1111/j.1600-0854.2009.00886.x

[emmm202217052-bib-0031] McLean CA , Cherny RA , Fraser FW , Fuller SJ , Smith MJ , Beyreuther K , Bush AI , Masters CL (1999) Soluble pool of Aβ amyloid as a determinant of severity of neurodegeneration in Alzheimer's disease. Ann Neurol 46: 860–866 10589538 10.1002/1531-8249(199912)46:6<860::aid-ana8>3.0.co;2-m

[emmm202217052-bib-0032] Nishimura M , Nakamura S , Kimura N , Liu L , Suzuki T , Tooyama I (2012) Age‐related modulation of γ‐secretase activity in non‐human primate brains. J Neurochem 123: 21–28 22817324 10.1111/j.1471-4159.2012.07884.x

[emmm202217052-bib-0033] Ono N , Iibuchi T , Todo H , Itakura S , Adachi H , Sugibayashi K (2022) Enhancement of skin permeation of fluorescein isothiocyanate‐dextran 4 kDa (FD4) and insulin by thermalporation. Eur J Pharm Sci 170: 106096 34929301 10.1016/j.ejps.2021.106096

[emmm202217052-bib-0034] Pettem KL , Yokomaku D , Luo L , Linhoff MW , Prasad T , Connor SA , Siddiqui TJ , Kawabe H , Chen F , Zhang L *et al* (2013) The specific α‐neurexin interactor calsyntenin‐3 promotes excitatory and inhibitory synapse development. Neuron 80: 113–128 24094106 10.1016/j.neuron.2013.07.016 PMC3821696

[emmm202217052-bib-0035] Ridge PG , Kauwe JSK (2018) Mitochondria and Alzheimer's disease: the role of mitochondrial genetic variation. Curr Genet Med Rep 6: 1–10 29564191 10.1007/s40142-018-0132-2 PMC5842281

[emmm202217052-bib-0036] Saito T , Matsuba Y , Mihira N , Takano J , Nilsson P , Itohara S , Iwata N , Saido TC (2014) Single App knock‐in mouse models of Alzheimer's disease. Nat Neurosci 17: 661–663 24728269 10.1038/nn.3697

[emmm202217052-bib-0037] Singh N , Hroudova J , Fišar Z (2017) *In vitro* effect of cognitives and nootropics on mitochondrial respiration and monoamine oxidase activity. Mol Neurobiol 54: 5894–5904 27660276 10.1007/s12035-016-0121-y

[emmm202217052-bib-0038] Sobu Y , Furukori K , Chiba K , Nairn AC , Kinjo M , Hata S , Suzuki T (2017) Phosphorylation of multiple sites within an acidic region of Alcadein α is required for kinesin‐1 association and Golgi exit of Alcadein α cargo. Mol Biol Cell 28: 3844–3856 29093024 10.1091/mbc.E17-05-0301 PMC5739299

[emmm202217052-bib-0039] Takei N , Sobu Y , Kimura A , Urano S , Piao Y , Araki Y , Taru H , Yamamoto T , Hata S , Nakaya T *et al* (2015) Cytoplasmic fragment of Alcadein α generated by regulated intramembrane proteolysis enhances amyloid β‐protein precursor (APP) transport into the late secretory pathway and facilitates APP cleavage. J Biol Chem 290: 987–995 25406318 10.1074/jbc.M114.599852 PMC4294525

[emmm202217052-bib-0040] Thinakaran G , Koo EH (2008) Amyloid precursor protein trafficking, processing, and function. J Biol Chem 283: 29615–29619 18650430 10.1074/jbc.R800019200 PMC2573065

[emmm202217052-bib-0041] Tsukada H , Nishiyama S , Fukumoto D , Kanazawa M , Harada N (2014) Novel PET probes ^18^F‐BCPP‐EF and ^18^F‐BCPP‐BF for mitochondrial complex I: a PET study in comparison with ^18^F‐BMS‐747158‐02 in rat brain. J Nucl Med 55: 473–480 24470629 10.2967/jnumed.113.125328

[emmm202217052-bib-0042] Tymianski M , Charlton MP , Carlen PL , Tator CH (1993) Source specificity of early calcium neurotoxicity in cultured embryonic spinal neurons. J Neurosci 13: 2085–2104 8097530 10.1523/JNEUROSCI.13-05-02085.1993 PMC6576557

[emmm202217052-bib-0043] Um JW , Pramanik G , Ko JS , Song M‐Y , Lee D , Kim H , Park K‐S , Südhof TC , Tabuchi K , Ko J (2014) Calsyntenins function as synaptogenic adhesion molecules in concert with neurexins. Cell Rep 6: 1096–1109 24613359 10.1016/j.celrep.2014.02.010 PMC4101519

[emmm202217052-bib-0044] Vagnoni A , Perkinton MS , Gray EH , Francis PT , Noble W , Miller CC (2012) Calsyntenin‐1 mediates axonal transport of the amyloid precursor protein and regulates Aβ production. Hum Mol Genet 21: 2845–2854 22434822 10.1093/hmg/dds109 PMC3373235

[emmm202217052-bib-0045] Vassar R , Bennett BD , Babu‐Khan S , Kahn S , Mendiaz EA , Denis P , Teplow DB , Ross S , Amarante P , Loeloff R *et al* (1999) Beta‐secretase cleavage of Alzheimer's amyloid precursor protein by the transmembrane aspartic protease BACE. Science 286: 735–741 10531052 10.1126/science.286.5440.735

[emmm202217052-bib-0046] Walsh DM , Hartley DM , Kusumoto Y , Fezoui Y , Condron MM , Lomakin A , Benedek GB , Selkoe DJ , Teplow DB (1999) Amyloid β‐protein fibrillogenesis. Structure and biological activity of protofibrillar intermediates. J Biol Chem 274: 25945–25952 10464339 10.1074/jbc.274.36.25945

[emmm202217052-bib-0047] World Alzheimer's Report (2018) The state of the art of dementia research: new frontiers. London: Alzheimer's Disease International. https://www/alzint.org/resource/world-alzheimer-report-2018

[emmm202217052-bib-0048] Yamagishi S , Iga Y , Nakamura M , Takizawa C , Fukumoto D , Kakiuchi T , Nishiyama S , Ohba H , Tsukada H , Sato K *et al* (2019) Upregulation of cannabinoid receptor type 2, but not TSPO, in senescence‐accelerated neuroinflammation in mice: a positron emission tomography study. J Neuroinflammation 16: 208 31707986 10.1186/s12974-019-1604-3 PMC6842455

[emmm202217052-bib-0049] Zhang S‐J , Steijaert MN , Lau D , Schültz G , Delucinge‐Vivier C , Descombes P , Bading H (2007) Decoding NMDA receptor signaling: identification of genomic programs specifying neuronal survival and death. Neuron 53: 549–562 17296556 10.1016/j.neuron.2007.01.025

