# Supplementary material for: Brain p3‐Alcβ peptide restores neuronal viability impaired by Alzheimer's amyloid β‐peptide
Source: EMBO Mol Med. 2023 Mar 30;15(5):e17052. doi: 10.15252/emmm.202217052 (PMC10165357; doi:10.15252/emmm.202217052)
Supplement: Supplementary file 1 — Appendix [file EMMM-15-e17052-s001.pdf]

**Brain p3-Alcβ peptide restores neuronal viability impaired by Alzheimer's amyloid β-peptide**

Saori Hata, Haruka Saito, Takeharu Kakiuchi, Dai Fukumoto, Shigeyuki Yamamoto, Kensaku Kasuga, Ayano Kimura, Koichi Moteki, Ruriko Abe, Shungo Adachi, Shoich Kinoshita, Kumiko Yoshizawa-Kumagaye, Hideki Nishio, Takashi Saito, Takaomi C. Saido, Tohru Yamamoto, Masaki Nishimura, Hidenori Taru, Yuriko Sobu, Hiroyuki Ohba, Shingo Nishiyama, Norihiro Harada, Takeshi Ikeuchi, Hideo Tsukada, Yasuomi Ouchi, and Toshiharu Suzuki.

Appendix table of contents:
-Index: page 1
-Appendix Table S1: page 2
-Appendix Table S2: page 3

**Appendix tables**

**Appendix Table S1. Subject information and biomarker levels in CSF**

| Group | Age (y) | Female % | Aβ42 (pg/mL) | p-tau181 (pg/mL) | t-tau (pg/mL) | p3-Alcβ37 (pg/mL) |
|---|---|---|---|---|---|---|
| A- (n=36) | 74.67 ± 10.91 | 58.33 | 453.1 ± 92.75 | 27.47 ± 11.77 | 67.22 ± 61.83 | 3928 ± 1372 |
| A+T-N- (n=51) | 74.88 ± 8.536 | 60.78 | 219.6 ± 80.9 | 20.77 ± 6.272 | 39.16 ± 19.27 | 1889 ± 858.7 |
| A+T+N- (n=30) | 74.87 ± 8.361 | 63.33 | 196.4 ± 78.26 | 41.67 ± 9.927 | 64.43 ± 24.66 | 2899 ± 1314 |
| A+T+N+ (n=14) | 76.57 ± 10.65 | 57.14 | 245 ± 77.52 | 72.01 ± 29.67 | 282.1 ± 175.3 | 3808 ± 1515 |

Groups were classified using the cut-off values of CSF biomarkers based on Gaussian mixture models (GMMs) of the previous report (Kasuga *et al*, 2022). Cut-off values are 359.6 pg/mL for Aβ42 (A+ indicates <359.6 pg/mL), 30.6 pg/mL for p-tau181 (T+ indicates >30.6 pg/mL), and 105.3 pg/mL for t-tau (>105.3 pg/mL) respectively.

**Appendix Table S2. Purchased antibodies used in this study**

| Antibody | Supplier | Catalogue # | RRID | Host | Dilution |
|---|---|---|---|---|---|
| Monoclonal anti-tubulin β3 (TUJ1) | BioLegend (San Diego, CA, USA) | 801201 | AB_2313773 | Mouse | 1:1000 |
| Monoclonal anti-Aβ (82E1) | IBL (Fujioka, Japan) | 10323 | AB_10707424 | Mouse | 1:500 |
| Monoclonal anti-synaptophysin (D-4) | Santa Cruz Biotechnology, (Dallas, TX, USA) | sc-17750 | AB_628311 | Mouse | 1:1000 |
| Monoclonal anti-GluN2B | BD Biosciences (San Jose, CA, USA) | 610416 | AB_397796 | mouse | 1:1000 |
| Alexa Fluor 488-conjugated donkey anti-mouse IgG | Invitrogen/Thermo Fisher Scientific (Waltham, MA, USA) | A-21202 | AB_141607 | Donkey | 1:500 |
| Alexa Fluor 546-conjugated goat anti-mouse IgG | Invitrogen/Thermo Fisher Scientific (Waltham, MA, USA) | A-11003 | AB_2534071 | Goat | 1:500 |
| Alexa Fluor 488-conjugated donkey anti-rabbit IgG | Abcam (Cambridge, UK) | ab150065 | n/a | Donkey | 1:500 |
| Alexa Fluor 54-conjugated goat anti-rabbit IgG | Invitrogen/Thermo Fisher Scientific (Waltham, MA, USA) | A-11010 | AB_2534077 | Goat | 1:500 |
