# Supplementary material for: Brain p3‐Alcβ peptide restores neuronal viability impaired by Alzheimer's amyloid β‐peptide
Source: EMBO Mol Med. 2023 Mar 30;15(5):e17052. doi: 10.15252/emmm.202217052 (PMC10165357; doi:10.15252/emmm.202217052)
Supplement: Supplementary file 2 — Expanded View Figures PDF [file EMMM-15-e17052-s008.pdf]

Expanded View Figures

Figure EV1. Nonaggregative property of p3-Alc peptides and the effect of p3-Alcβ on the viability of mouse primary cultured neurons.

A The amino acid sequences of human Aβ42, p3-Alcα35, and p3-Alcβ37 (amino acid numbers of Aβ42 are for the APP695 isoform).

B p3-Alc peptides do not aggregate. Synthetic Aβ42, p3-Alcα35, and p3-Alcβ37 peptides (10 μM each) were solubilized in PBS with (+) or without (−) incubation at 37°C for 24 h. The peptides were immunoblotted using anti-Aβ 82E1 (left), anti-p3-Alcα UT135 (middle), and anti-p3-Alcβ #854 (right) antibodies. Protein size markers are shown (kDa).

C p3-Alcβ37 does not form oligomers in PBS. Synthetic p3-Alcβ37 was dissolved in PBS (50 μM) and then incubated at 37°C for the indicated time (0, 2, 4, 24 h), and aliquots (5 μl) were subjected to size-exclusion chromatography (left panel) along with molecular size marker proteins (right). The retention time of marker proteins is indicated on the graph on the right.

D Monitoring of peptide aggregation with Thioflavin T fluorescence. Indicated peptide solutions (10 μl of 10 μM) were incubated for the indicated time (h) at 37°C. Thioflavin T was then added to the peptide solutions, and the fluorescence was measured (Ex. 430 nm/Em. 485 nm). The fluorescence intensity is represented as a relative fluorescent unit (RFU) (mean ± SEM; $n = 8$).

E Aβ42 aggregation in the presence of p3-Alc peptides. Aliquots (10 μl) of Aβ42 solution (20 μM) were combined with the aliquots (10 μl) of p3-Alcα35 or p3-Alcβ37 solutions (0, 20, 200 μM). The assay solutions were incubated for 12 h at 37°C and Thioflavin T was added to measure the fluorescence (Ex 430 nm/Em 485 nm). The fluorescence intensity (RFU) is expressed relative to that of the sample in the absence of p3-Alc peptides (assigned a value of 1.0). Statistical significance was determined by a one-way ANOVA followed by the Tukey's multiple comparisons test (mean ± SEM; $n = 6$).

F Effect of p3-Alcα35 and p3-Alcβ37 on the viability of mouse primary cultured neurons. Wild-type neurons (div 15–20) were incubated for 24 h in the presence (10 μM) or absence (−) of p3-Alcα35 and p3-Alcβ37. Neuronal viability was evaluated using MTT assays and is expressed relative to that of cells cultured in the absence of peptides (assigned a value of 1.0). Statistical analysis was performed using a one-way ANOVA, followed by the Dunnett's multiple comparisons test (mean ± SEM; $n = 10$), and the significant P-value ($P < 0.01$) is indicated on the graph.

G Aβ42 oligomer-induced neurotoxicity and restoration of neuronal viability by p3-Alcβ9-19 and p3-Alcβ37. Wild-type mouse neurons (DIV 16–17) were incubated for 24 h with (+, 2.5 μM (left) and 10 μM (right)) or without (−) Aβ42 oligomers (Aβo) in the presence (9–19, p3-Alcβ9-19; 1–37, p3-Alcβ37) or absence (−) of p3-Alcβ peptide (10 μM). Neuronal viability was evaluated with MTT assays and expressed relative to that of cells cultured in the absence of peptides (assigned a value of 1.0). Statistical analysis was performed using a one-way ANOVA, followed by the Tukey's multiple comparisons test (mean ± SEM; $n = 16$–17), and the significant P-values ($P < 0.05$, $P < 0.01$, $P < 0.0001$) are indicated on the graph.

Data information: Experimental numbers indicate biological replicates. Detailed information including the statistical summary is described in Dataset EV2.
Source data are available online for this figure.

 © 2023 The Authors

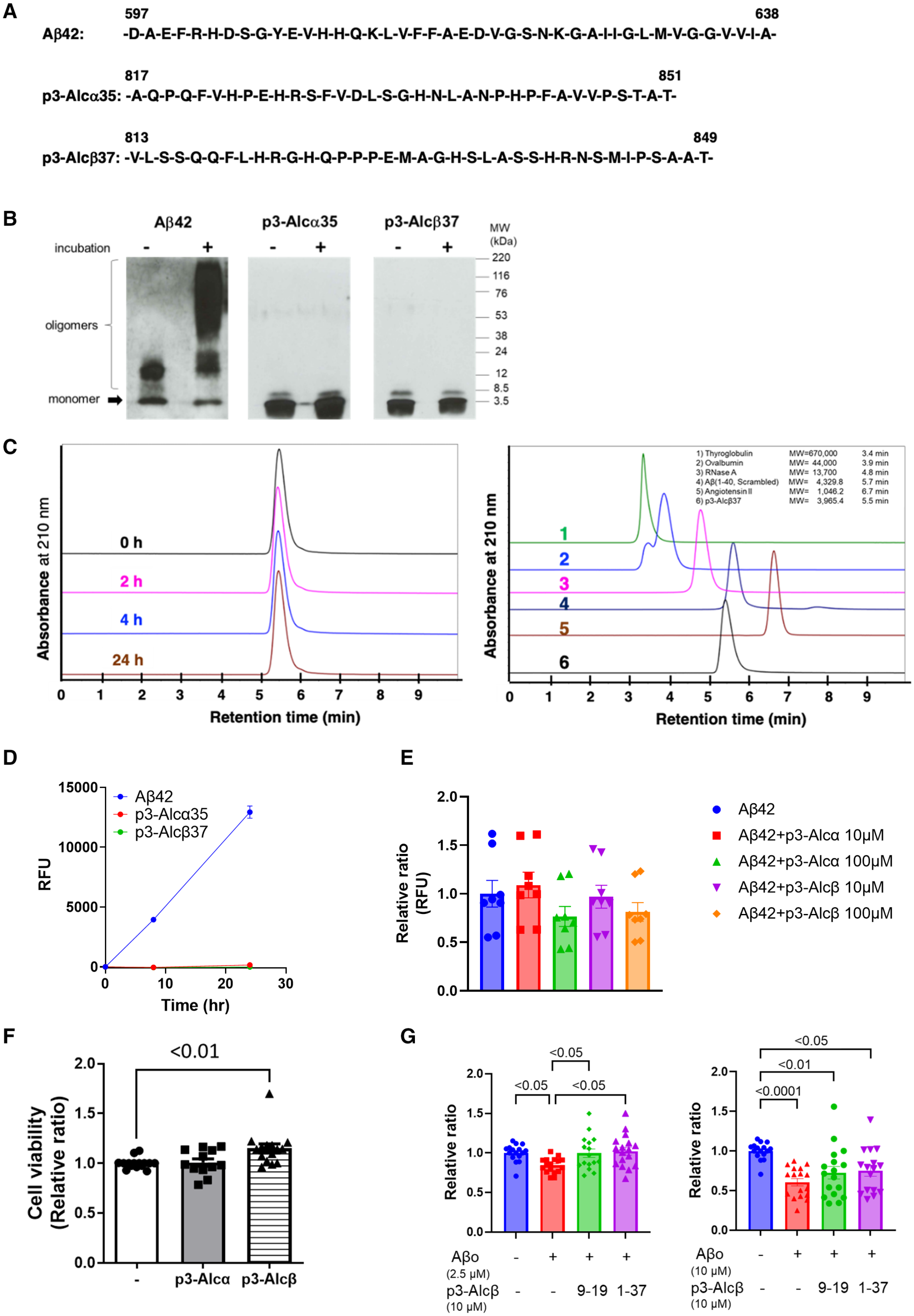
A
597 638
Aβ42: -D-A-E-F-R-H-D-S-G-Y-E-V-H-H-Q-K-L-V-F-F-A-E-D-V-G-S-N-K-G-A-I-I-G-L-M-V-G-G-V-V-I-A-
817 851
p3-Alcα35: -A-Q-P-Q-F-V-H-P-E-H-R-S-F-V-D-L-S-G-H-N-L-A-N-P-H-P-F-A-V-V-P-S-T-A-T-
813 849
p3-Alcβ37: -V-L-S-S-Q-Q-F-L-H-R-G-H-Q-P-P-P-E-M-A-G-H-S-L-A-S-S-H-R-N-S-M-I-P-S-A-A-T-
B
Aβ42 p3-Alcα35 p3-Alcβ37
incubation - + - + - +
MW (kDa)
220 116 76 53 38 24 12 8.5 3.5
oligomers
monomer
C
Absorbance at 210 nm
0 h
2 h
4 h
24 h
Retention time (min)
1) Thyroglobulin MW=670,000 3.4 min
2) Ovalbumin MW= 44,000 3.9 min
3) RNase A MW= 13,700 4.8 min
4) Aβ(1-40, Scrambled) MW= 4,329.8 5.7 min
5) Angiotensin II MW= 1,046.2 6.7 min
6) p3-Alcβ37 MW= 3,965.4 5.5 min
D
RFU
Aβ42
p3-Alcα35
p3-Alcβ37
Time (hr)
E
Relative ratio (RFU)
Aβ42
Aβ42+p3-Alcα 10μM
Aβ42+p3-Alcα 100μM
Aβ42+p3-Alcβ 10μM
Aβ42+p3-Alcβ 100μM
F
Cell viability (Relative ratio)
<0.01
- p3-Alcα p3-Alcβ
G
Relative ratio
<0.05 <0.05 <0.05
Aβo (2.5 μM) - + + +
p3-Alcβ (10 μM) - - 9-19 1-37
<0.05 <0.01 <0.0001
Aβo (10 μM) - + + +
p3-Alcβ (10 μM) - - 9-19 1-37

Figure EV1.

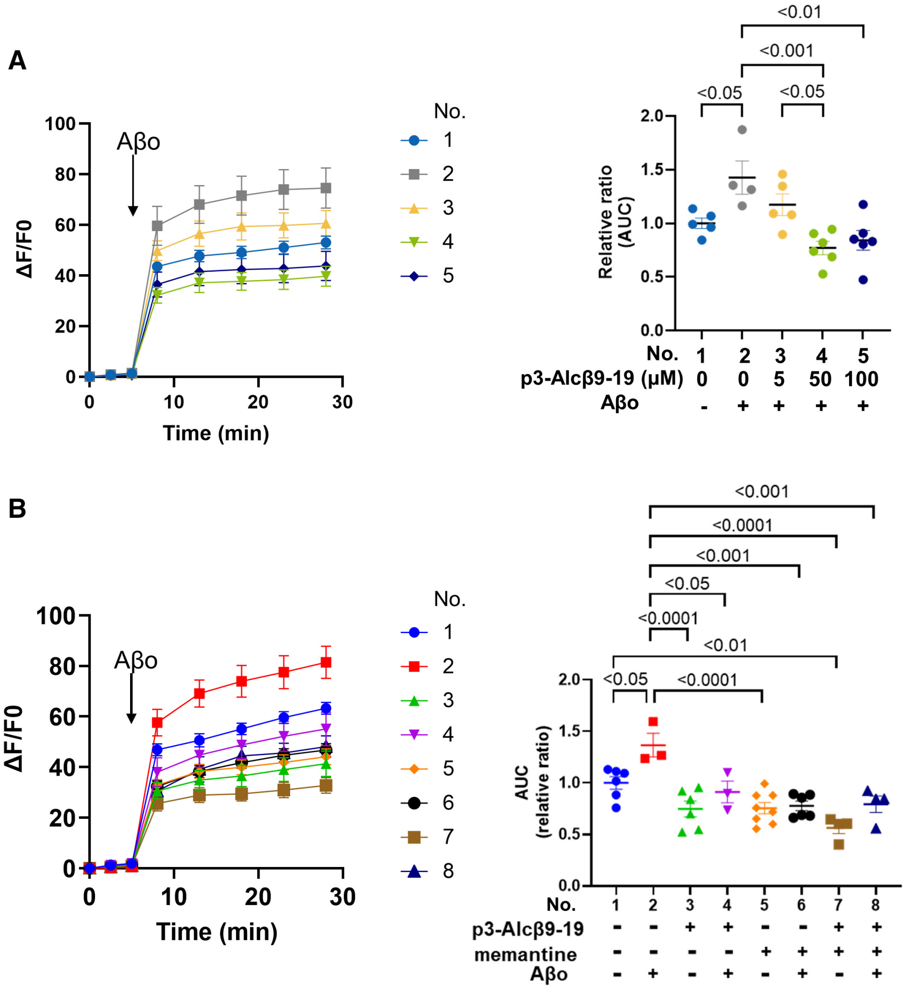

Figure EV2. p3-Alcβ9-19 dose-dependently suppresses Aβ42-triggered neuronal Ca^{2+} influx and nonsynergistic suppression of Ca^{2+} influx induced by Aβ42 oligomers (Aβo) in neurons by p3-Alcβ and memantine.

A Dose–response effect of p3-Alcβ9-19. Mouse neurons (div 15) pretreated with Fluo 4-AM were stimulated at 5 min (arrow) with (+) or without (–) Aβo (5.2 μM) in the presence of the stipulated amount of p3-Alcβ9-19 (μM). The fluorescence intensity was recorded at the indicated time (left) and the fluorescence area intensity for 23 min (5–28 min at the indicated time points) is shown (right) as the AUC expressed relative to that of cells cultured in the absence of p3-Alcβ and Aβo (assigned a value of 1.0 in No. 1). Statistical significance was determined with a one-way ANOVA with the Tukey's multiple comparison test (mean ± SEM; n = 4–6) and significant P-values ($P < 0.05$, $P < 0.01$, $P < 0.001$) are indicated on the graph.

B Effects of p3-Alcβ and memantine. Neurons (div 13) pretreated with Fluo 4-AM were stimulated with or without Aβo (5.2 μM) at 5 min (arrow) in the presence (+) or absence (–) of p3-Alcβ9-19 (50 μM) and memantine (5 μM). Fluorescence intensity was recorded at the indicated time (left), and the fluorescence area intensity for 23 min (5–28 min at the indicated time points) is shown (right) as the AUC expressed relative to that of cells cultured in experiment No. 1 (assigned a value of 1.0). Statistical significance was determined by a one-way ANOVA with the Tukey's multiple comparison test (mean ± SEM; n = 3–8), and significant P-values ($P < 0.05$, $P < 0.01$, $P < 0.001$, $P < 0.0001$) are indicated on the graph.

Data information: Experimental numbers indicate biological replicates. Detailed information including the statistical summary is described in Dataset EV2. Source data are available online for this figure.

 © 2023 The Authors

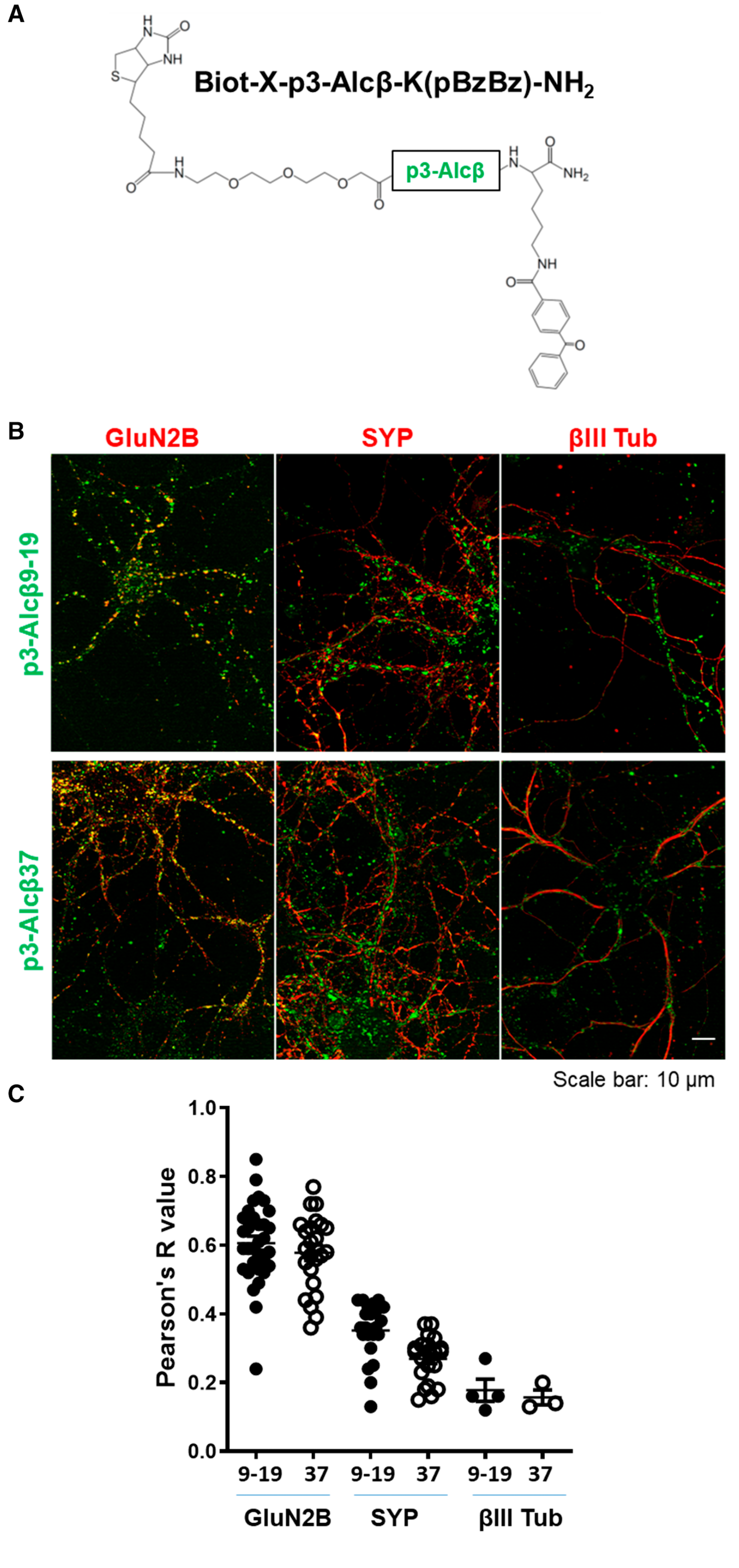

Figure EV3. Colocalization of p3-Alcβ associated to neurons with neuronal proteins.

A Structure of biotin-X-p3-Alcβ-K(pBzBz)-NH_2. Alcβ-KO mouse neurons (div 14) were incubated with biotin-X-p3-Alcβ9-19-K(pBzBz)-NH_2 or biotin-X-p3-Alcβ37-K(pBzBz)-NH_2 followed by UV irradiation.

B Localization of biotin-X-p3-Alcβ-K(pBzBz)-NH_2 probes associated with neurons. The cells were fixed and immunostained with antibodies against GluN2B, synaptophysin (SYP), and βIII-tubulin (βIII Tub). Localization of p3-Alcβ was visualized with streptavidin-Alexa488 (green), while the immunoreactive neuronal proteins were localized with an Alexa546-conjugated second antibody (red). Scale bar, 10 μm.

C Colocalization efficiency between p3-Alcβ and neuronal proteins. Colocalization was calculated using the coloc2 plug-in. The colocalization rates of proteins and p3-Alcβ were calculated from each frame of images (15,750 μm^2) of neurons and are indicated as Pearson's *R* value. Independent cell stainings were performed one to three times per cell preparation, and three to five frames were acquired from each well. All values were combined and subjected to statistical analysis with the indicated number of independent biological repeats (mean ± SEM). Pearson's coefficients are shown (*R* value of 1.0 indicates perfect colocalization while an *R* value of 0 indicates random localization).

Data information: Experimental numbers indicate frame numbers. Detailed information including the statistical summary is described in Dataset EV2. Source data are available online for this figure.

© 2023 The Authors

Figure EV4. Specific sELISA system for the detection of p3-Alcβ9-19 and transport of subcutaneously administered p3-Alcβ into blood and CSF, and coronal parametric PET images of [^{11}C]DPA713 SUVR in wild-type and AD mouse model following subcutaneous injection of p3-Alcβ9-19, and PET images of [^{18}F]BCPP-EF SUVR in monkeys following transdermal administration of vehicle and p3-Alcβ9-19.

A–C Sandwich ELISA for the quantification of p3-Alcβ9-19 and the determination of the pharmacokinetic profile of p3-Alcβ9-19. (A) Specific reactivity of the sELISA system with p3-Alcβ9-19. Indicated amounts of synthetic p3-Alcβ9-19 (closed circles), p3-Alcβ37 (closed squares), and the mixtures of the indicated amounts of p3-Alcβ9-19 with 1,000 pg/ml p3-Alcβ37 (open triangles) were dissolved in buffer A (PBS containing 1% bovine serum albumin and 0.05% Tween-20) and assayed in duplicate by sELISA. Immunoreactivities were detected as described in "Materials and Methods." Results are reported as mean ± SEM (n = 2). (B) Transport of p3-Alcβ9-19 into the blood. p3-Alcβ9-19 (1 mg/kg of body weight, n = 4 per time point) was administered subcutaneously to wild-type (WT) mice (9-month-old). Blood was collected from their tail veins at the indicated time points, the plasma was diluted 200-fold, and p3-Alcβ9-19 concentrations were quantified by sELISA. Results are reported as mean ± SEM (C) Transport of p3-Alcβ9-19 into CSF. P3-Alcβ9-19 (5 mg/kg body weight, n = 4 per time point) was administered subcutaneously to WT mice (5-month-old). CSF was collected from four mice, respectively, at the indicated time points and diluted 20-fold, and p3-Alcβ9-19 concentrations were quantified by sELISA. Results are reported as mean ± SEM.

D–F PET imaging with [^{11}C]DPA713. (D) The SUVRs of [^{11}C]DPA713 in the cortex and hippocampus of wild-type (WT) and AD mice (APP-KI) with (APP-KI/p3-Alcβ9-19) or without (APP-KI) subcutaneous injection of p3-Alcβ9-19 (1 mg/kg body weight). The PET data are superimposed on X-ray CT images, and the color bar denotes the SUVR. (E, F) The SUVRs of [^{18}F]BCPP-EF in the cortex (E) and hippocampus (F) are compared. Statistical analysis was performed using a one-way ANOVA, followed by Bonferroni correction for multiple comparisons (mean ± SEM; n = 5–7), and the significant P-value ($P < 0.01$) is indicated on the graph.

G–I Increased mitochondrial activity after administration of p3-Alcβ9-19 to monkeys. Three consecutive PET scans in the same *Rhesus* monkeys (n = 2) were performed after the transdermal administration of vehicle and p3-Alcβ9-19 (0.5 mg/kg and 1 mg/kg). The PET images of [^{18}F]BCPP-EF SUVR were displayed in parallel with magnetic resonance (MR) images (G), and the color bar denotes the SUVR (H). The bar graph indicates the percentile increase in binding (I). Results are reported as mean ± SEM (n = 2).

Data information: Experimental numbers indicate biological replicates with two monkeys. Detailed information including the statistical summary is described in Dataset EV2.
Source data are available online for this figure.

 © 2023 The Authors

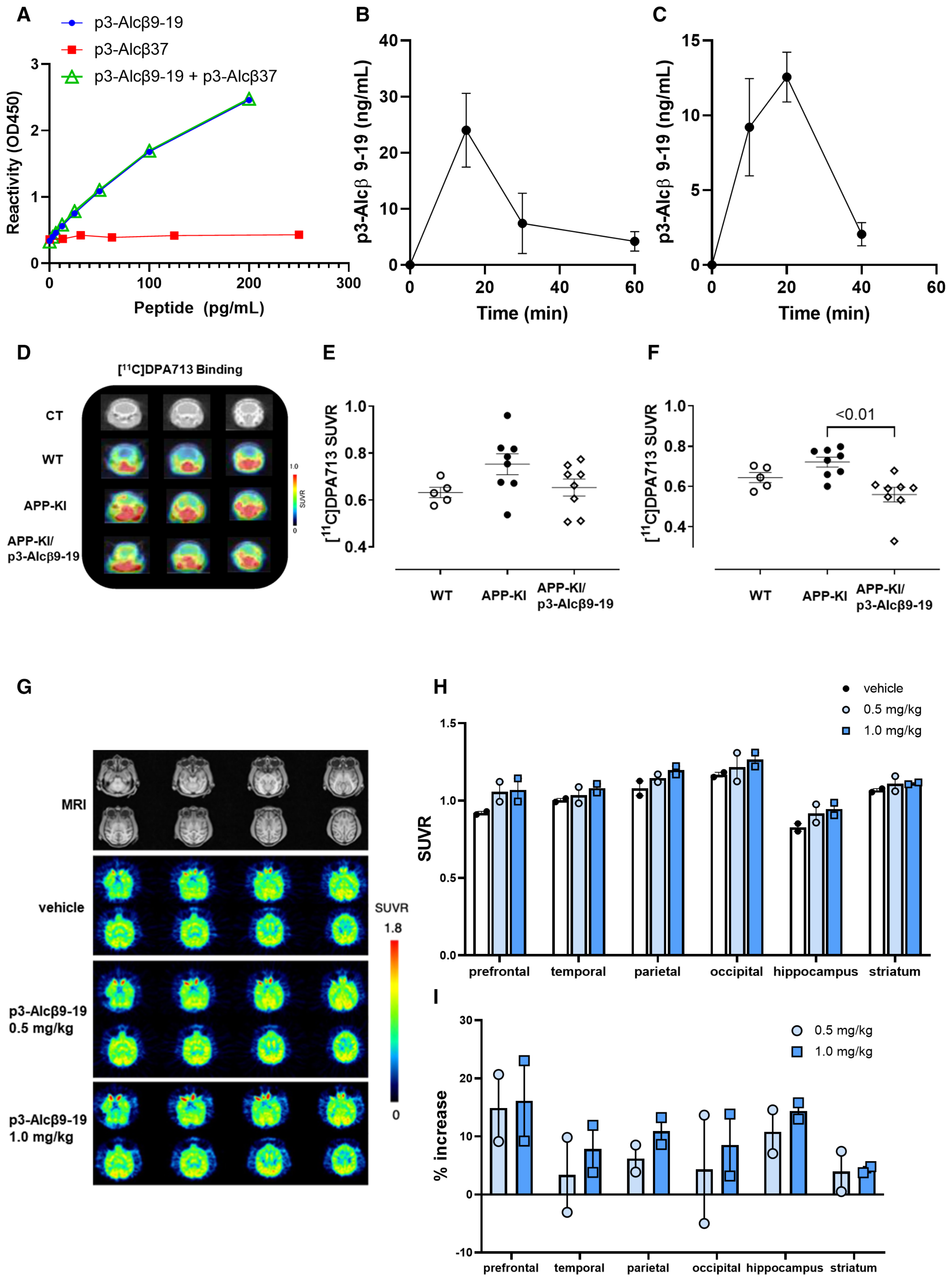
A
p3-Alcβ9-19
p3-Alcβ37
p3-Alcβ9-19 + p3-Alcβ37
Reactivity (OD450)
Peptide (pg/mL)
B
p3-Alcβ 9-19 (ng/mL)
Time (min)
C
p3-Alcβ 9-19 (ng/mL)
Time (min)
D
[11C]DPA713 Binding
CT
WT
APP-KI
APP-KI/
p3-Alcβ9-19
SUVR
E
[11C]DPA713 SUVR
WT
APP-KI
APP-KI/
p3-Alcβ9-19
F
[11C]DPA713 SUVR
<0.01
WT
APP-KI
APP-KI/
p3-Alcβ9-19
G
MRI
vehicle
p3-Alcβ9-19
0.5 mg/kg
p3-Alcβ9-19
1.0 mg/kg
SUVR
1.8
0
H
vehicle
0.5 mg/kg
1.0 mg/kg
SUVR
prefrontal temporal parietal occipital hippocampus striatum
I
0.5 mg/kg
1.0 mg/kg
% increase
prefrontal temporal parietal occipital hippocampus striatum

Figure EV4.

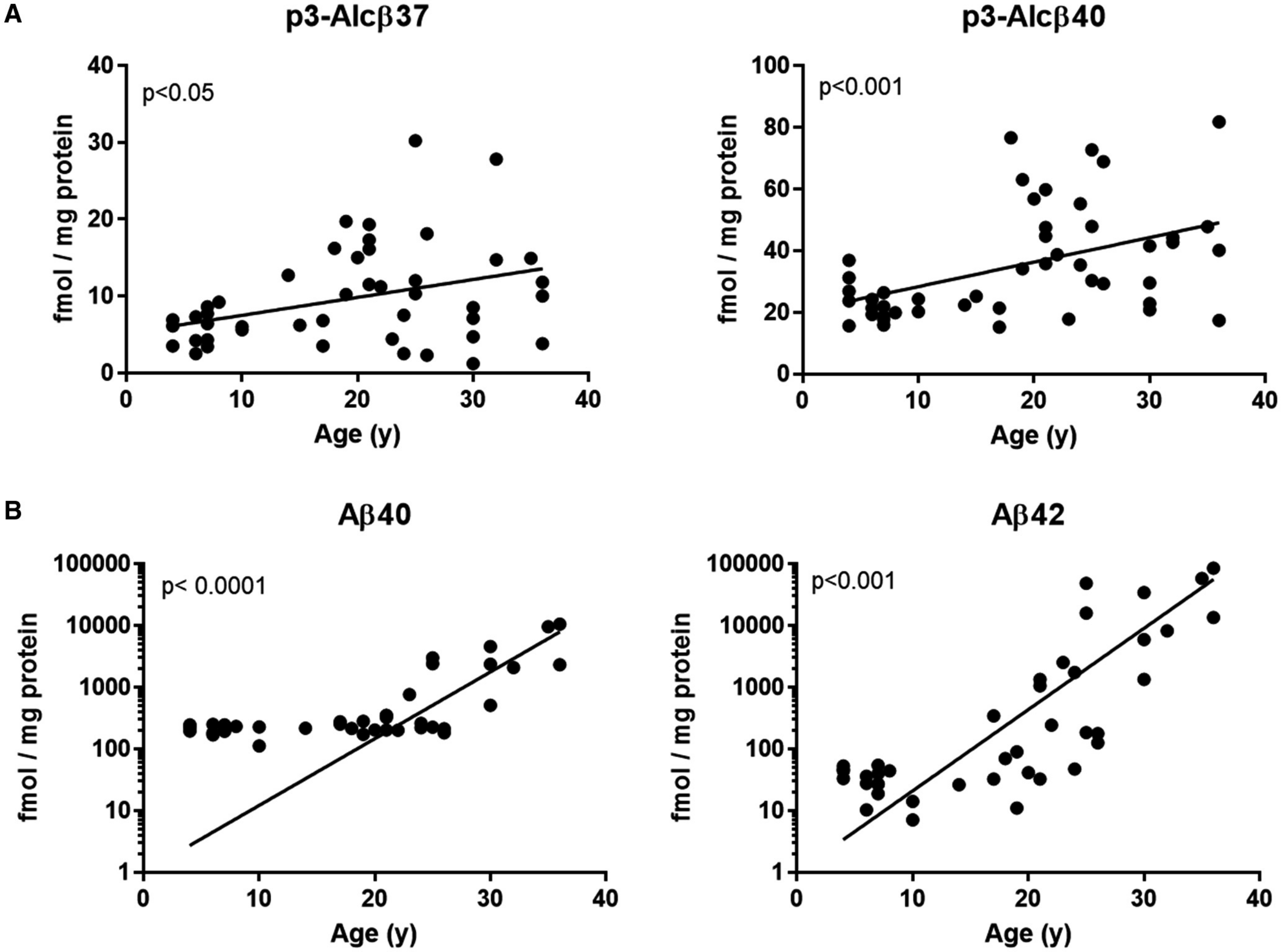

Figure EV5. Age-dependent changes of p3-Alcβ and Aβ in monkey brain parenchyma.

A, B Temporal cortex tissue from *Cynomolgus* monkeys of various ages (y, years old, $n = 47$) was used. Changes in p3-Alcβ37 and p3-Alcβ40 levels (A), and Aβ40 and Aβ42 levels (B) in the TBS-insoluble fraction are shown as a function of age. Statistical analysis was performed using Pearson's correction (A left, $r^2 = 0.1379$; A right $r^2 = 0.216$; B left, $r^2 = 0.3347$; B right, $r^2 = 0.2873$); P-values < 0.05 were considered statistically significant.

Data information: Experimental numbers indicate biological replicates. Detailed information including the statistical summary is described in Dataset EV2.
Source data are available online for this figure.

 © 2023 The Authors

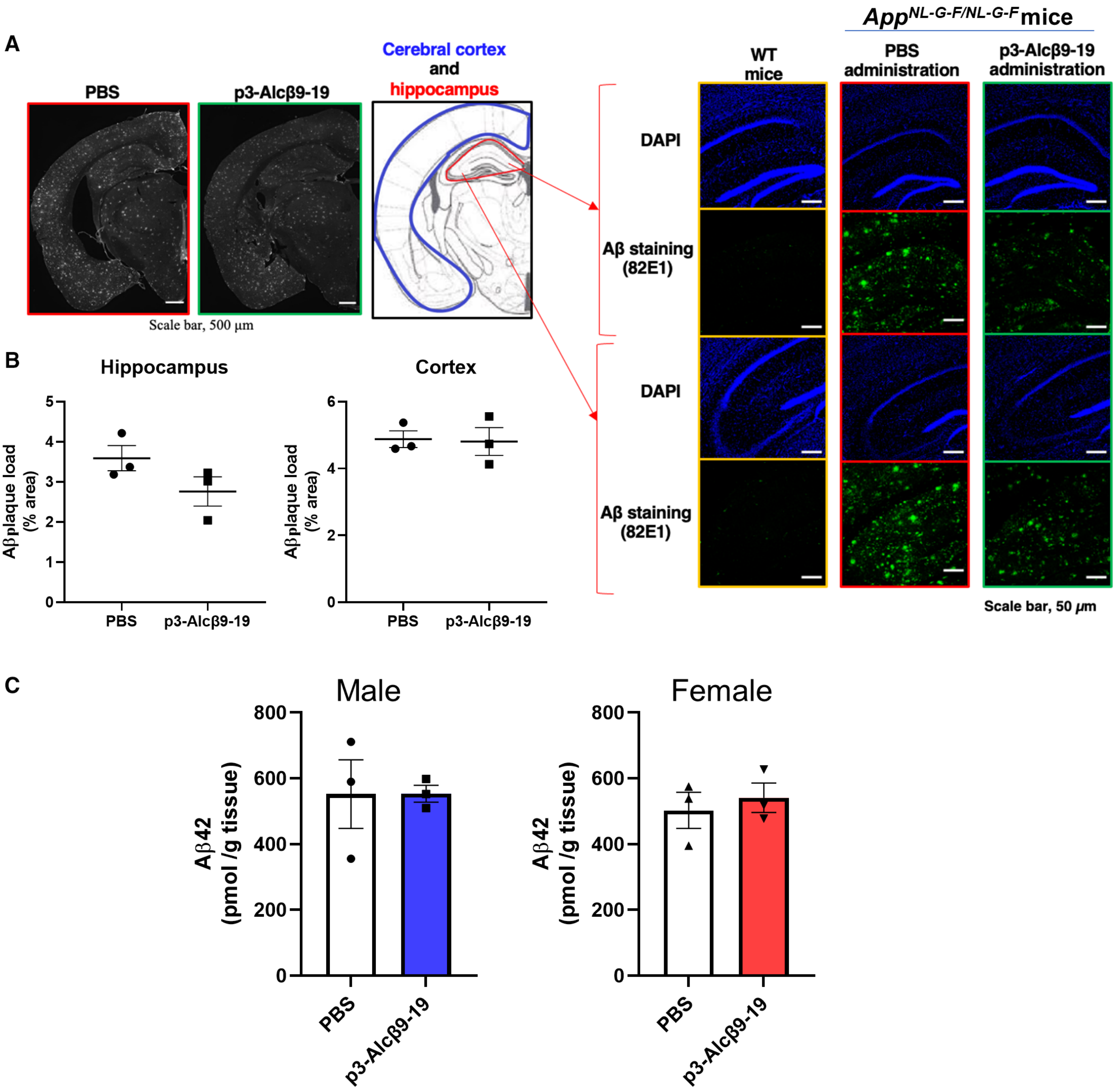

Figure EV6. Aβ accumulation and Aβ42 levels in the cerebral cortex and hippocampus in a mouse model of AD with or without subcutaneous administration of p3-Alcβ9-19.

A *App*$^{NL-G-F/NL-G-F}$ mice (9-month-old females) were subcutaneously administered p3-Alcβ9-19 (1 mg/kg body weight) or PBS daily for 30 days. The brain sections (20-µm-thick) were immunostained with anti-human Aβ antibody (green) along with DAPI staining (blue). Magnified views of the hippocampus of *APP*$^{NL-G-F}$ mice are shown in the panels on the right together with the corresponding area in wild-type (WT) mice.

B Aβ plaque-occupied areas within the regions bordered by blue (cerebral cortex) and red (hippocampus) lines in the hemisphere of the brain (panel A) were quantified by NIH Image J software. After adjusting for the threshold within the indicated area, the percent area above the threshold was measured to obtain the total area that was occupied by Aβ (as displayed in percentage). Slices from three mice with p3-Alcβ9-19 or PBS administration were quantified (n = 3, one slice per mouse) and statistically analyzed with the unpaired Student's t-test (mean ± SEM, n = 3).

C *App*$^{NL-G-F/NL-G-F}$ mice (9-month-old males and females) were subcutaneously administered p3-Alcβ9-19 (1 mg/kg body weight) or PBS daily for 30 days as described above. The cerebral cortex and hippocampus were dissected and then homogenized. Human Aβ42 levels in the lysates were quantified and the amounts of Aβ42 were compared between AD mice that were or were not administered p3-Alcβ9-19. Statistical significance was determined by the unpaired Student's t-test (mean ± SEM, n = 3 [3 male (left) or female (right) mice per experimental group]).

Data information: Experimental numbers indicate biological replicates. Detailed information including the statistical summary is described in Dataset EV2.
Source data are available online for this figure.

© 2023 The Authors
