# Supplementary figures and images for: Brain p3‐Alcβ peptide restores neuronal viability impaired by Alzheimer's amyloid β‐peptide
Source: EMBO Mol Med. 2023 Mar 30;15(5):e17052. doi: 10.15252/emmm.202217052 (PMC10165357; doi:10.15252/emmm.202217052)

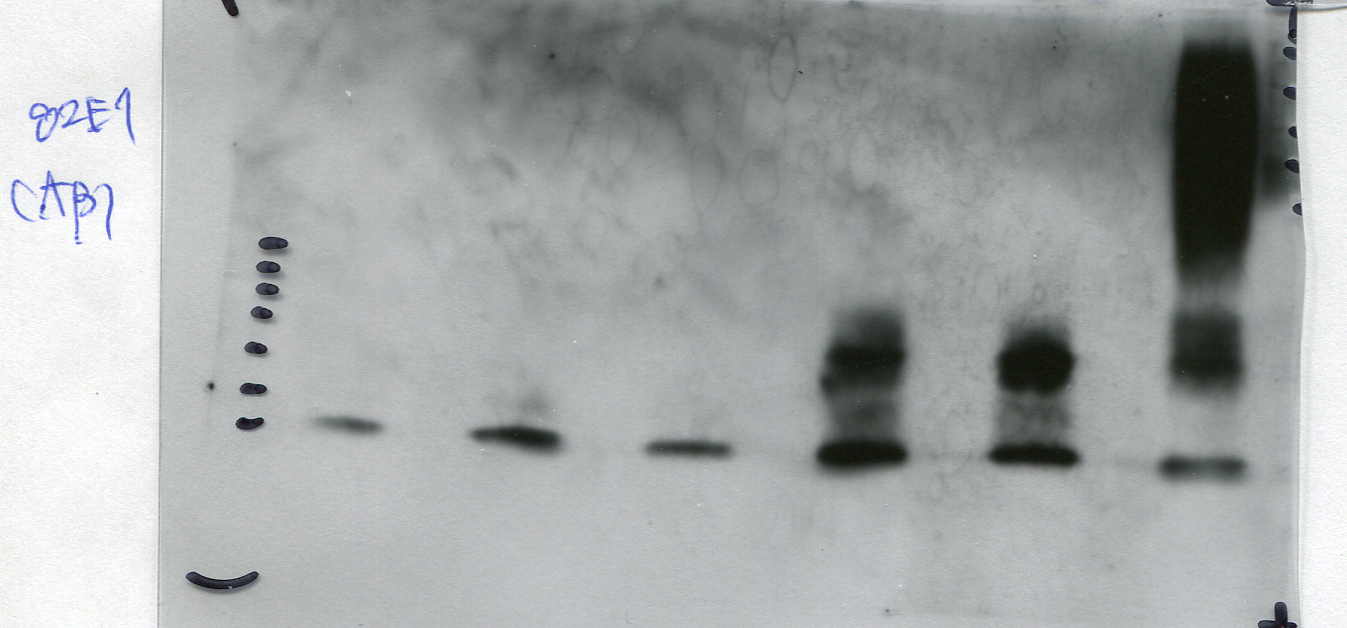

Supplement: Supplementary file 5 — Source Data for Expanded View [file EMMM-15-e17052-s012.zip › EV source data/Fig EV1/FigEV1B/Aâ└.tif]

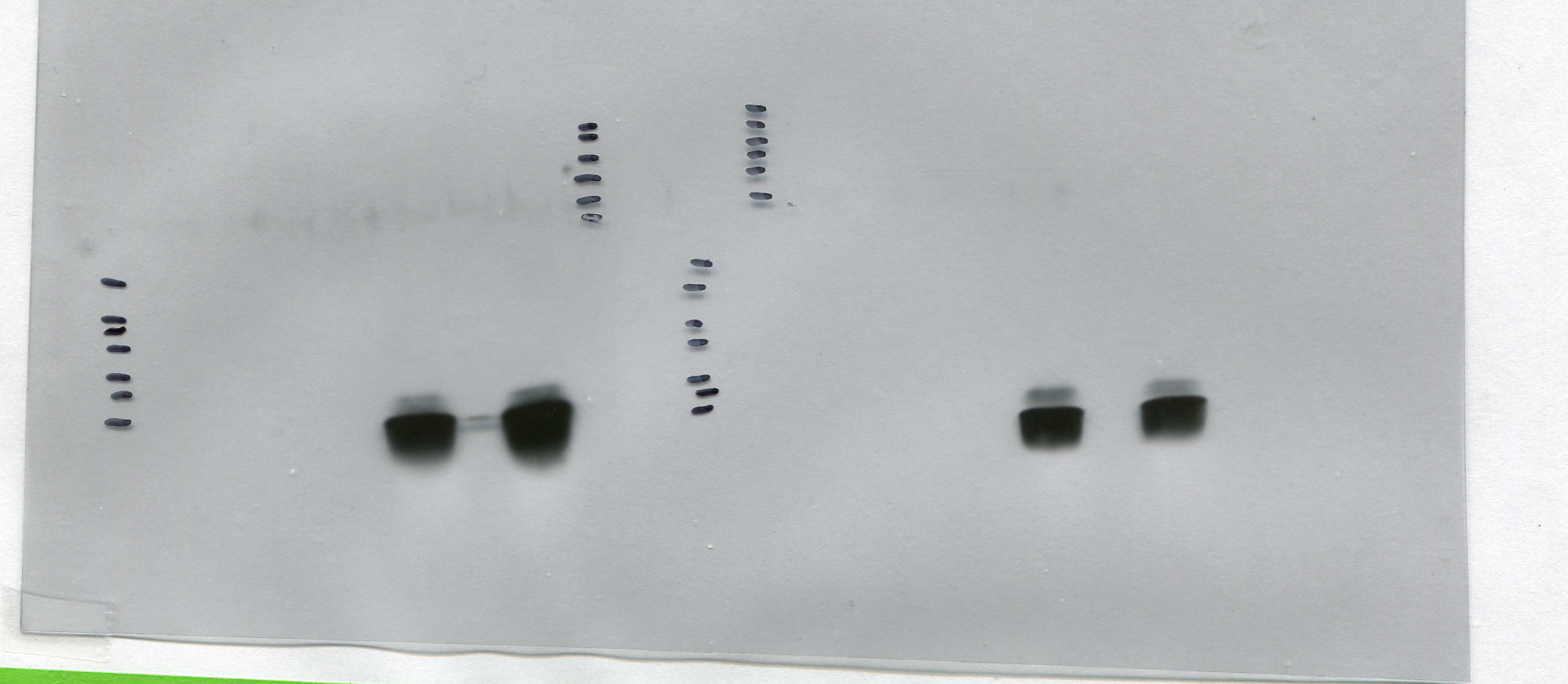

Supplement: Supplementary file 5 — Source Data for Expanded View [file EMMM-15-e17052-s012.zip › EV source data/Fig EV1/FigEV1B/p3-Alc .tif]

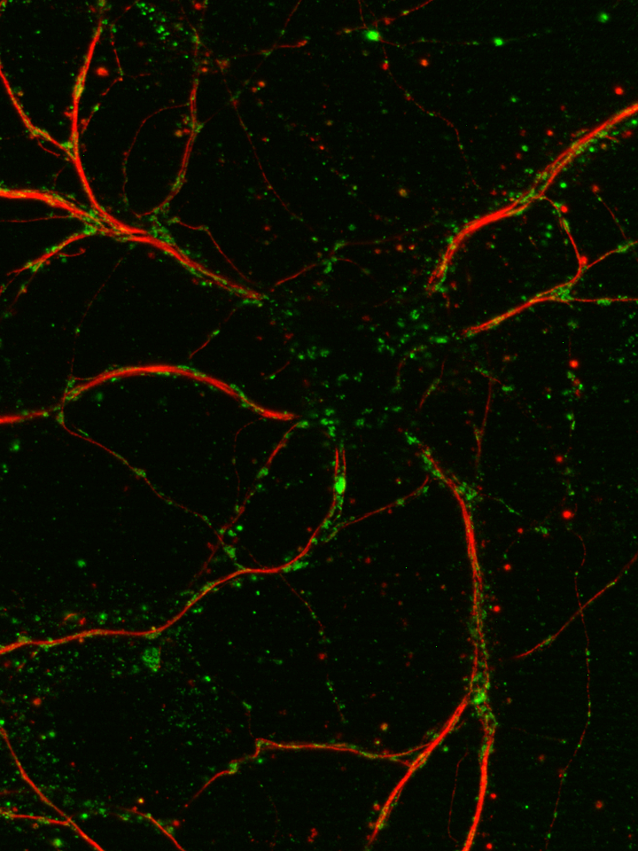

Supplement: Supplementary file 5 — Source Data for Expanded View [file EMMM-15-e17052-s012.zip › EV source data/Fig EV3/FigEV3B/bIIITub;1-37.tif]

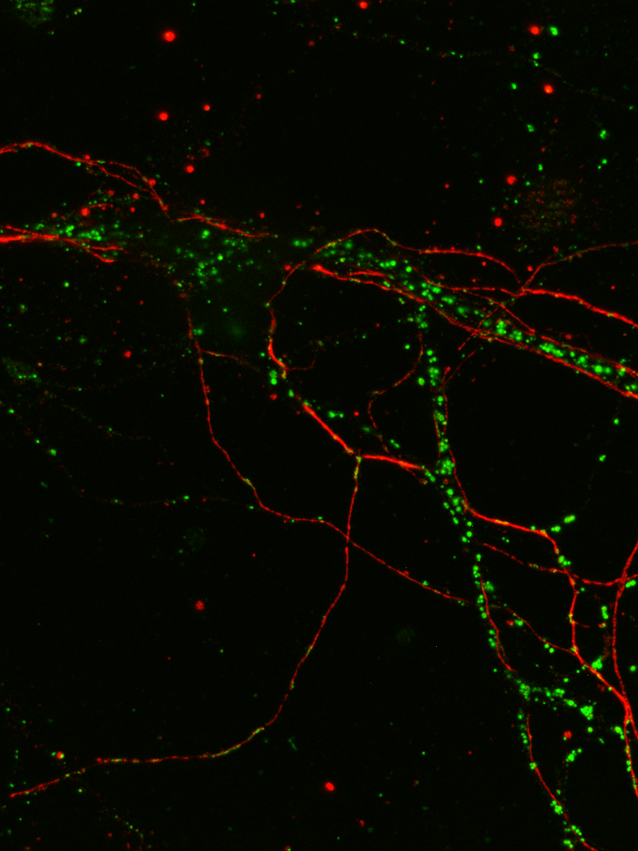

Supplement: Supplementary file 5 — Source Data for Expanded View [file EMMM-15-e17052-s012.zip › EV source data/Fig EV3/FigEV3B/bIIITub;9-19.tif]

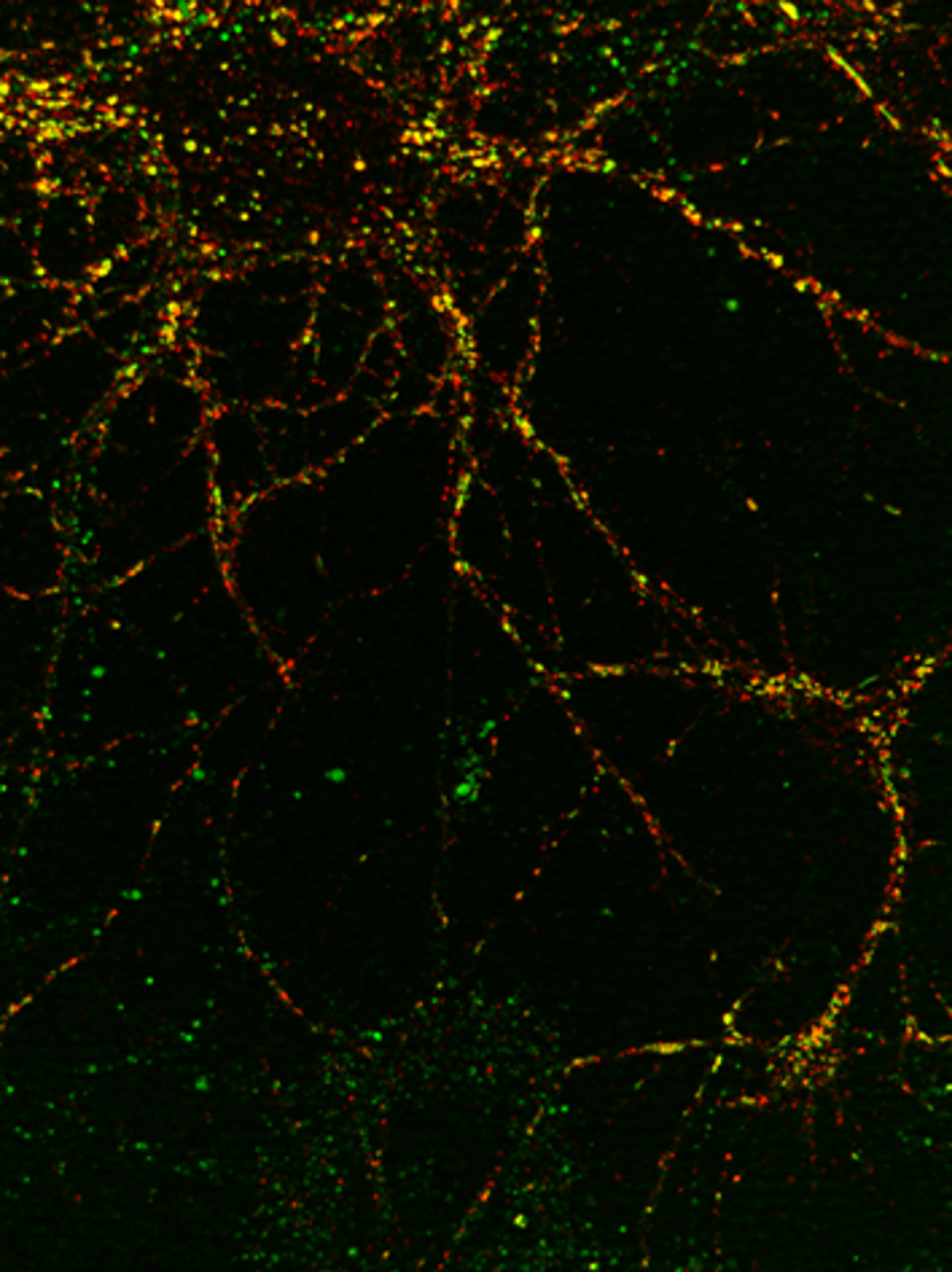

Supplement: Supplementary file 5 — Source Data for Expanded View [file EMMM-15-e17052-s012.zip › EV source data/Fig EV3/FigEV3B/GluN2B;1-37.tif]

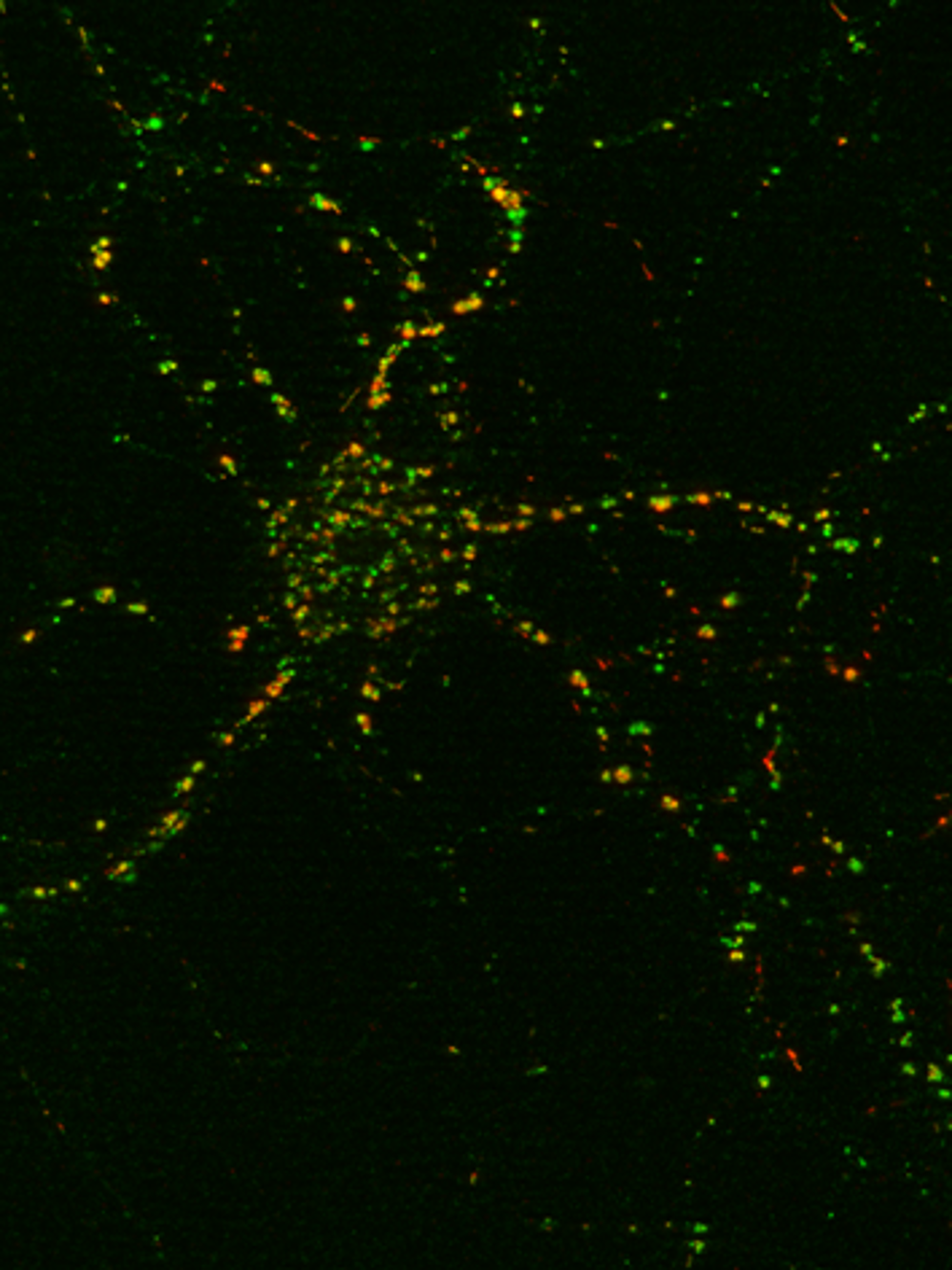

Supplement: Supplementary file 5 — Source Data for Expanded View [file EMMM-15-e17052-s012.zip › EV source data/Fig EV3/FigEV3B/GluN2B;9-19.tif]

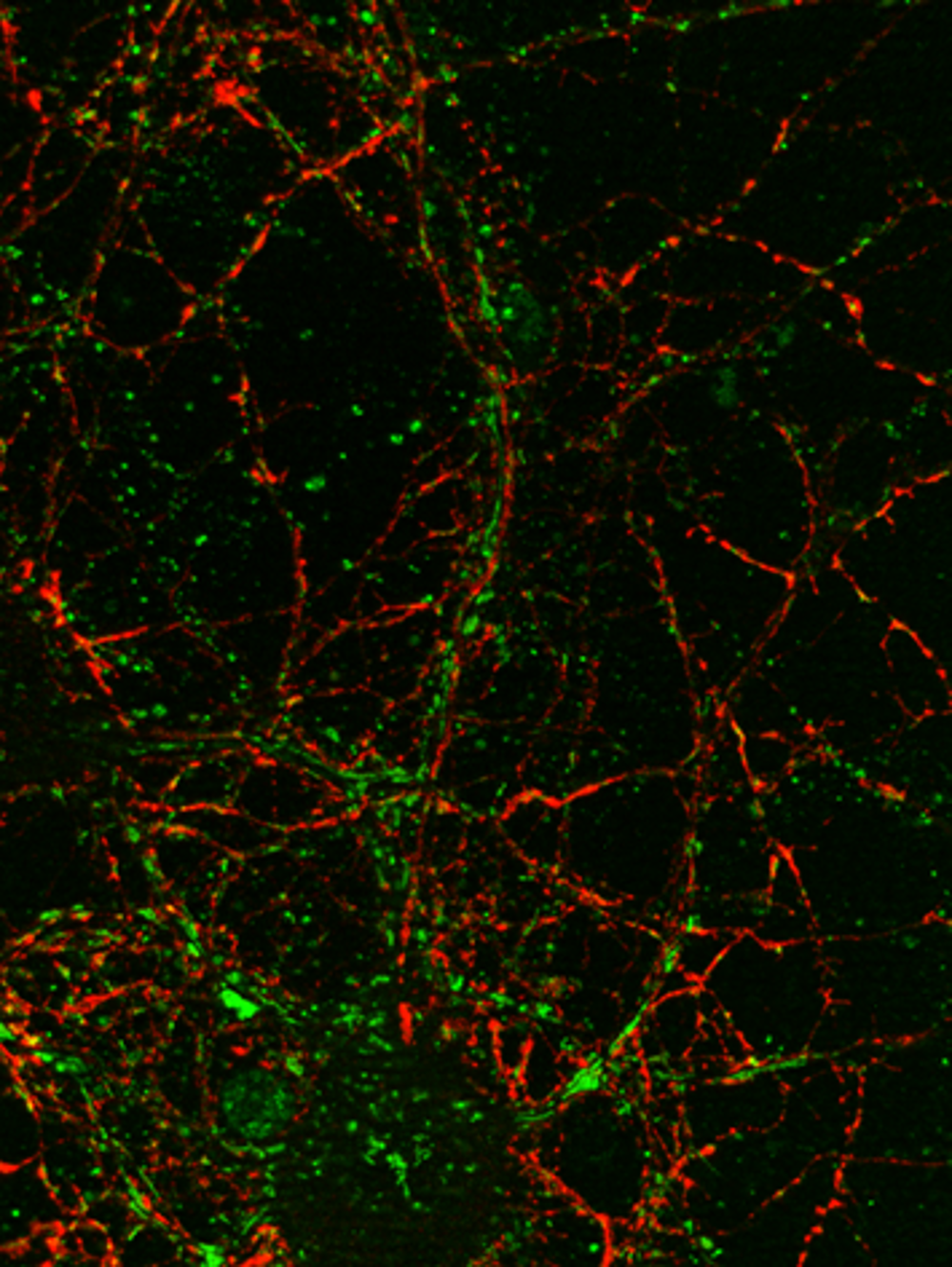

Supplement: Supplementary file 5 — Source Data for Expanded View [file EMMM-15-e17052-s012.zip › EV source data/Fig EV3/FigEV3B/SYP;1-37.tif]

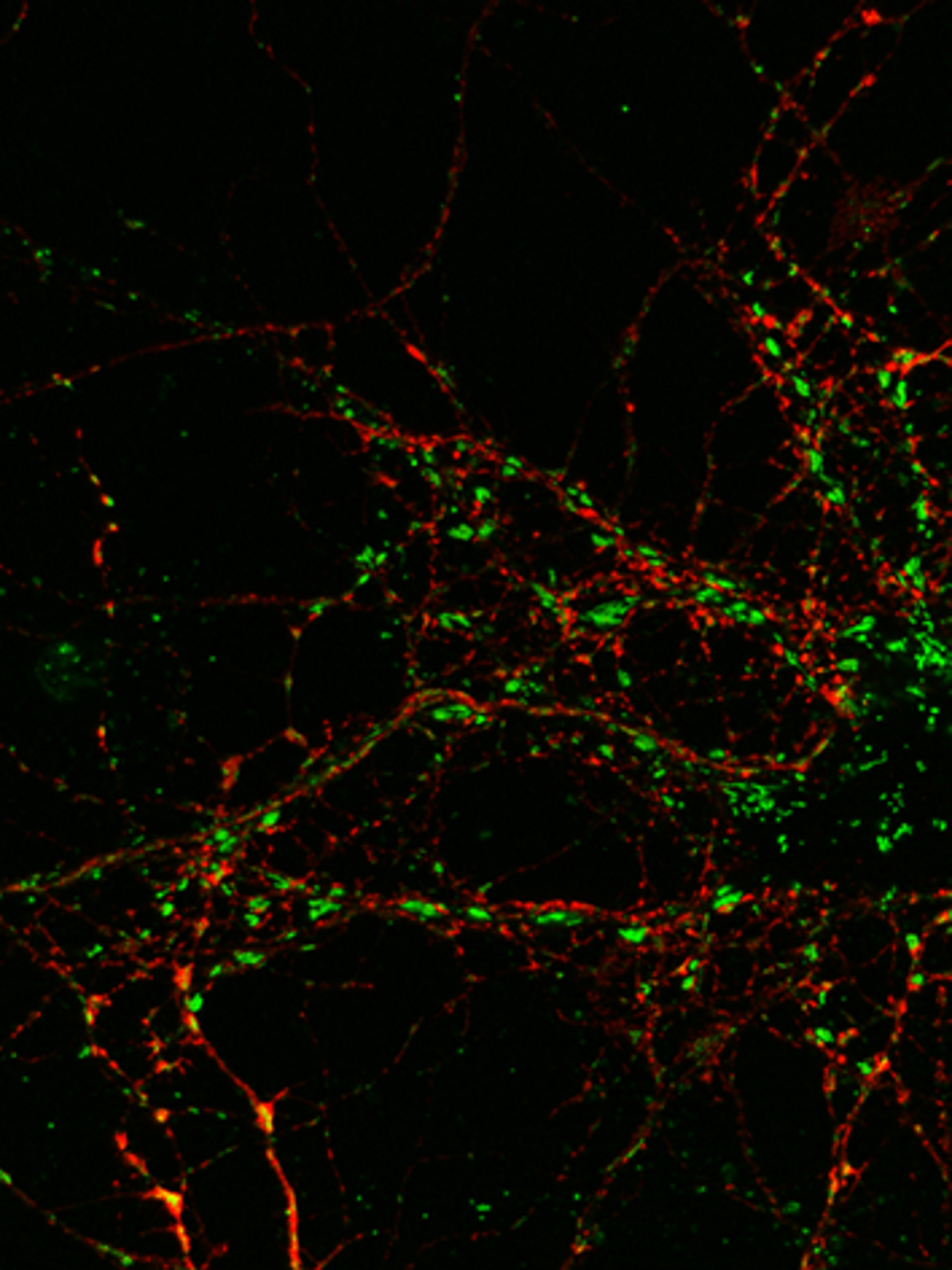

Supplement: Supplementary file 5 — Source Data for Expanded View [file EMMM-15-e17052-s012.zip › EV source data/Fig EV3/FigEV3B/SYP;9-19.tif]

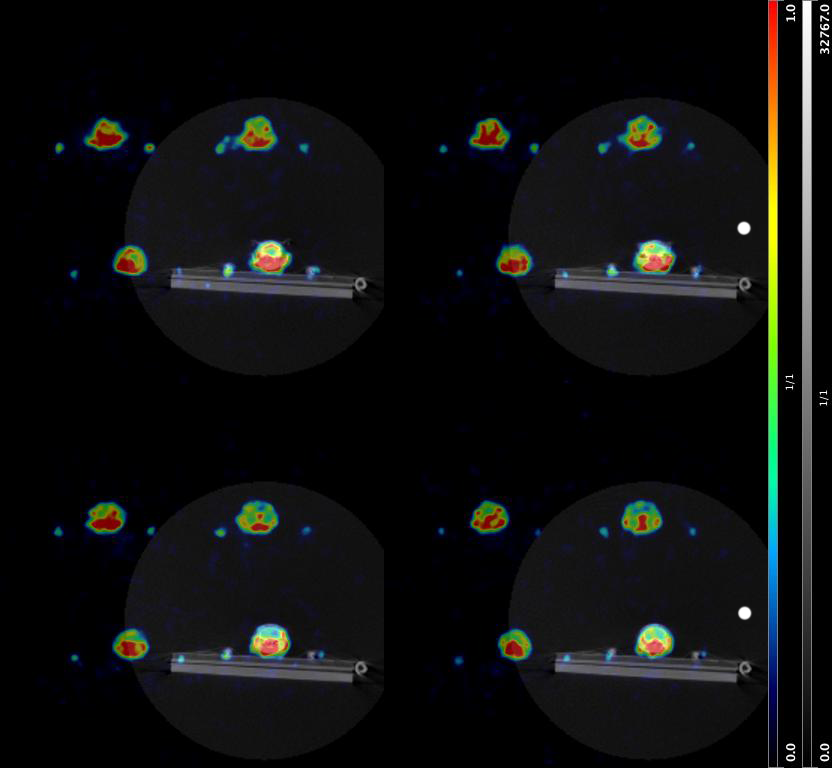

Supplement: Supplementary file 5 — Source Data for Expanded View [file EMMM-15-e17052-s012.zip › EV source data/Fig EV4/FigEV4D/DPA_APP_1.tif]

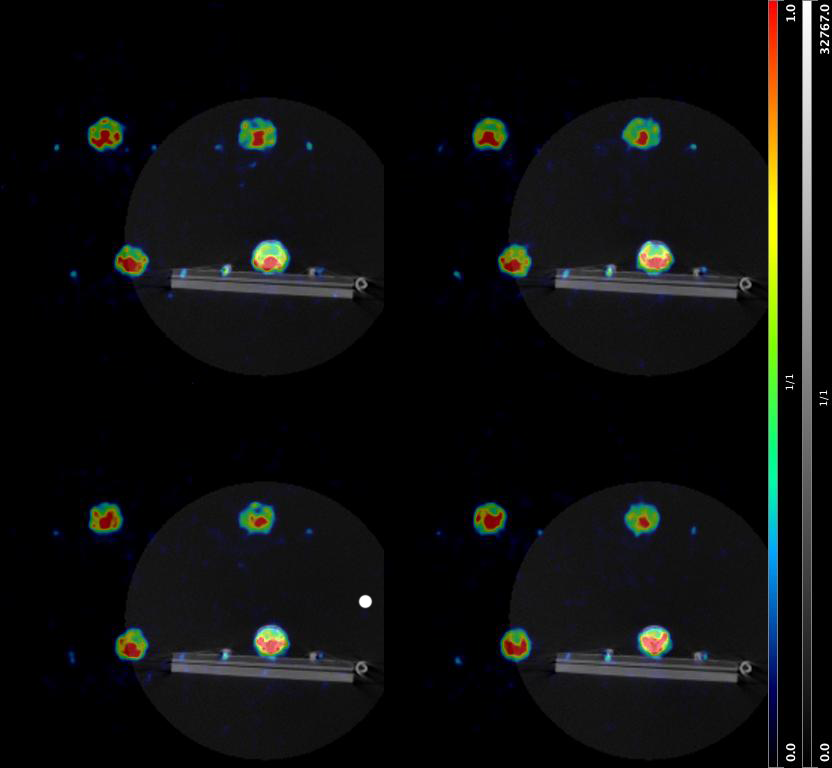

Supplement: Supplementary file 5 — Source Data for Expanded View [file EMMM-15-e17052-s012.zip › EV source data/Fig EV4/FigEV4D/DPA_APP_2.tif]

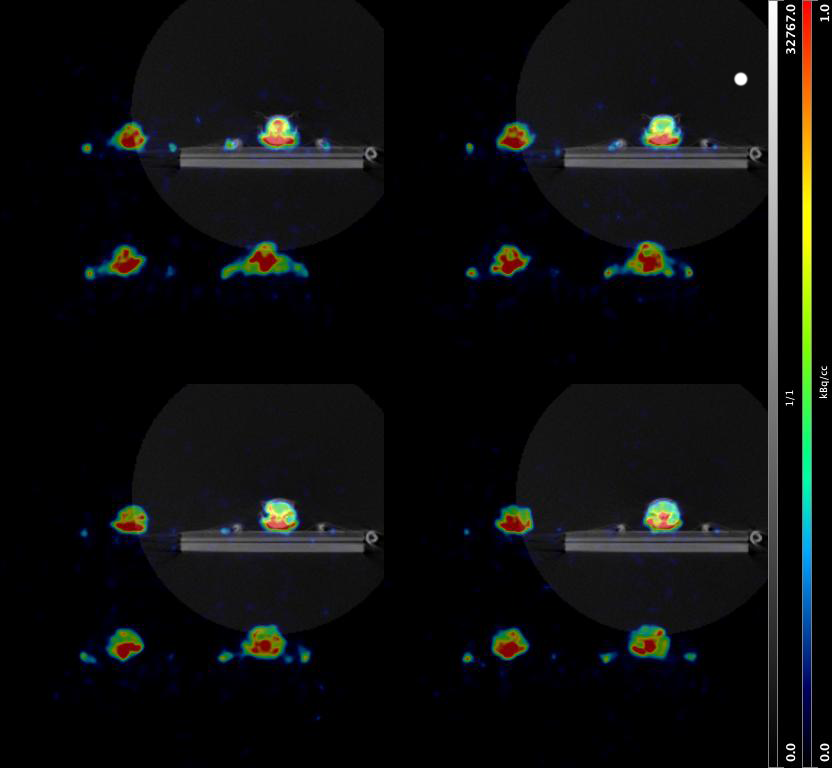

Supplement: Supplementary file 5 — Source Data for Expanded View [file EMMM-15-e17052-s012.zip › EV source data/Fig EV4/FigEV4D/DPA_APP-p3Alc_1.tif]

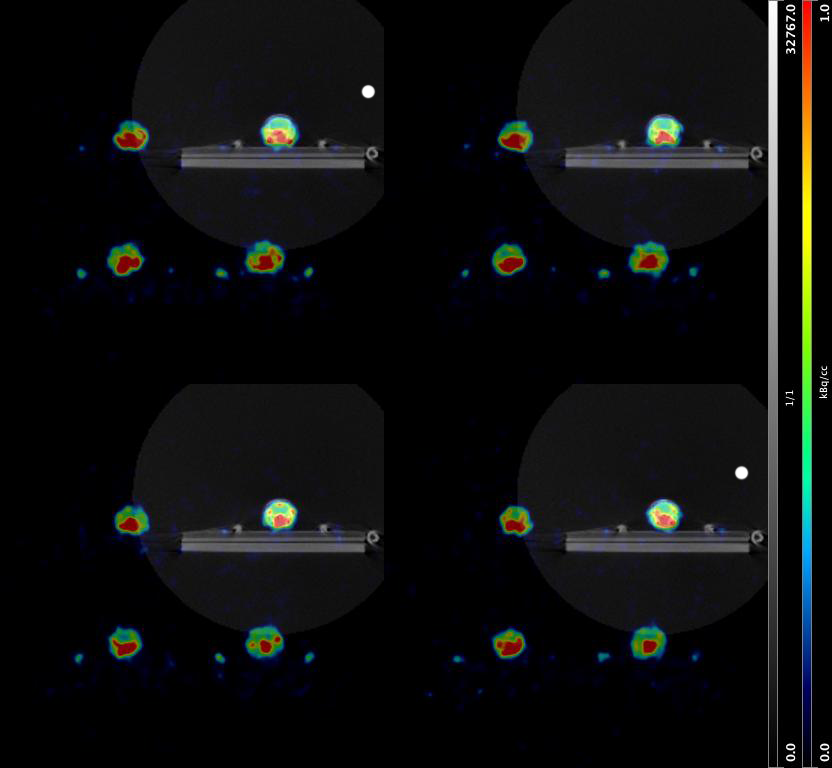

Supplement: Supplementary file 5 — Source Data for Expanded View [file EMMM-15-e17052-s012.zip › EV source data/Fig EV4/FigEV4D/DPA_APP-p3Alc_2.tif]

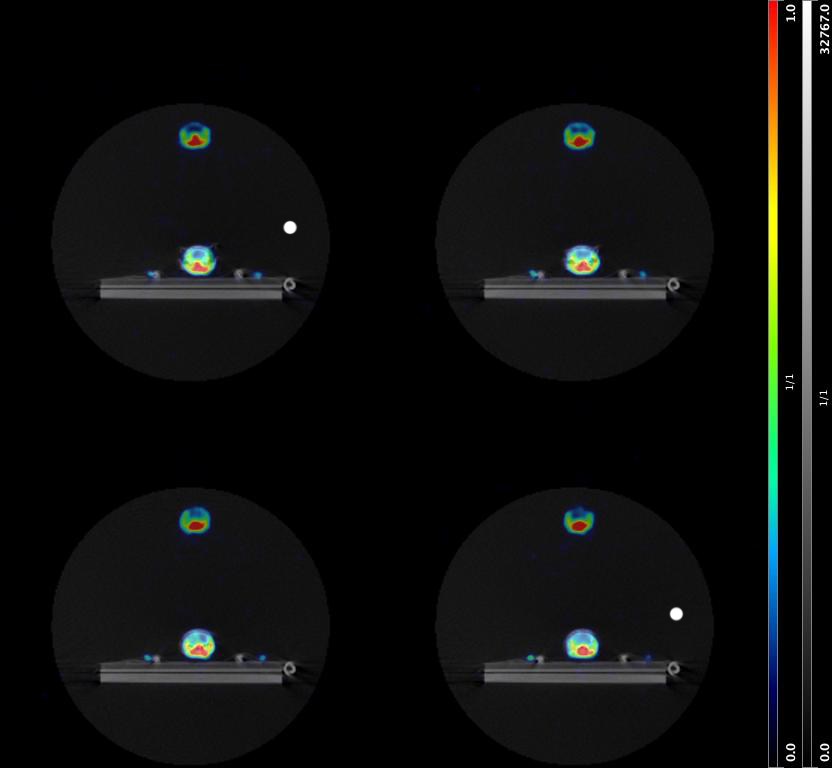

Supplement: Supplementary file 5 — Source Data for Expanded View [file EMMM-15-e17052-s012.zip › EV source data/Fig EV4/FigEV4D/DPA_WT_1.tif]

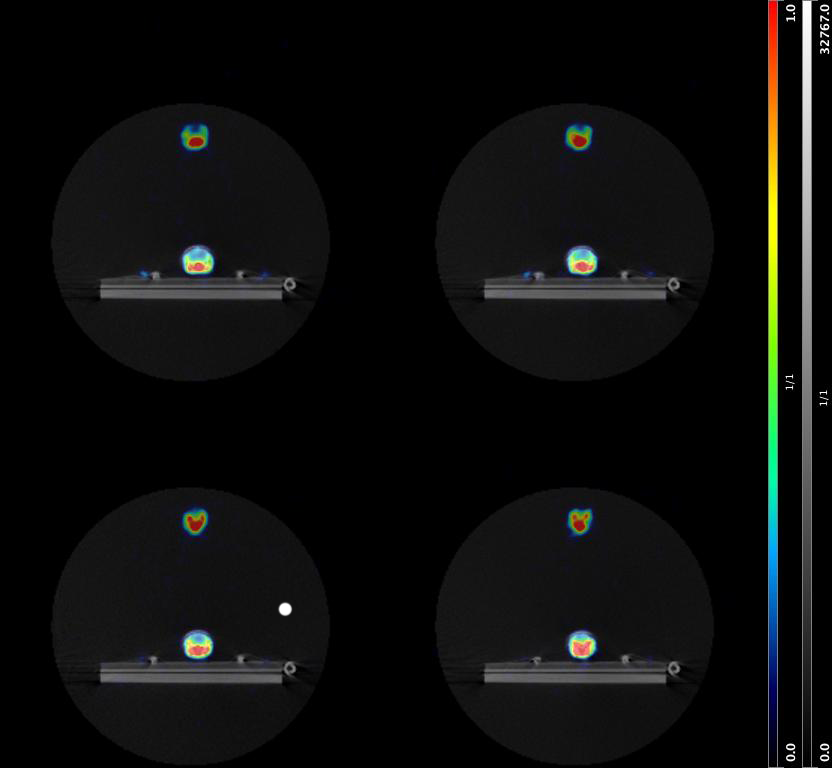

Supplement: Supplementary file 5 — Source Data for Expanded View [file EMMM-15-e17052-s012.zip › EV source data/Fig EV4/FigEV4D/DPA_WT_2.tif]

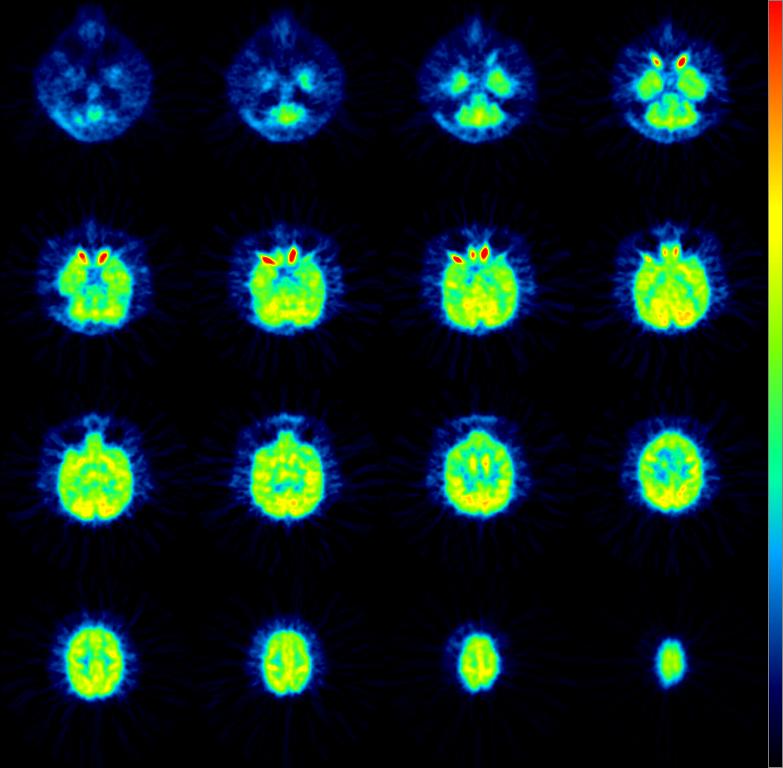

Supplement: Supplementary file 5 — Source Data for Expanded View [file EMMM-15-e17052-s012.zip › EV source data/Fig EV4/FigEV4G/Unpublished obs_7_0.5mgkg.jpg]

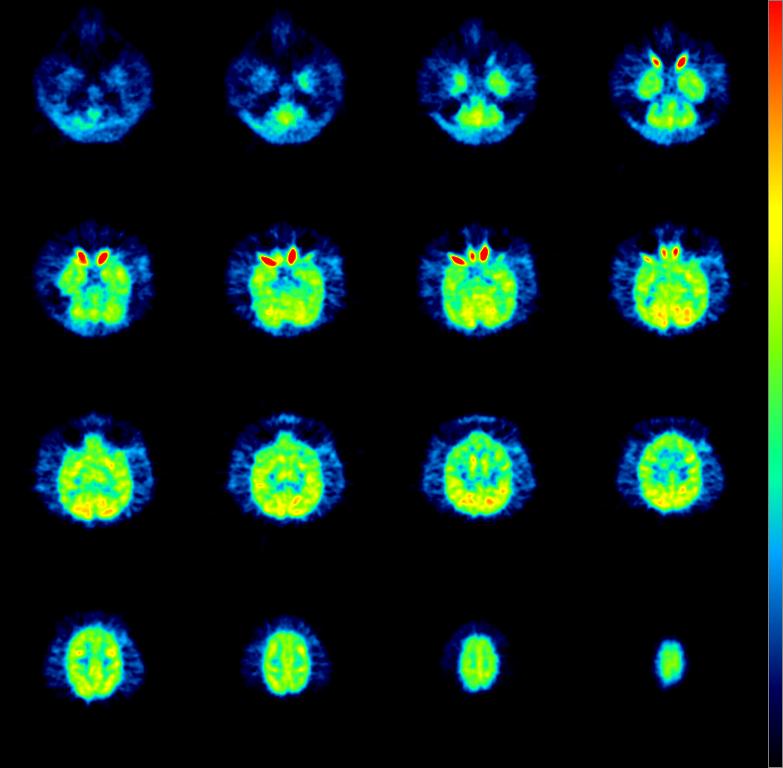

Supplement: Supplementary file 5 — Source Data for Expanded View [file EMMM-15-e17052-s012.zip › EV source data/Fig EV4/FigEV4G/Unpublished obs_7_1mgkg.jpg]

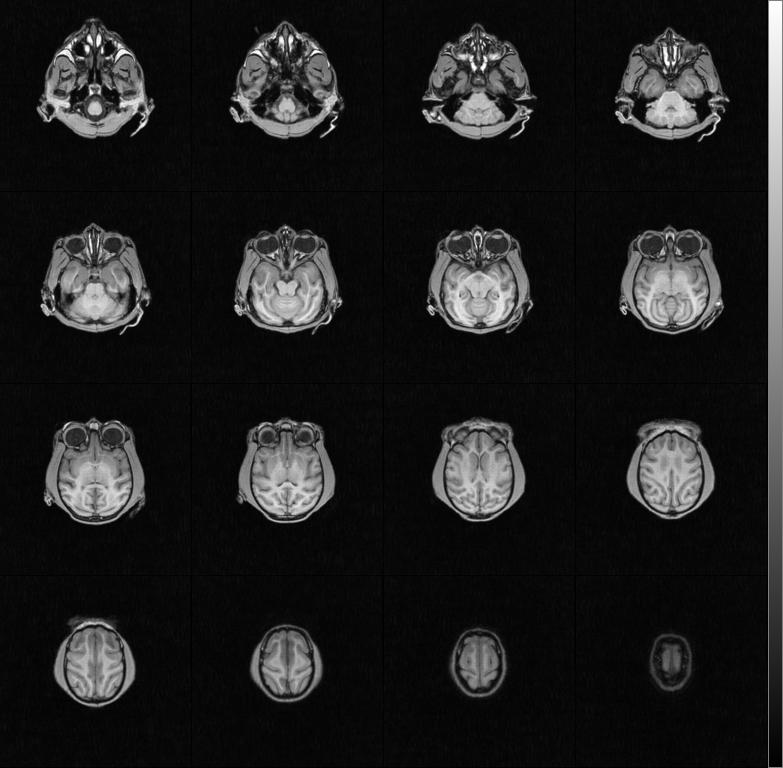

Supplement: Supplementary file 5 — Source Data for Expanded View [file EMMM-15-e17052-s012.zip › EV source data/Fig EV4/FigEV4G/Unpublished obs_7_MRI.jpg]

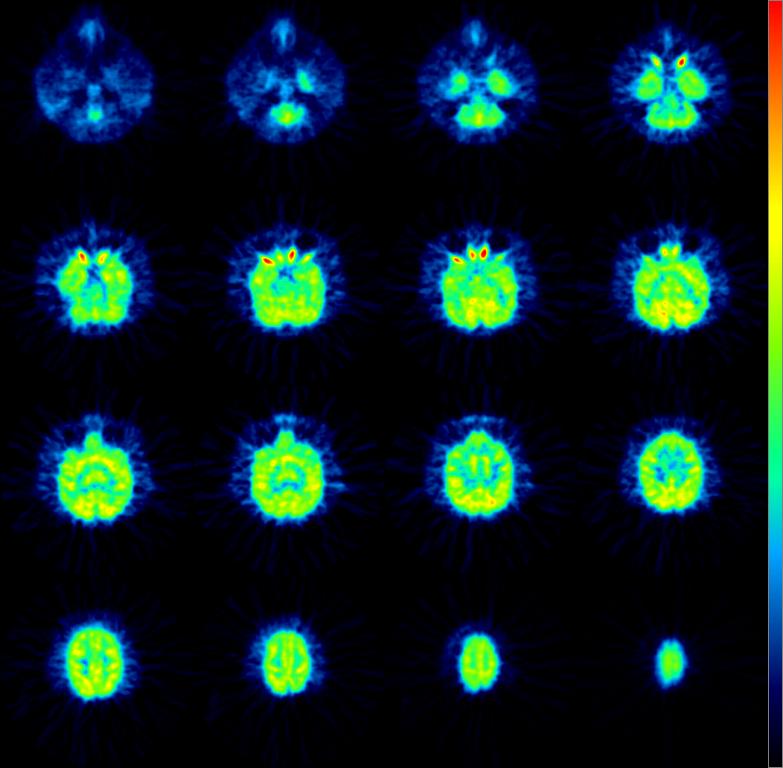

Supplement: Supplementary file 5 — Source Data for Expanded View [file EMMM-15-e17052-s012.zip › EV source data/Fig EV4/FigEV4G/Unpublished obs_7_vehicle.jpg]

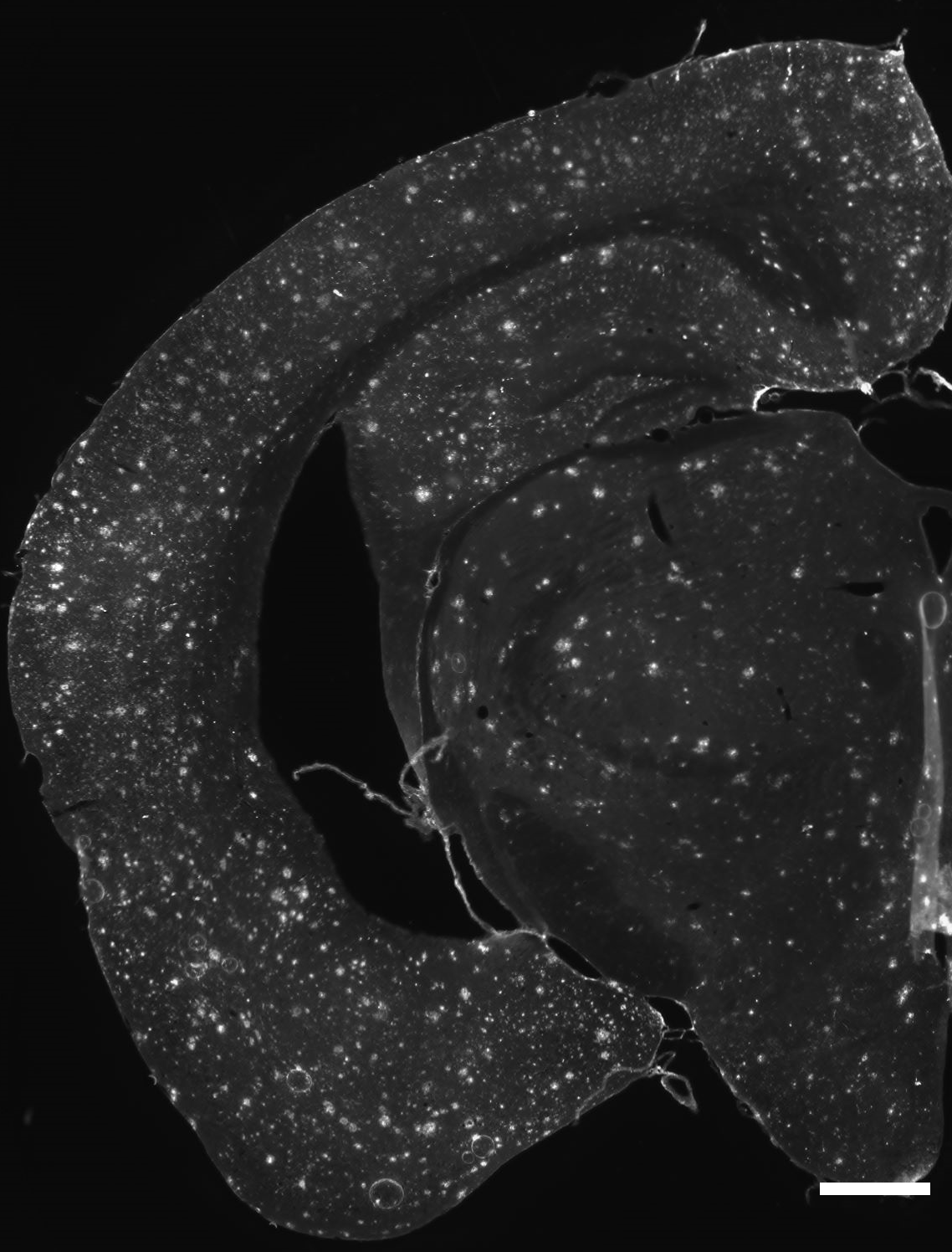

Supplement: Supplementary file 5 — Source Data for Expanded View [file EMMM-15-e17052-s012.zip › EV source data/Fig EV6/FigEV6A/01_PBS.tif]

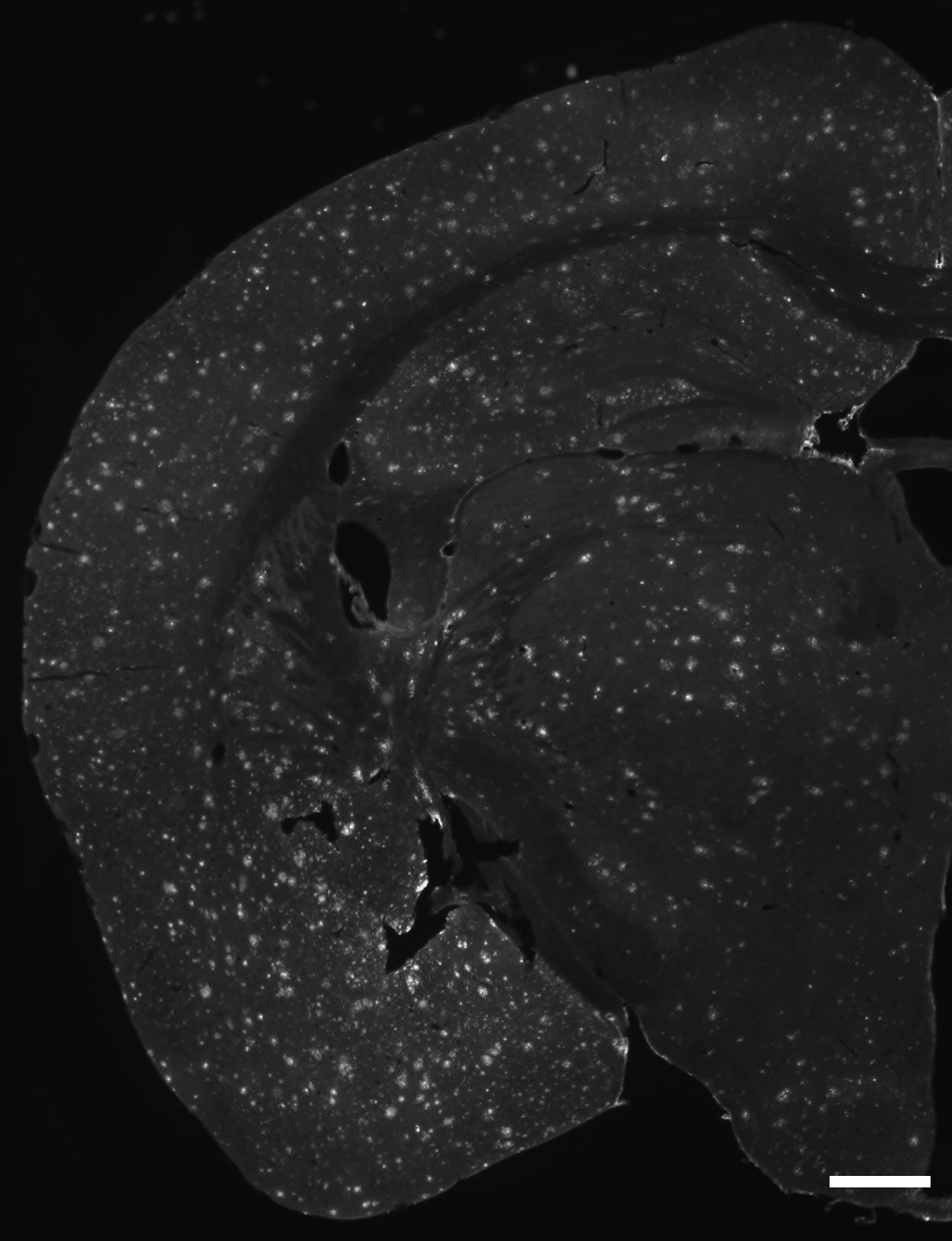

Supplement: Supplementary file 5 — Source Data for Expanded View [file EMMM-15-e17052-s012.zip › EV source data/Fig EV6/FigEV6A/02_p3-Alcb.tif]

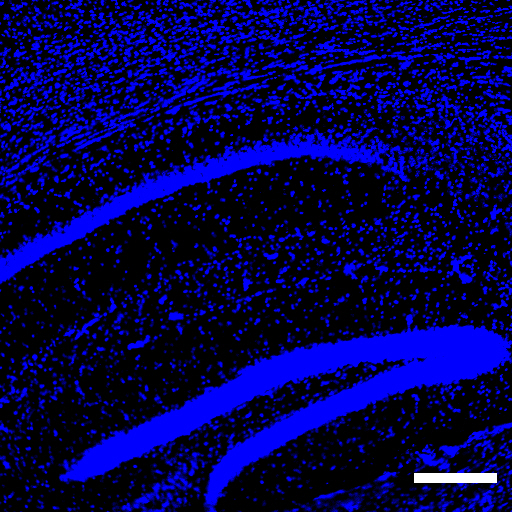

Supplement: Supplementary file 5 — Source Data for Expanded View [file EMMM-15-e17052-s012.zip › EV source data/Fig EV6/FigEV6A/03_WT_CA1_DAPI.tif]

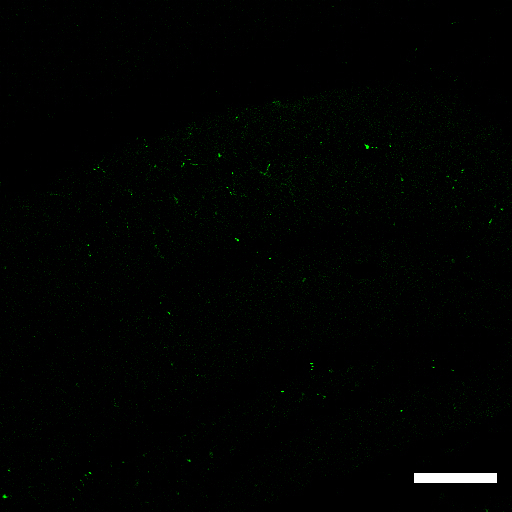

Supplement: Supplementary file 5 — Source Data for Expanded View [file EMMM-15-e17052-s012.zip › EV source data/Fig EV6/FigEV6A/04_WT_CA1_82E1.tif]

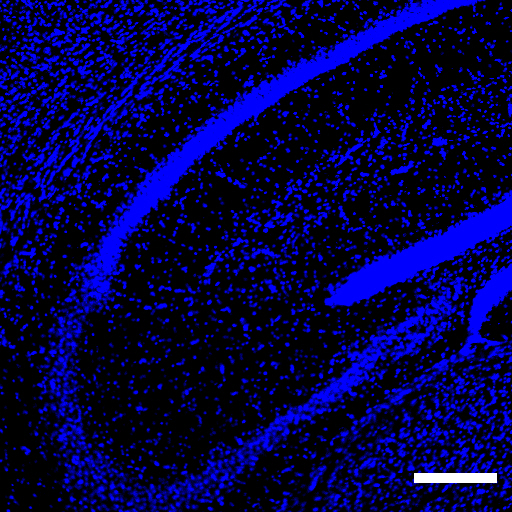

Supplement: Supplementary file 5 — Source Data for Expanded View [file EMMM-15-e17052-s012.zip › EV source data/Fig EV6/FigEV6A/05_WT_CA3_DAPI.tif]

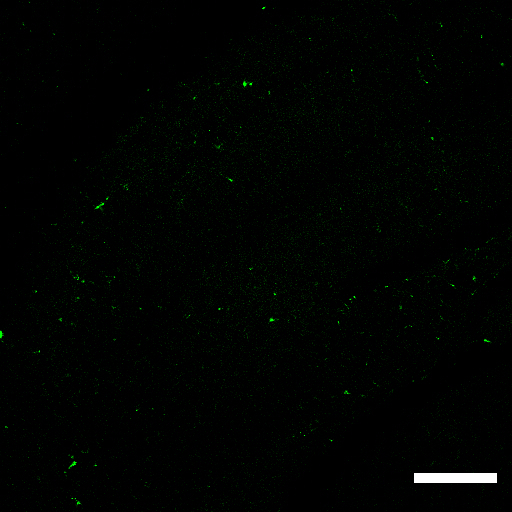

Supplement: Supplementary file 5 — Source Data for Expanded View [file EMMM-15-e17052-s012.zip › EV source data/Fig EV6/FigEV6A/06_WT_CA3_82E1.tif]

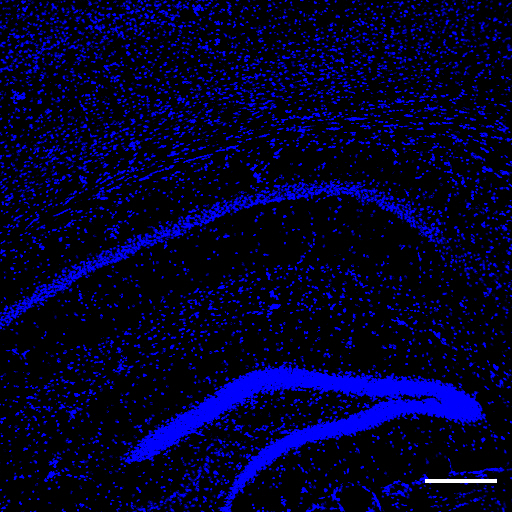

Supplement: Supplementary file 5 — Source Data for Expanded View [file EMMM-15-e17052-s012.zip › EV source data/Fig EV6/FigEV6A/07_PBS_CA1_DAPI.tif]

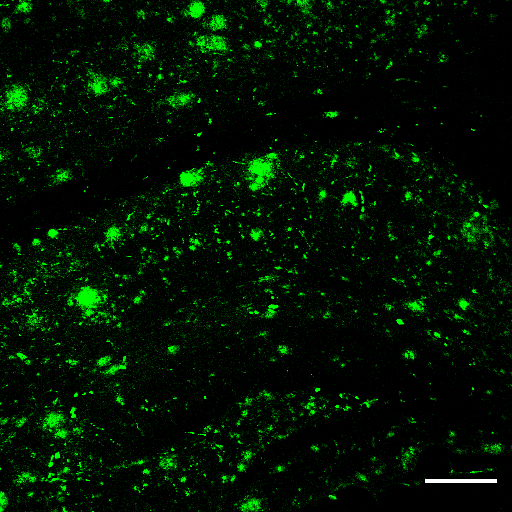

Supplement: Supplementary file 5 — Source Data for Expanded View [file EMMM-15-e17052-s012.zip › EV source data/Fig EV6/FigEV6A/08_PBS_CA1_82E1.tif]

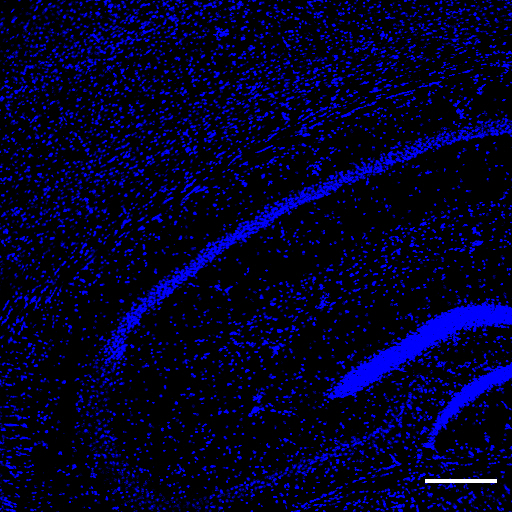

Supplement: Supplementary file 5 — Source Data for Expanded View [file EMMM-15-e17052-s012.zip › EV source data/Fig EV6/FigEV6A/09_PBS_CA3_DAPI.tif]

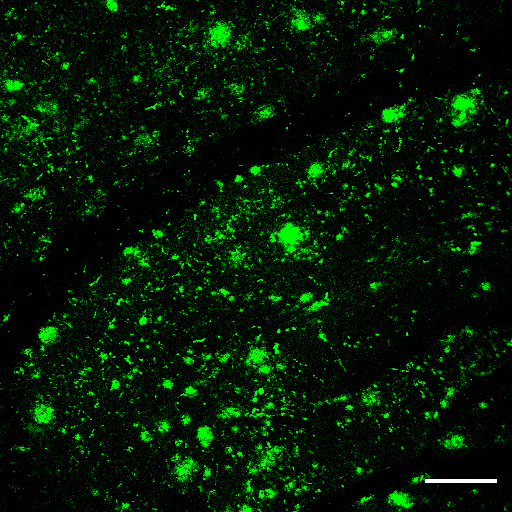

Supplement: Supplementary file 5 — Source Data for Expanded View [file EMMM-15-e17052-s012.zip › EV source data/Fig EV6/FigEV6A/10__PBS_CA3_82E1.tif]

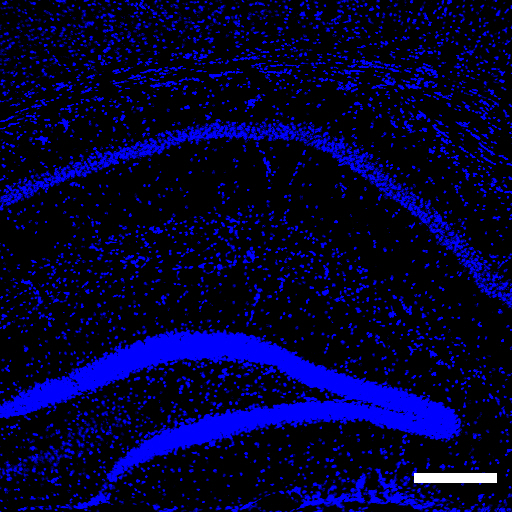

Supplement: Supplementary file 5 — Source Data for Expanded View [file EMMM-15-e17052-s012.zip › EV source data/Fig EV6/FigEV6A/11_p3-Alcb_CA1_DAPI.tif]

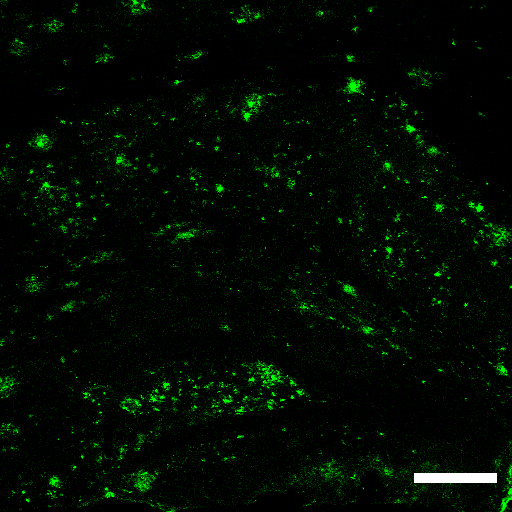

Supplement: Supplementary file 5 — Source Data for Expanded View [file EMMM-15-e17052-s012.zip › EV source data/Fig EV6/FigEV6A/12_p3-Alcb_CA1_82E1.tif]

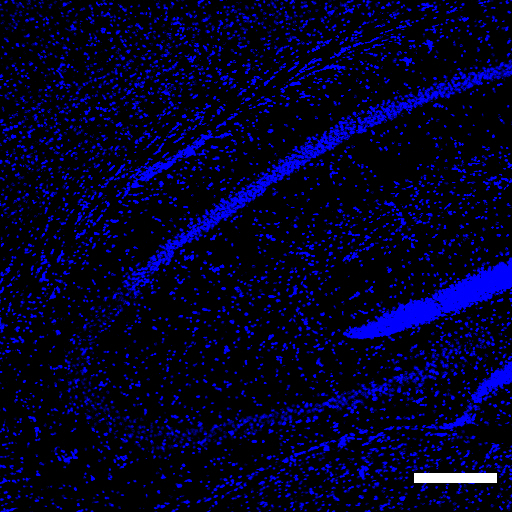

Supplement: Supplementary file 5 — Source Data for Expanded View [file EMMM-15-e17052-s012.zip › EV source data/Fig EV6/FigEV6A/13_p3-Alcb_CA3_DAPI.tif]

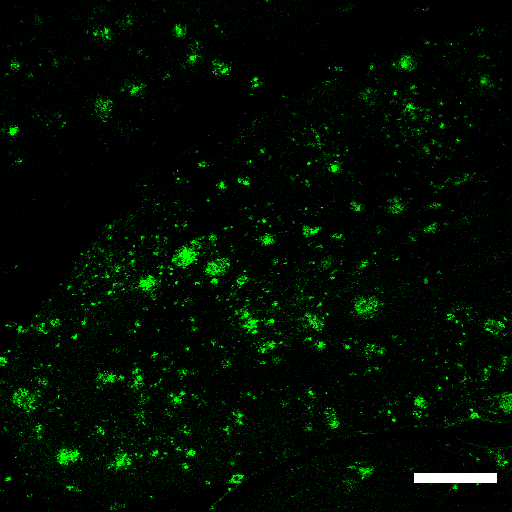

Supplement: Supplementary file 5 — Source Data for Expanded View [file EMMM-15-e17052-s012.zip › EV source data/Fig EV6/FigEV6A/14_p3-Alcb_CA3_82E1.tif]

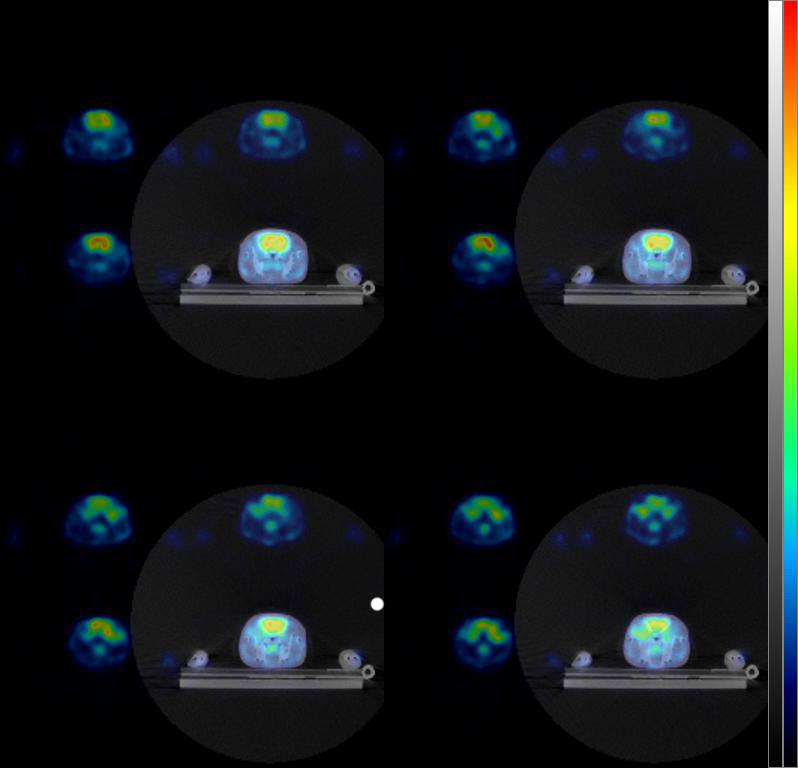

Supplement: Supplementary file 11 — Source Data for Figure 5 [file EMMM-15-e17052-s005.zip › Fig5/Fig5B/B_alc_1mg_1.tif]

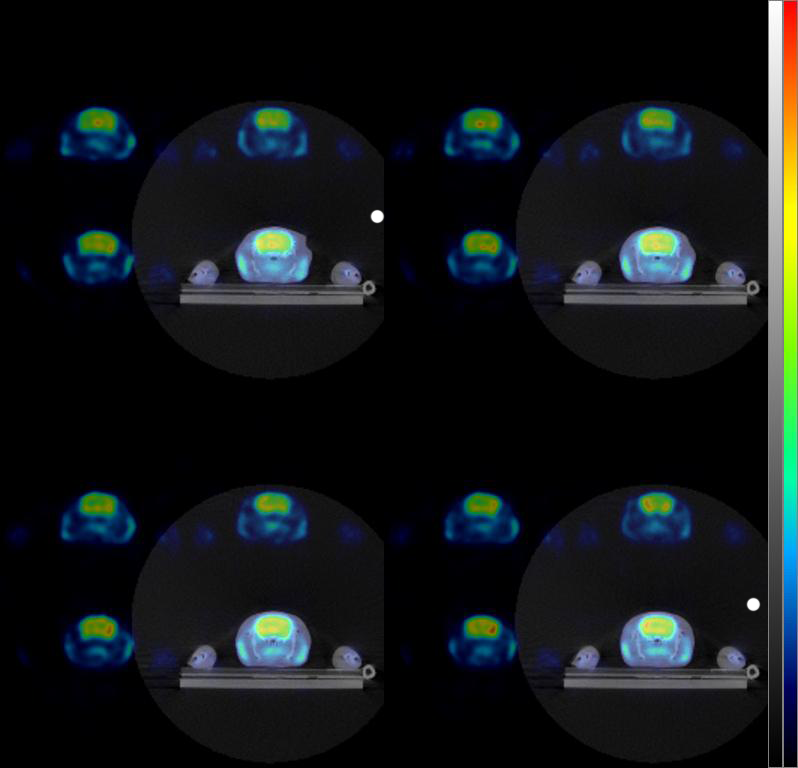

Supplement: Supplementary file 11 — Source Data for Figure 5 [file EMMM-15-e17052-s005.zip › Fig5/Fig5B/B_alc_1mg_2.tif]

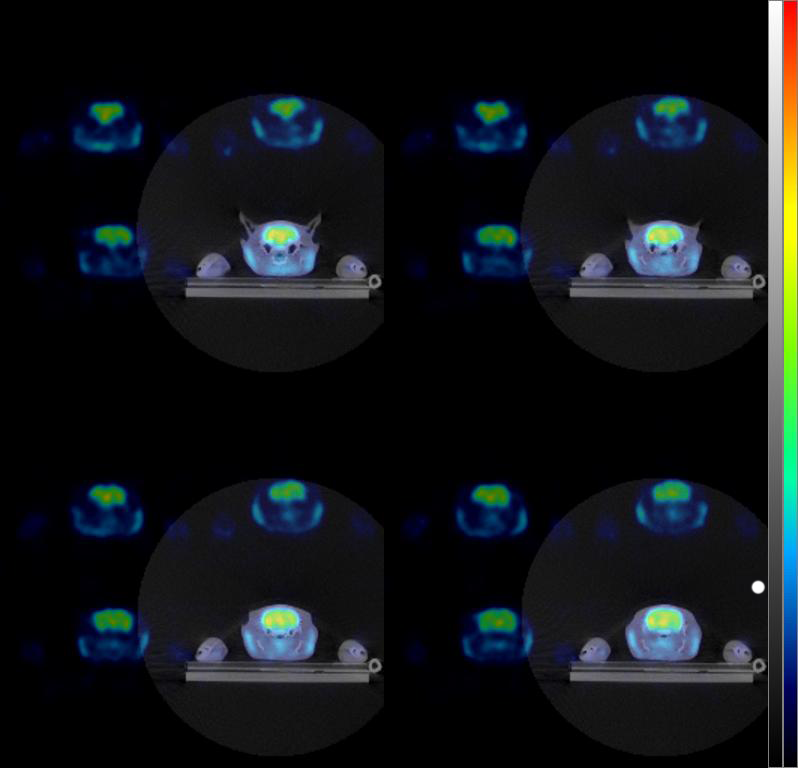

Supplement: Supplementary file 11 — Source Data for Figure 5 [file EMMM-15-e17052-s005.zip › Fig5/Fig5D/D_alc_5mg_3.tif]

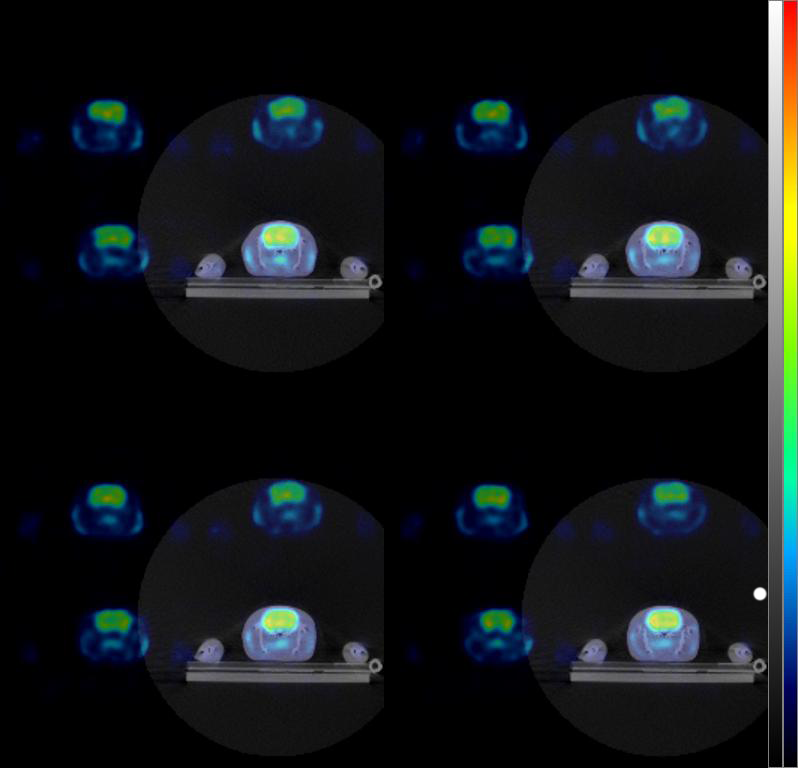

Supplement: Supplementary file 11 — Source Data for Figure 5 [file EMMM-15-e17052-s005.zip › Fig5/Fig5D/D_alc_5mg_2.tif]

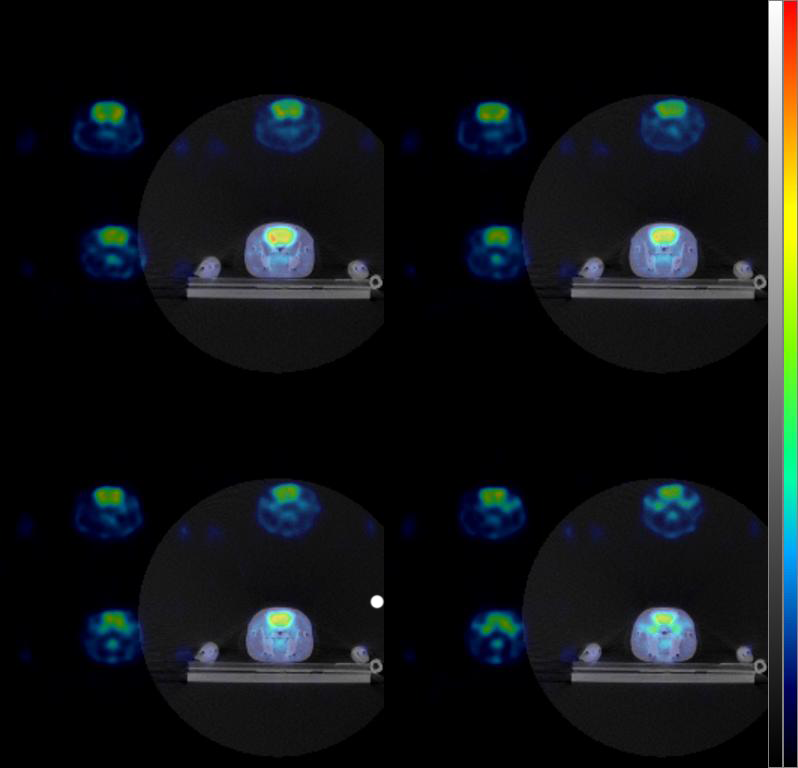

Supplement: Supplementary file 11 — Source Data for Figure 5 [file EMMM-15-e17052-s005.zip › Fig5/Fig5D/D_alc_5mg_1.tif]

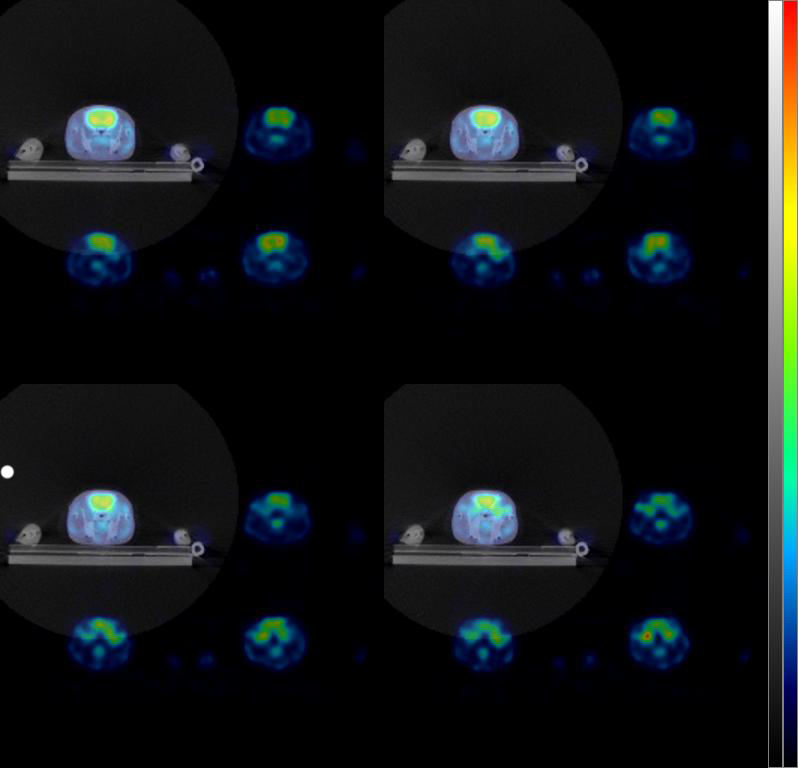

Supplement: Supplementary file 11 — Source Data for Figure 5 [file EMMM-15-e17052-s005.zip › Fig5/Fig5C/C_alc_3mg_1.tif]

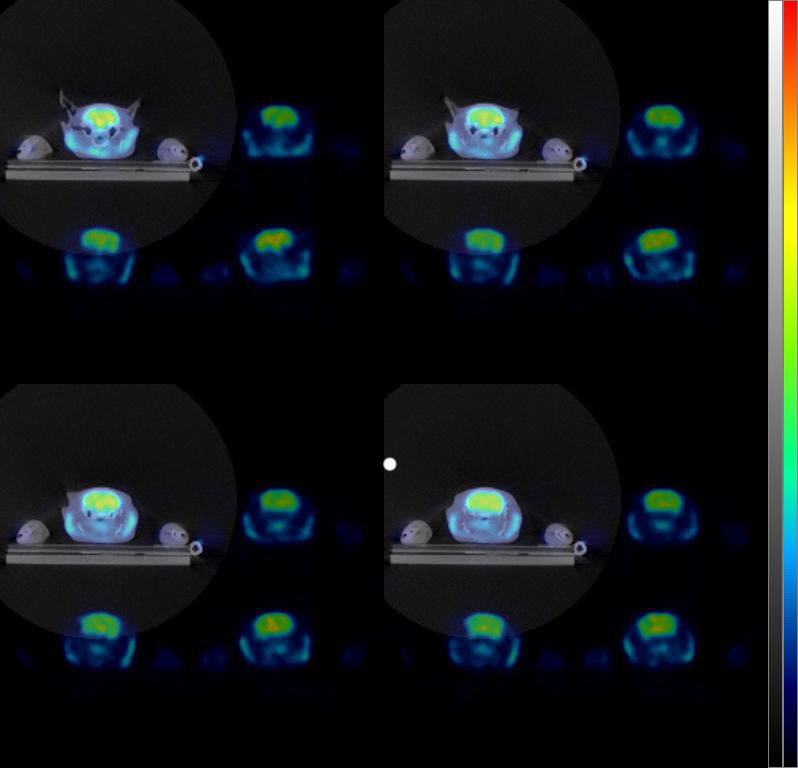

Supplement: Supplementary file 11 — Source Data for Figure 5 [file EMMM-15-e17052-s005.zip › Fig5/Fig5C/C_alc_3mg_3.tif]

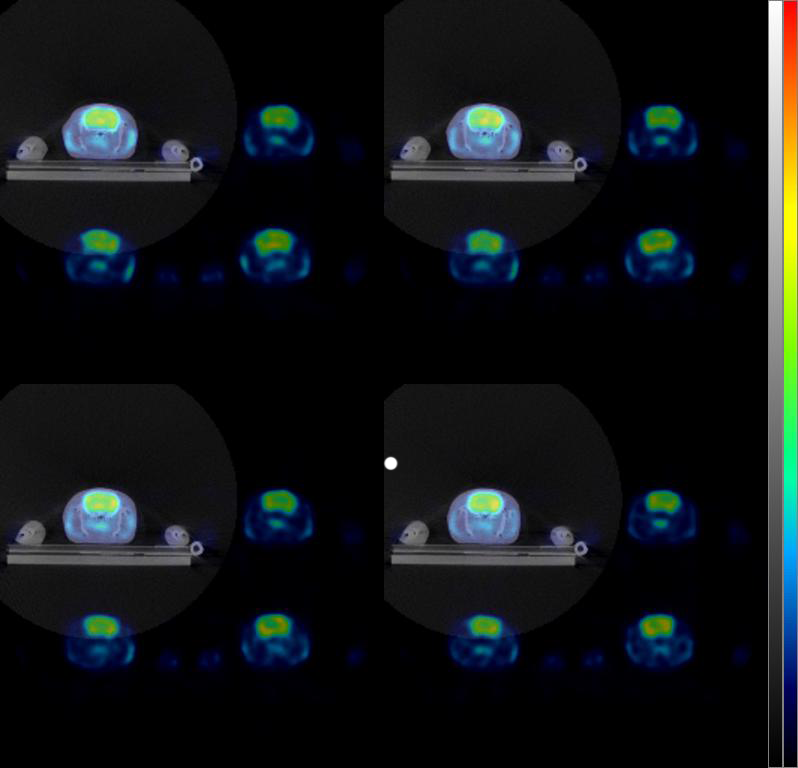

Supplement: Supplementary file 11 — Source Data for Figure 5 [file EMMM-15-e17052-s005.zip › Fig5/Fig5C/C_alc_3mg_2.tif]

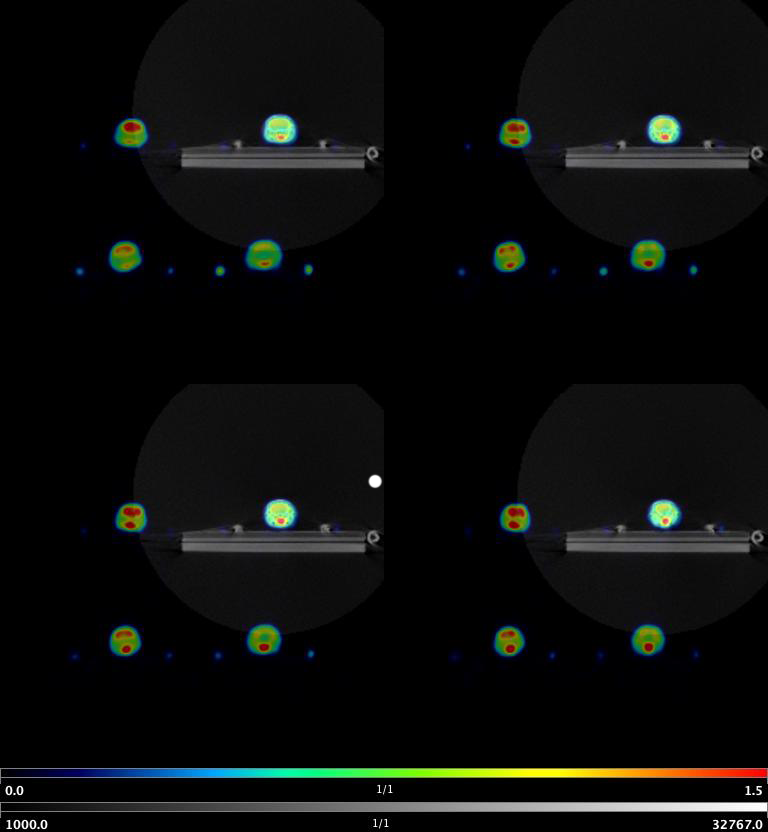

Supplement: Supplementary file 11 — Source Data for Figure 5 [file EMMM-15-e17052-s005.zip › Fig5/Fig5H/APP-p3Alc_2.tif]

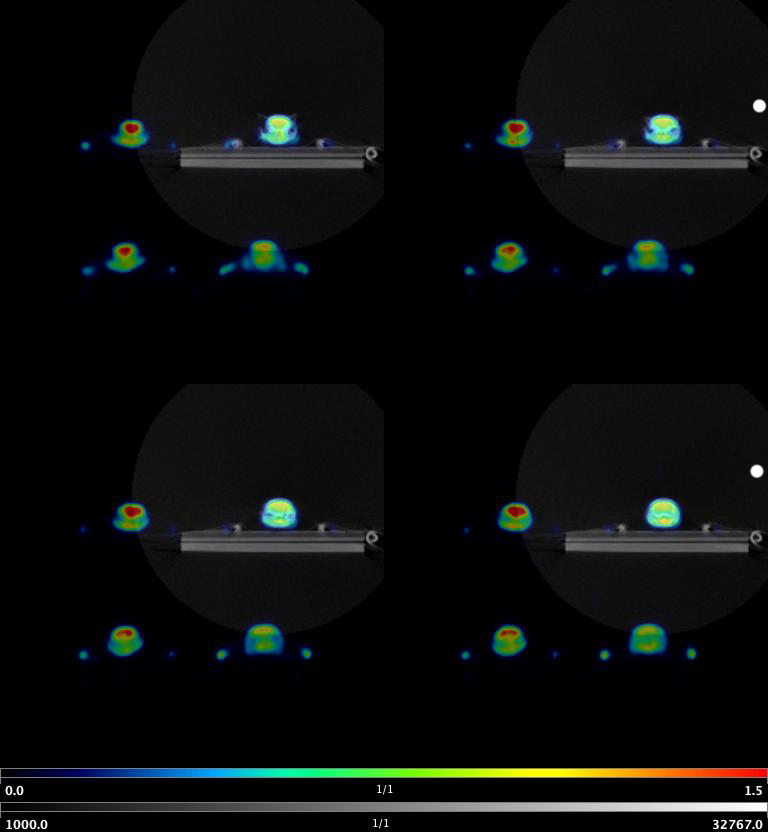

Supplement: Supplementary file 11 — Source Data for Figure 5 [file EMMM-15-e17052-s005.zip › Fig5/Fig5H/APP-p3Alc_1.tif]

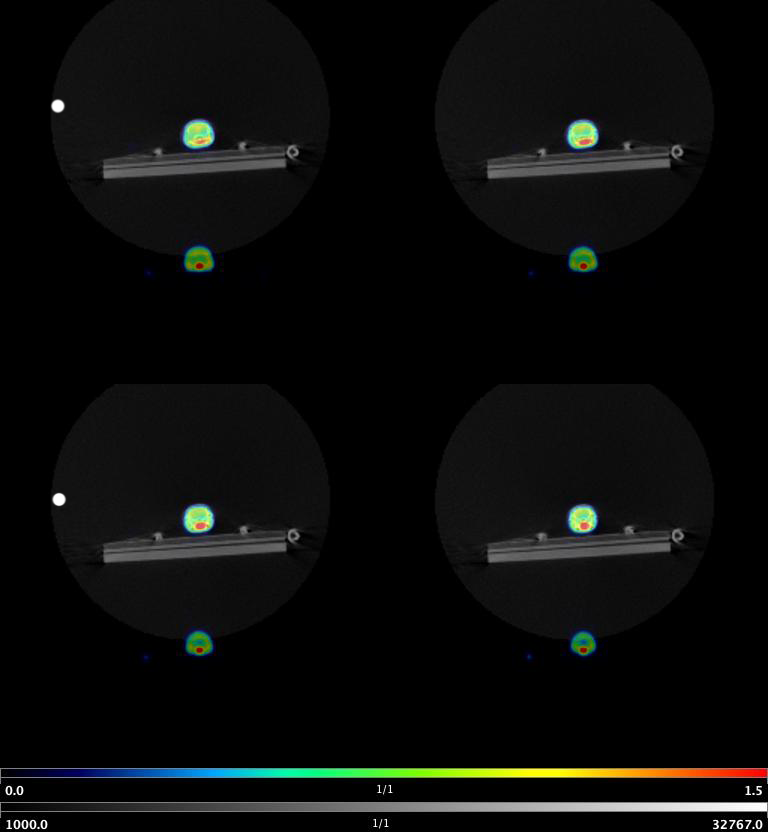

Supplement: Supplementary file 11 — Source Data for Figure 5 [file EMMM-15-e17052-s005.zip › Fig5/Fig5H/WT_2.tif]

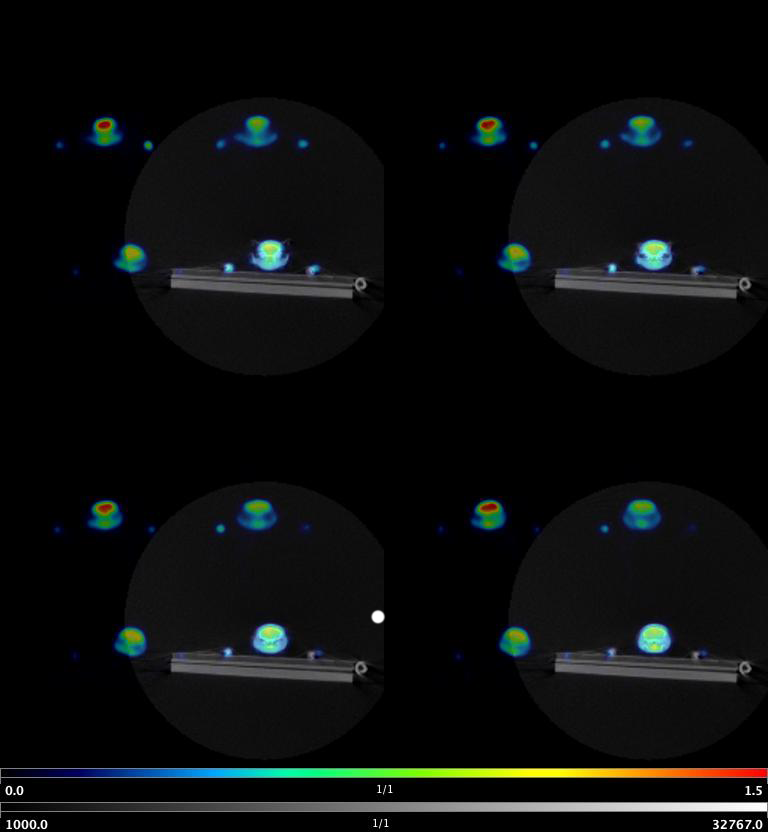

Supplement: Supplementary file 11 — Source Data for Figure 5 [file EMMM-15-e17052-s005.zip › Fig5/Fig5H/APP_1.tif]

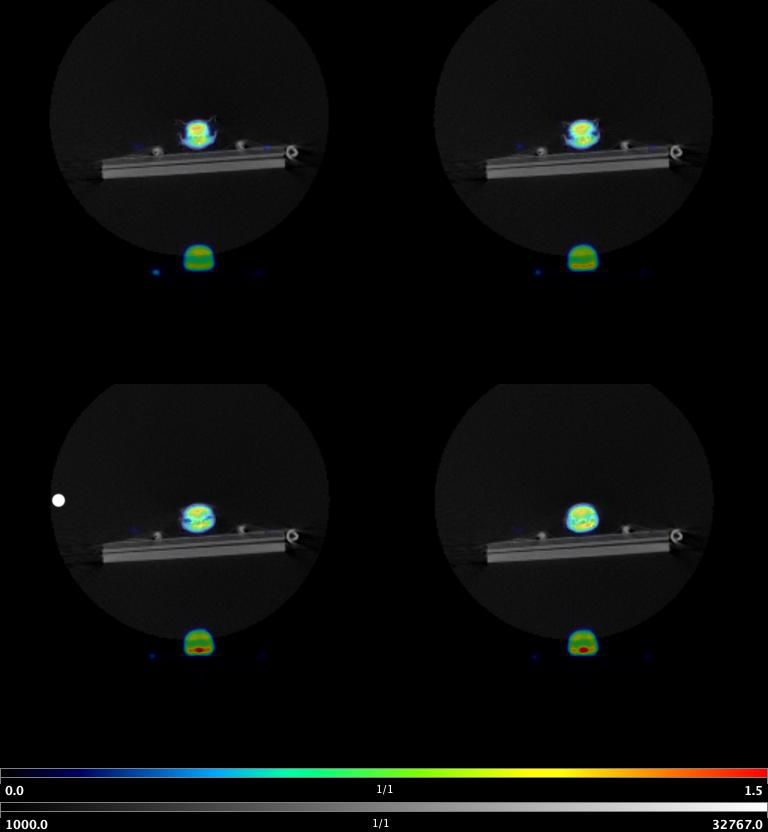

Supplement: Supplementary file 11 — Source Data for Figure 5 [file EMMM-15-e17052-s005.zip › Fig5/Fig5H/WT_1.tif]

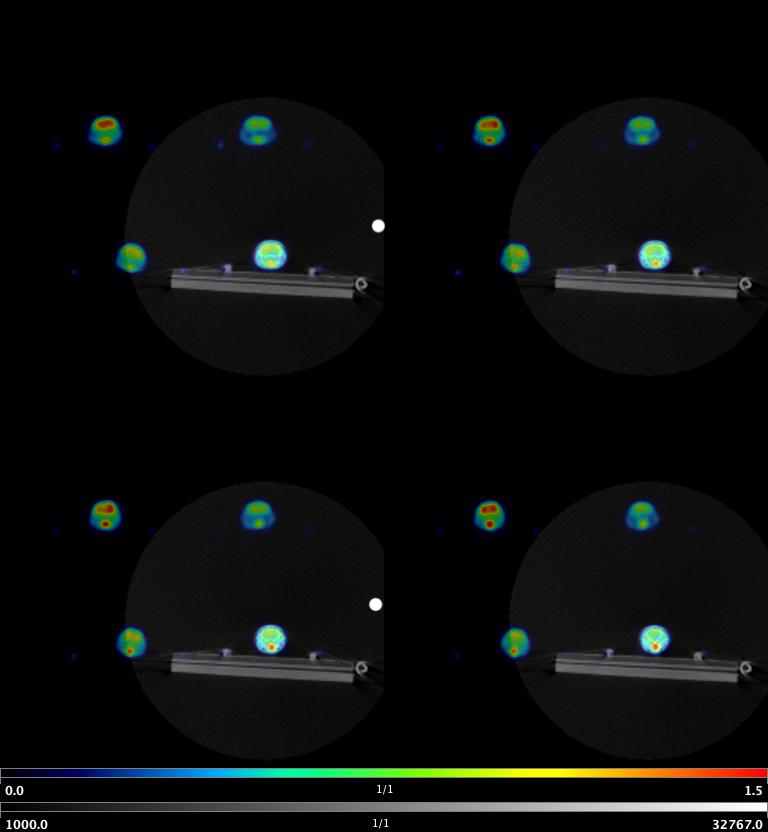

Supplement: Supplementary file 11 — Source Data for Figure 5 [file EMMM-15-e17052-s005.zip › Fig5/Fig5H/APP_2.tif]

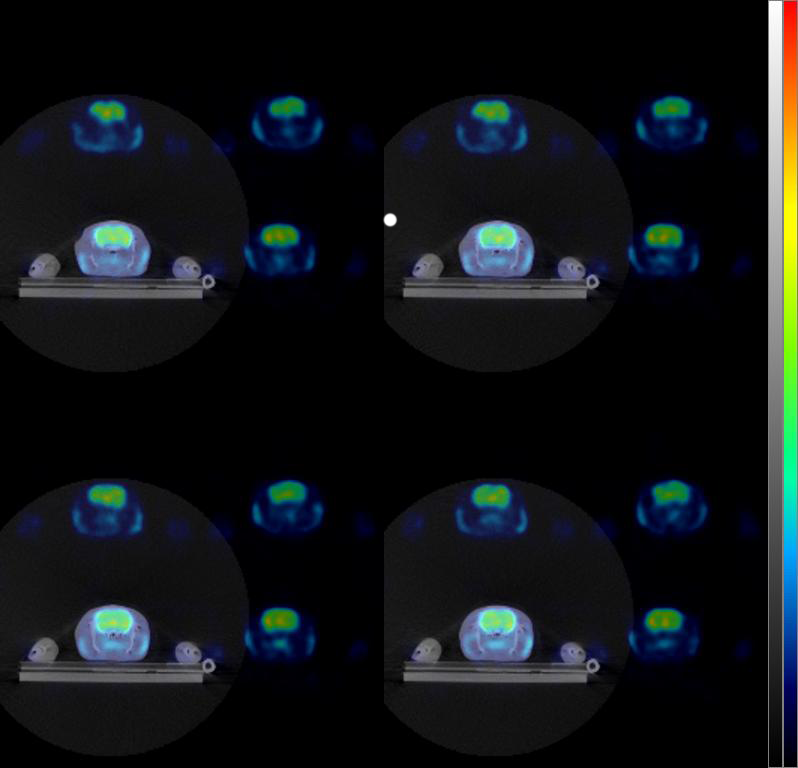

Supplement: Supplementary file 11 — Source Data for Figure 5 [file EMMM-15-e17052-s005.zip › Fig5/Fig5A/A_vehicle_2.tif]

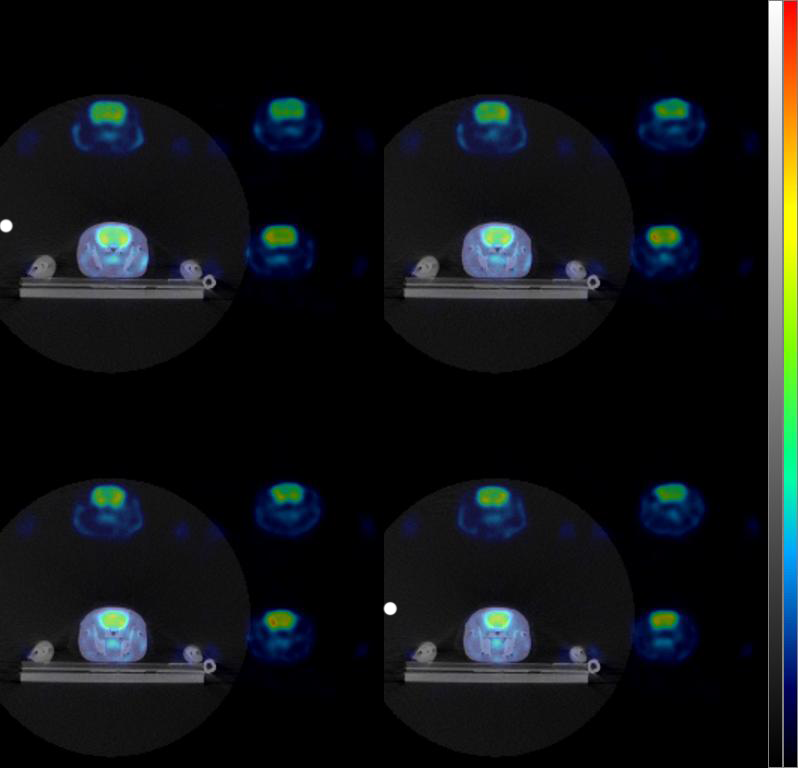

Supplement: Supplementary file 11 — Source Data for Figure 5 [file EMMM-15-e17052-s005.zip › Fig5/Fig5A/A_vehicle_1.tif]
