# Supplementary material for: Brain p3‐Alcβ peptide restores neuronal viability impaired by Alzheimer's amyloid β‐peptide
Source: EMBO Mol Med. 2023 Mar 30;15(5):e17052. doi: 10.15252/emmm.202217052 (PMC10165357; doi:10.15252/emmm.202217052)
Supplement: Supplementary file 12 [file EMMM-15-e17052-s007.pdf]

EMBO Molecular Medicine

Brain p3-Alcβ peptide restores neuronal viability impaired by Alzheimer's amyloid β-peptide

Saori Hata, Haruka Saito, Takeharu Kakiuchi, Dai Fukumoto, Shigeyuki Yamamoto, Kensaku Kasuga, Ayano Kimura, Koichi Moteki, Ruriko Abe, Shungo Adachi, Shoichi Kinoshita, Kumiko Yoshizawa-Kumagaye, Hideki Nishio, Takashi Saito, Takaomi Saido, Tohru Yamamoto, Masaki Nishimura, Hidenori Taru, Yuriko Sobu, Hiroyuki Ohba, Shingo Nishiyama, Norihiro Harada, Takeshi Ikeuchi, Hideo Tsukada, Yasuomi Ouchi, and Toshiharu Suzuki
DOI: 10.15252/emmm.202217052

Corresponding authors: Toshiharu Suzuki (tsuzuki@pharm.hokudai.ac.jp) , Yasuomi Ouchi (ouchi@hama-med.ac.jp)

Review Timeline:

Submission Date:	16th Oct 22
Editorial Decision:	22nd Nov 22
Revision Received:	23rd Jan 23
Editorial Decision:	28th Feb 23
Revision Received:	14th Mar 23
Accepted:	14th Mar 23

Editor: Jingyi Hou

Transaction Report:

(Note: With the exception of the correction of typographical or spelling errors that could be a source of ambiguity, letters and reports are not edited. Depending on transfer agreements, referee reports obtained elsewhere may or may not be included in this compilation. Referee reports are anonymous unless the Referee chooses to sign their reports.)

22nd Nov 2022

Dear Prof. Suzuki,

Thank you again for submitting your work to EMBO Molecular Medicine. We have heard back from three referees who agreed to evaluate your manuscript. As you will see from the reports below, the referees acknowledge the potential interest of the study. However, they raise a series of concerns, which we would ask you to address in a major revision of the manuscript.

I think that the referees' recommendations are relatively straightforward, so there is no need to reiterate their comments. In particular, Referee #2 mentioned that given the modest in vitro effect and in the absence of behavioral experiments, the physiological relevance of p3-Alcbeta peptide treatment remains somewhat unclear at this stage. During our pre-decision cross-commenting process (in which the referees are given a chance to make additional comments, including on each other's reports), Referees #1 and #3 made specific recommendations on this point. They both think that the result section should be revised to tone down the conclusion regarding the effects of p3-Alcbeta, and limitations of the study (including the absence of learning and memory assay) should be discussed. In light of these comments, we would still encourage you to perform animal behavioral experiments to strengthen the physiological significance of the presented findings. However, this would not be mandatory for the acceptance of the manuscript.

All other issues raised by the referees need to be satisfactorily addressed. As you may already know, our editorial policy allows in principle a single round of major revision so it is essential to provide responses to the reviewers' comments that are as complete as possible. Please feel free to contact me in case you would like to discuss in further detail any of the issues raised by the reviewers.

We would welcome the submission of a revised version within three months for further consideration. EMBO Molecular Medicine has a "scooping protection" policy, whereby similar findings that are published by others during review or revision are not a criterion for rejection. Should you decide to submit a revised version, I do ask that you get in touch after three months if you have not completed it to update us on the status.

We are aware that many laboratories cannot function at full efficiency during the current COVID-19/SARS-CoV-2 pandemic and have therefore extended our "scooping protection policy" to cover the period required for a full revision to address the experimental issues. Please let me know should you need additional time, and also if you see a paper with related content published elsewhere.

Please read below for important editorial formatting and consult our author's guidelines for proper formatting of your revised article for EMBO Molecular Medicine.

I look forward to receiving your revised manuscript.
Use this link to login to the manuscript system and submit your revision: https://embomolmed.msubmit.net/cgi-bin/main.plex

Sincerely,
Jingyi

Jingyi Hou
Editor
EMBO Molecular Medicine

*****

***** Reviewer's comments *****

Referee #1 (Remarks for Author):

Hata et. al. reported a very interesting and extensive data demonstrating neuroprotective effects of p3-Alcβ peptide on Alzheimer's disease (AD)-related Aβ oligomer-induced neurotoxicity in vitro and in vivo. Through a series of previous studies, author's group have reported that p3-Alcβ peptide is produced from neuronal protein alcadein via γ-secretase cleavage. Since γ-secretase dysfunction increases generation of toxic Aβ peptide and is thought to cause AD, authors utilized p3-Alcβ peptide levels to detect γ-secretase dysfunction. Importantly, authors demonstrated that p3-Alcβ peptide levels were significantly reduced in the cerebrospinal fluid from AD patients. These studies strongly indicate that reduced level of p3-Alcβ peptide is closely associated with AD pathogenesis.
In this study, they now provide evidence that boosting p3-Alcβ peptide level is a novel therapeutic strategy for early stage of AD. Authors showed that administration of p3-Alcβ peptide increased cellular viability by reducing Aβo-mediated excessive Ca2+ influx and enhancing mitochondrial activity using primary cultured neurons. Importantly, they demonstrated that peripheral administration successfully transferred p3-Alcβ peptides into the brain and improved the mitochondrial viability in the mouse model Aβ amyloidosis as revealed by PET imaging. Experiments are carefully and elegantly designed, and all data are solid. This reviewer thinks that this work will attract attention in the field of AD research and is a strong candidate for publication in the prestigious journal. I have a few comments as below.

Comments:

1) In Fig. 2A and 2B, the scale of y-axis seems to be different. Is there any reason for this?
2) Does p3-Alcβ 9-19 affect Aβ aggregation in vitro and Aβ pathology in vivo?
3) What is the advantage of using p3-Alcβ 9-19 over p3-Alcβ 1-37? And do p3-Alcβ 9-19 peptides exist in vivo?
4) It seems that dose dependency of neuroprotection by p3-Alcβ 9-19 is evident in vitro, but not in vivo. It would be helpful if authors can discuss this point.
5) For readability, a results section describing Figure 2 and Figure 3 may be divided into two sections with subheading.
6) In the discussion section, it would be informative to discuss the novelty of p3-Alcβ 9-19 compared to current AD therapeutics such as memantine and anti-Aβ immune therapy.

Referee #2 (Comments on Novelty/Model System for Author):

The main limitation of this paper is that p3-Alcβ peptides inhibit AβO toxicity to cultured neurons only slightly. In the absence of behavior experiments showing that p3-Alcβ prevents learning and memory deficits caused by Aβ in AD model mice, the physiological significance of the cultured neuron data is questionable.

Referee #2 (Remarks for Author):

This paper by Hata and colleagues presents evidence that proteolytic fragments of the p3-Alcβ peptide, which is produced by successive α-secretase/γ-secretase cleavage of Alcβ, inhibits various toxic effects of amyloid-β oligomers (AβOs) on neurons. Examples of AβO effects on neurons that reportedly were suppressed by p3-Alcβ include cytotoxicity, mitochondrial toxicity and excess calcium influx. Supportive data were obtained from experiments with cultured neurons, mice and rats, and led the authors to propose that p3-Alcβ has therapeutic potential for Alzheimer's disease (AD).

One conspicuous feature of most cultured neuron results shown is that effects of p3-Alcβ on AβO toxicity, though statistically significant, were typically minimal (just a few percent). This raises the obvious question of whether or not p3-Alcβ inhibits any AβO activities to a degree sufficient for physiological significance. A pertinent way to answer this question is to show that learning and memory in AD model mice is improved by p3-Alcβ, but no animal behavior experiments are included in the paper.

Many of the minimal effects of p3-Alcβ may reflect how AβOs were used to treat cultured neurons. To cite just one example, the cytotoxicity assays were performed using a 24 hour exposure to 2.5 µM AβOs. Synthetic AβOs prepared by the method used by the authors are barely cytotoxic at that concentration, though, and effectively kill neurons only at concentrations ({greater than or equal to}10 µM) orders of magnitude higher than AβO levels in vivo. Thus, the design of the cytotoxicity assays is questionable.

Beyond these serious limitations of the current version of the manuscript, the following relatively minor issues also warrant the authors' attention.

1) On page 10, in the Materials and Methods section, the authors describe how Aβ, but not p3-Alcα or p3-Alcβ peptides were oligomerized. Presumably the same procedure was used for all 3 peptides, but whatever the case may be it should be described explicitly.

2) Page 11 in the Materials and Methods section contains the following passage. "The neurons were administered 5 µL of 82 mM CaCl2 (final concentration, 2mM) when studying CaCl2 administration, or 11 µL of 100 µM Aβo in DMEM without L-glutamate including DMSO (2%), or 10 µL of DMEM with L-glutamate including DMSO (2%) for the study of Aβo and NMDA (Cat#0114, Tocris Bioscience, Bristol UK) administration." It would be helpful to readers if the final concentrations of AβOs and NMDA after their dilution into medium were also provided here.

3) Figure 3 shows western blotting evidence that Aβ1-42, but neither p3-Alcα1-35 nor p3-Alcβ1-37 forms oligomers. The blot data are clear, but they represent only one type of assay for oligomerization. It is possible that oligomers of p3-Alcα1-35, p3-Alcβ1-37 or both were formed, but in contrast to AβOs, they were sensitive to SDS. To provide more robust evidence that neither p3-Alcα1-35 nor p3-Alcβ1-37 forms oligomers the authors should use at least one additional assay, for example native gels, size exclusion chromatography, sucrose gradient ultracentrifugation or negative stain EM.

4) Please make all figures completely comprehensible without forcing readers to move their eyes back and forth between the figures and their corresponding legends. For example, instead of indicating statistical significance with asterisks, (*, **, ***, etc.) simply place the p values (as in p<0.001) directly on the figures. Similarly, in Figure EV3B, color code the labels for each row and column to correspond to their respective immunofluorescence colors (p3-Alcβ37, p3-Alcβ9-19 should use green text, and GluN2B, SYP, βIII Tub should use red text).

5) Please provide details about the microscopy used for Figure EV3 (microscope model; objective type, including magnification and NA, etc.).

6) Finally, Figure 5 depicts AβOs as causing neuronal apoptosis. That may be true in fly and worm models of AD, but most neuron death in AD is apparently not due to apoptosis.

Referee #3 (Comments on Novelty/Model System for Author):

Suzuki and colleagues discovered alcadeins nearly a decade ago. These are type 1 membrane proteins that appear to be substrates for alpha and gamma secretases as is APP. The theme that has emerged is that p3-Alz is an abeta-like fragment that is also abnormally metabolized in Alzheimer's disease (AD), but, in general, the alcadein fragments are protective rather than

being toxic. Suzuki et al have assessed alcadein in human CSF and found data that support this model. In the current paper, they demonstrate that p3-Alc can modulate pathogenesis and behavior in a mouse model of AD. The data are quite an impressive tour-de-force, including the development of a novel Alc fragment ELISA. The data are clear and convincing, and the conclusions are appropriately cautious. There are no ethical issues.

Referee #3 (Remarks for Author):

I support publication in whatever form the editor prefers. I could imagine publishing only half of the text and moving the methods text and figures to an online supplement.

***** Reviewer's comments *****

Referee #1 (Remarks for Author):

Hata et. al. reported a very interesting and extensive data demonstrating neuroprotective effects of p3-Alcβ peptide on Alzheimer's disease (AD)-related Aβ oligomer-induced neurotoxicity in vitro and in vivo. Through a series of previous studies, author's group have reported that p3-Alcβ peptide is produced from neuronal protein alcadein via γ-secretase cleavage. Since γ-secretase dysfunction increases generation of toxic Aβ peptide and is thought to cause AD, authors utilized p3-Alcβ peptide levels to detect γ-secretase dysfunction. Importantly, authors demonstrated that p3-Alcβ peptide levels were significantly reduced in the cerebrospinal fluid from AD patients. These studies strongly indicate that reduced level of p3-Alcβ peptide is closely associated with AD pathogenesis.
In this study, they now provide evidence that boosting p3-Alcβ peptide level is a novel therapeutic strategy for early stage of AD. Authors showed that administration of p3-Alcβ peptide increased cellular viability by reducing Aβo-mediated excessive Ca2+ influx and enhancing mitochondrial activity using primary cultured neurons. Importantly, they demonstrated that peripheral administration successfully transferred p3-Alcβ peptides into the brain and improved the mitochondrial viability in the mouse model Aβ amyloidosis as revealed by PET imaging. Experiments are carefully and elegantly designed, and all data are solid. This reviewer thinks that this work will attract attention in the field of AD research and is a strong candidate for publication in the prestigious journal. I have a few comments as below.

Comments:

1) In Fig. 2A and 2B, the scale of y-axis seems to be different. Is there any reason for this?

Response: The lower reactivity of neurons in Fig. 2A may be caused by the condition of primary cultured neurons. We re-examined the effect of p3-Alcβ9-19 using a new set of neurons. In terms of the scale magnitude of the y-axis, our new finding is comparable to the result in Fig. 2B. We have replaced the old Fig. 2A with the new result.

2) Does p3-Alcβ 9-19 affect Aβ aggregation in vitro and Aβ pathology in vivo?

Response: In a study with Thioflavin T, Aβ42 aggregation tends to be slightly, and not significantly, suppressed in the presence of a higher concentration of either p3-Alcα35 or p3-Alcβ37 (**Unpublished observation 10**). The suppression of Aβ42 aggregation by p3-Alcβ37 was comparable to that by p3-Alcα35. Therefore, we think that the presence of other peptides may non-specifically influence the aggregation of Aβ42 in vitro.

In a separate study, we administered p3-Alcβ9-19 (1 mg/kg weight) subcutaneously into 9-month-old APP-KI (*App*$^{NL\text{-}G\text{-}F/NL\text{-}G\text{-}F}$) mice daily for 30 days and immunostained amyloid plaques in the hippocampus

and cerebral cortex (**Unpublished observation 8**). The area occupied by amyloid plaques (% of total area) tended to decrease in the hippocampus of p3-Alcβ9-19-treated mice but was not significant compared to control (PBS-administered) mice. In this AD mouse model (*App*$^{NL\text{-}G\text{-}F/NL\text{-}G\text{-}F}$), Aβ42 begins accumulating in the brain at 3 months of age and reaches a saturated level at 5 months of age (*Neurobiol. Aging*,.2023.123 63-74). Therefore, the data suggest that p3-Alcβ9-19 is ineffective at removing accumulated/aggregated Aβ in the brain, which is distinct from the effect of therapy with an anti-Aβ antibody.

Furthermore, we quantified Aβ42 levels in the cerebral cortex and hippocampus of 9-month-old *App*$^{NL\text{-}G\text{-}F/NL\text{-}G\text{-}F}$ mice that were subcutaneously administered p3-Alcβ9-19 (1mg/kg body weight) daily for 30 days as described above. The level of Aβ42 in mice that were administered p3-Alcβ9-19 was identical to that in PBS-injected control mice (**Unpublished observation 9**).

Taken together (Unpublished observations 8 and 9 in vivo with Unpublished observations 10 in vitro), we think that the effects of p3-Alcβ on Aβ aggregation in vitro and on amyloid clearance in vivo are limited if any. Moreover, and as described in our text, we would like to emphasize that p3-Alcβ increases neuronal viability and protects neurons against Aβ-induced toxicity, and that the direct targets of p3-Alcβ are not Aβ peptide and Aβ aggregates/plaques.

3) What is the advantage of using p3-Alcβ 9-19 over p3-Alcβ 1-37? And do p3-Alcβ 9-19 peptides exist in vivo?

Response: The advantages of using p3-Alcβ9-19 are its smaller size, improved pharmacokinetics in blood and CSF, and its low cost as the drug substance compared to p3-Alcβ1-37 synthesis. We also confirmed that p3-Alcβ1-37 was transported into the brain via subcutaneous administration in Alcβ-KO mice, which do not generate endogenous p3-Alcβ1-37. p3-Alcβ9-19 peptides are not generated physiologically in vivo. We determined the pharmacokinetics of p3-Alcβ9-19 in rats and monkeys. The data were obtained for drug development. Since endogenous p3-Alcβ1-37 is detected in the plasma and CSF of humans, monkeys, and rodents, the use of p3-Alcβ9-19 will yield reliable results for p3-Alcβ9-19 pharmacokinetics even in the presence of endogenous p3-Alcβ peptides in the blood and CSF.

4) It seems that dose dependency of neuroprotection by p3-Alcβ 9-19 is evident in vitro, but not in vivo. It would be helpful if authors can discuss this point.

Response: In the PET imaging study with rats (**Fig. 4A-G**), we performed a dose-response analysis of 1 mg, 3 mg, and 5 mg p3-Alcβ9-19 per body weight (kg). The administration of p3-Alcβ9-19 (1 mg/kg body weight) significantly increased mitochondrial function in the brain.

Furthermore, in the behavioral test, we found that peripherally administered doses of 1.0 mg/kg p3-Alcβ9-19 per body weight were effective at restoring memory (**Unpublished observations for review** by a collaborator). Therefore, we used 1.0 mg of p3-Alcβ9-19/kg weight in our animal studies and have described it in the Discussion section as unpublished comments.

5) For readability, a results section describing Figure 2 and Figure 3 may be divided into two sections with subheading.

Response: The results section has been separated in the revised version of the manuscript.

6) In the discussion section, it would be informative to discuss the novelty of p3-Alcβ 9-19 compared to current AD therapeutics such as memantine and anti-Aβ immune therapy.

Response: As per our response to Question 2, we have discussed the novelty of p3-Alcβ 9-19 in the Discussion.

Referee #2 (Comments on Novelty/Model System for Author):

The main limitation of this paper is that p3-Alcβ peptides inhibit AβO toxicity to cultured neurons only slightly. In the absence of behavior experiments showing that p3-Alcβ prevents learning and memory deficits caused by Aβ in AD model mice, the physiological significance of the cultured neuron data is questionable.

Referee #2 (Remarks for Author):

This paper by Hata and colleagues presents evidence that proteolytic fragments of the p3-Alcβ peptide, which is produced by successive α-secretase/γ-secretase cleavage of Alcβ, inhibits various toxic effects of amyloid-β oligomers (AβOs) on neurons. Examples of AβO effects on neurons that reportedly were suppressed by p3-Alcβ include cytotoxicity, mitochondrial toxicity and excess calcium influx. Supportive data were obtained from experiments with cultured neurons, mice and rats, and led the authors to propose that p3-Alcβ has therapeutic potential for Alzheimer's disease (AD).

One conspicuous feature of most cultured neuron results shown is that effects of p3-Alcβ on AβO toxicity, though statistically significant, were typically minimal (just a few percent). This raises the obvious question of whether or not p3-Alcβ inhibits any AβO activities to a degree sufficient for physiological significance. A pertinent way to answer this question is to show that learning and memory in AD model mice is improved by p3-Alcβ, but no animal behavior experiments are included in the paper.

Response: We have performed preliminary animal behavior experiments (These results are shown in **Unpublished observations** for review only). We used the acute AD mouse model instead of AD mice models. Aβ42 oligomers, which can induce cognitive impairment, were injected into the lateral ventricles in wild-type mice. Injection of p3-Alcβ peptide into the lateral ventricles or intraperitoneally ameliorated Aβ42

oligomer-induced memory impairment. These experiments were performed in the laboratory of our collaborator. The collaborative team will attempt more behavioral studies with AD mouse models, but the current COVID-19/SARS-CoV-2 pandemic during the last couple of years made it difficult to maintain the mice population and conduct additional and extensive behavioral tests. Nevertheless, we believe that Unpublished observations demonstrate that p3-Alcβ9-19 ameliorates the memory impairment induced by Aβ42 oligomers.

The collaborative study team will publish their behavior analyses, together with further detailed analyses, as a separate paper in the future.

Many of the minimal effects of p3-Alcβ may reflect how AβOs were used to treat cultured neurons. To cite just one example, the cytotoxicity assays were performed using a 24-hour exposure to 2.5 μM AβOs. Synthetic AβOs prepared by the method used by the authors are barely cytotoxic at that concentration, though, and effectively kill neurons only at concentrations ({greater than or equal to}10 μM) orders of magnitude higher than AβO levels in vivo. Thus, the design of the cytotoxicity assays is questionable.

Response: We examined neurotoxicity using a higher concentration of Aβo (Unpublished observation 11 for the review only). As suggested by the reviewer, a higher concentration of Aβo (10 μM) exhibits stronger neurotoxicity that severely impairs cell viability than a lower concentration (2.5 μM) of Aβo. In the presence of 10 μM of Aβo, p3-Alcβ peptide (10 μM) tended to restore neuronal viability, but this was insufficient. Therefore, we chose 2.5 μM of Aβo to show weak toxicity, where neuronal viability can be significantly restored from the impairment by administering 10 μM of either p3-Alcβ37 or p3-Alcβ9-19.

Beyond these serious limitations of the current version of the manuscript, the following relatively minor issues also warrant the authors' attention.

1) On page 10, in the Materials and Methods section, the authors describe how Aβ, but not p3-Alcα or p3-Alcβ peptides were oligomerized. Presumably the same procedure was used for all 3 peptides, but whatever the case may be it should be described explicitly.

Response: We have not described the preparation of p3-Alc solution. The description is the procedure used to prepare Aβ42 oligomers. Since p3-Alc peptides are non-aggregative (Fig. EV1B and C, and we show a new result in Unpublished observation 2), we dissolved p3-Alc peptides in DMSO. We have corrected this description in Materials and methods.

2) Page 11 in the Materials and Methods section contains the following passage. "The neurons were administered 5 μL of 82 mM CaCl2 (final concentration, 2mM) when studying CaCl2 administration, or 11 μL

of 100 µM Aβo in DMEM without L-glutamate including DMSO (2%), or 10 µL of DMEM with L-glutamate including DMSO (2%) for the study of Aβo and NMDA (Cat#0114, Tocris Bioscience, Bristol UK) administration." It would be helpful to readers if the final concentrations of AβOs and NMDA after their dilution into medium were also provided here.

Response: We have now clearly rewritten the procedure and have provided the final concentrations of Aβo and NMDA.

3) Figure 3 shows western blotting evidence that Aβ1-42, but neither p3-Alcα1-35 nor p3-Alcβ1-37 forms oligomers. The blot data are clear, but they represent only one type of assay for oligomerization. It is possible that oligomers of p3-Alcα1-35, p3-Alcβ1-37 or both were formed, but in contrast to AβOs, they were sensitive to SDS. To provide more robust evidence that neither p3-Alcα1-35 nor p3-Alcβ1-37 forms oligomers the authors should use at least one additional assay, for example native gels, size exclusion chromatography, sucrose gradient ultracentrifugation or negative stain EM.

Response: First, we monitored peptide aggregation with Thioflavin T (**Unpublished observation 2 for review only**). As expected, Aβ42 formed aggregates, which showed strong fluorescence intensity that increased with incubation time. The fluorescence intensities of both p3-Alcα35 and p3-Alcβ37 were below the threshold and did not increase until the incubation time point of 24 h.

Second, p3-Alcβ37 was monitored by size-exclusion chromatography (**Fig. EV1C in the revised version**). p3-Alcβ37 (50 µM, diluted in PBS) was incubated for 0, 2, 4, and 24 h at 37°C, and the peptides were then subjected to size-exclusion chromatography with PBS. At all incubation time points, a single peak of p3-Alcβ37 with molecular size ~4,000 was detected, and no oligomers were detected. This result indicates that p3-Alcβ37 did not aggregate in PBS.

4) Please make all figures completely comprehensible without forcing readers to move their eyes back and forth between the figures and their corresponding legends. For example, instead of indicating statistical significance with asterisks, (*, **, ***, etc.) simply place the p values (as in p<0.001) directly on the figures. Similarly, in Figure EV3B, color code the labels for each row and column to correspond to their respective immunofluorescence colors (p3-Alcβ37, p3-Alcβ9-19 should use green text, and GluN2B, SYP, βIII Tub should use red text).

Response: We have placed the *p*-values directly on the figures.

5) Please provide details about the microscopy used for Figure EV3 (microscope model; objective type, including magnification and NA, etc.).

Response: We have described information about the microscope in the section on “Photo-affinity labeling of neuronal proteins in cultured neurons with p3-Alcβ9-19 and p3-Alcβ37, and colocalization with neuronal proteins” in the Materials and methods.

6) Finally, Figure 5 depicts AβOs as causing neuronal apoptosis. That may be true in fly and worm models of AD, but most neuron death in AD is apparently not due to apoptosis.

Response: We have now used the term “Cell death pathway” rather than “Apoptotic pathway.” Fig. 5 has been replaced with a new one.

Referee #3 (Comments on Novelty/Model System for Author):

Suzuki and colleagues discovered alcadeins nearly a decade ago. These are type 1 membrane proteins that appear to be substrates for alpha and gamma secretases as is APP. The theme that has emerged is that p3-Alz is an abeta-like fragment that is also abnormally metabolized in Alzheimer's disease (AD), but, in general, the alcadein fragments are protective rather than being toxic. Suzuki et al have assessed alcadein in human CSF and found data that support this model. In the current paper, they demonstrate that p3-Alc can modulate pathogenesis and behavior in a mouse model of AD. The data are quite an impressive tour-de-force, including the development of a novel Alc fragment ELISA. The data are clear and convincing, and the conclusions are appropriately cautious. There are no ethical issues.

Referee #3 (Remarks for Author):

I support publication in whatever form the editor prefers. I could imagine publishing only half of the text and moving the methods text and figures to an online supplement.

Response: Thank you very much for your positive remark about our research. The manuscript size is standard and is not bigger compared to other publications that are currently published in this journal. Based on my understanding, the methods text is located along the main text and figures to help, and for the benefit of, the readers. Furthermore, the editor recommends only five expanded-view figures. In the first submission, we had already attached seven expanded-view figures, which have been condensed into the five expanded-view figures in the revised version of the manuscript. I would like to keep the current structure.

28th Feb 2023

Dear Prof. Suzuki,

Thank you for the submission of your revised manuscript to EMBO Molecular Medicine. We have now received the enclosed report from two referees who were asked to re-assess it. As you will see, the referees are now overall supportive, and I am pleased to inform you that we will be able to accept your manuscript pending the following amendments:

1. Please note that according to our guidelines on "Unpublished Data", the journal does not permit citation of "data not shown" or" unpublished data". All data referred to in the paper should be displayed in the main or Expanded View figures. Further, considering the comments of Referee #2, we would encourage you to include the "unpublished observation for Reviewer" in the manuscript (except for the preliminary animal behavior experiments). Citations of unpublished data should be removed from the manuscript file.

2. Remove the 'Author contribution' section from the manuscript file.

3. Funding information seems to be incomplete in the online submission system. Please ensure the information entered in the submission system is consistent with that in the manuscript file.

4. Appendix file: page numbers are needed in the Table of Content.

5. Reference format: Please list up to 10 co-authors of a paper before adding et al. to the reference list. Remove DOI links for published papers.

6. We noticed that you provided source data for EV figures, and the list goes up to Fig EV7, but there are only 5 EV figures provided. Please clarify this and upload the source data for EV figures as one file per figure.

7. As part of the EMBO Publications transparent editorial process initiative (see our Editorial at http://embomolmed.embopress.org/content/2/9/329), EMBO Molecular Medicine will publish online a Review Process File (RPF) to accompany accepted manuscripts.

In the event of acceptance, this file will be published in conjunction with your paper and will include the anonymous referee reports, your point-by-point response and all pertinent correspondence relating to the manuscript. Let us know if you disagree with this.

Please note that the Authors checklist will be published at the end of the RPF.

8. In the synopsis text, please add a short stand first (maximum of 300 characters, including space). Please use the passive voice.

9. Our data editor has made some suggestions to your manuscript (please see attached), which we would like to ask you to address (along with other issues) before you submit your revised manuscript. Please use this version to make your changes on the paper and keep the track mode on.

Please submit your revised manuscript within two weeks. I look forward to seeing a revised version of your manuscript as soon as possible.

Sincerely,
Jingyi

Jingyi Hou
Editor
EMBO Molecular Medicine

*** Instructions to submit your revised manuscript ***

*** PLEASE NOTE *** As part of the EMBO Publications transparent editorial process initiative (see our Editorial at https://www.embopress.org/doi/pdf/10.1002/emmm.201000094), EMBO Molecular Medicine will publish online a Review Process File to accompany accepted manuscripts.

In the event of acceptance, this file will be published in conjunction with your paper and will include the anonymous referee reports, your point-by-point response and all pertinent correspondence relating to the manuscript. If you do NOT want this file to be published, please inform the editorial office at contact@embomolmed.org.

To submit your manuscript, please follow this link:

https://embomolmed.msubmit.net/cgi-bin/main.plex

When submitting your revised manuscript, please include:

1) a .docx formatted version of the manuscript text (including Figure legends and tables)

2) Separate figure files*

3) supplemental information as Expanded View and/or Appendix. Please carefully check the authors guidelines for formatting Expanded view and Appendix figures and tables at
https://www.embopress.org/page/journal/17574684/authorguide#expandedview

4) a letter INCLUDING the reviewer's reports and your detailed responses to their comments (as Word file).

5) The paper explained: EMBO Molecular Medicine articles are accompanied by a summary of the articles to emphasize the major findings in the paper and their medical implications for the non-specialist reader. Please provide a draft summary of your article highlighting
- the medical issue you are addressing,
- the results obtained and
- their clinical impact.
This may be edited to ensure that readers understand the significance and context of the research.
Please refer to any of our published articles for an example.

6) For more information: There is space at the end of each article to list relevant web links for further consultation by our readers. Could you identify some relevant ones and provide such information as well? Some examples are patient associations, relevant databases, OMIM/proteins/genes links, author's websites, etc...

7) EMBO Molecular Medicine now requires a complete author checklist (https://www.embopress.org/page/journal/17574684/authorguide) to be submitted with all revised manuscripts. Please use the checklist as guideline for the sort of information we need WITHIN the manuscript. The checklist should only be filled with page numbers were the information can be found. This is particularly important for animal reporting, antibody dilutions (missing) and exact values and n that should be indicted instead of a range.

8) Every published paper now includes a 'Synopsis' to further enhance discoverability. Synopses are displayed on the journal webpage and are freely accessible to all readers. They include a short stand first (maximum of 300 characters, including space) as well as 2-5 one sentence bullet points that summarise the paper. Please write the bullet points to summarise the key NEW findings. They should be designed to be complementary to the abstract - i.e. not repeat the same text. We encourage inclusion of key acronyms and quantitative information (maximum of 30 words / bullet point). Please use the passive voice. Please attach these in a separate file or send them by email, we will incorporate them accordingly.

You are also welcome to suggest a striking image or visual abstract to illustrate your article. If you do please provide a jpeg file 550 px-wide x 400-px high.

9) A Conflict of Interest statement should be provided in the main text

10) Please note that we now mandate that all corresponding authors list an ORCID digital identifier. This takes <90 seconds to complete. We encourage all authors to supply an ORCID identifier, which will be linked to their name for unambiguous name identification.

Currently, our records indicate that the ORCID for your account is 0000-0002-1655-2374.

Please click the link below to modify this ORCID:

Link Not Available

11) The system will prompt you to fill in your funding and payment information. This will allow Wiley to send you a quote for the article processing charge (APC) in case of acceptance. This quote takes into account any reduction or fee waivers that you may be eligible for. Authors do not need to pay any fees before their manuscript is accepted and transferred to our publisher.

*Additional important information regarding Figures

Each figure should be given in a separate file and should have the following resolution:
Graphs 800-1,200 DPI
Photos 400-800 DPI
Colour (only CMYK) 300-400 DPI"

Figures are not edited by the production team. All lettering should be the same size and style; figure panels should be indicated by capital letters (A, B, C etc). Gridlines are not allowed except for log plots. Figures should be numbered in the order of their appearance in the text with Arabic numerals. Each Figure must have a separate legend and a caption is needed for each panel.

*Additional important information regarding figures and illustrations can be found at https://bit.ly/EMBOPressFigurePreparationGuideline. See also figure legend preparation guidelines: https://www.embopress.org/page/journal/17574684/authorguide#figureformat

The system will prompt you to fill in your funding and payment information. This will allow Wiley to send you a quote for the article processing charge (APC) in case of acceptance. This quote takes into account any reduction or fee waivers that you may be eligible for. Authors do not need to pay any fees before their manuscript is accepted and transferred to our publisher.

***** Reviewer's comments *****

Referee #1 (Remarks for Author):

Authors addressed this reviewer's comments adequately.

Referee #2 (Comments on Novelty/Model System for Author):

The auithors used an appropriate mix of animal models (rat and mouse), and in vitro assays to test the potential use of p3-Alcβ for Alzheimer's disease.

Referee #2 (Remarks for Author):

As reviewer #1 of the original submission, my main criticism of the paper was that the results provided were overinterpreted. More specifically, while p3-Alcβ clearly provided protection against excess calcium influx and impaired mitochondrial activity in neurons, its effects on Aβo-induced cytotoxicity for cultured neurons were marginal, and effects on learning and memory, the ultimate test of any drug that might be successful against AD, were not addressed. In the authors' favor, subcutaneous injection of p3-Alcβ into APP-KI mice led to stronger SUVR signals from the [18F]BCPP-EF PET ligand than injection of vehicle (Figure 4), which is consistent with p3-Alcβ protecting against Aβ-mediated neuron death. [18F]BCPP-EF detects mitochondrial complex I, and though by extension it may indirectly measure neuronal viability, it is not at all clear how accurately it does so.

The revised paper does not change my opinion about those issues, but evaluating the paper by additional criteria does paint a somewhat different picture. For example, the study seems to have been very carefully designed and conducted, and the data are of very high quality. The fact that the data do not support stunning protection of cultured neurons by p3-Alcβ against Aβo-induced cell death is not the authors' fault, and those data are certainly worthy of publication because AD researchers will benefit from knowing about all of the experiments described in the paper. That said, I feel that adding to the paper the behavioral data that were provided to reviewers in the "Unpublished observations for review" attachment would substantially elevate the strength of the paper's message about p3-Alcβ. In short, the science is solid, but key results regarding protection against Aβ cytotoxicity are unimpressive for cultured neurons and ambiguous in vivo, and adding the behavioral data already in hand would alleviate that concern.

***** Reviewer's comments *****

Referee #1 (Remarks for Author):

Authors addressed this reviewer's comments adequately.

Response: Thank you very much for your positive remark about our research and your encouragement to the publication of the manuscript.

Referee #2 (Remarks for Author):

As reviewer #1 of the original submission, my main criticism of the paper was that the results provided were overinterpreted. More specifically, while p3-Alcβ clearly provided protection against excess calcium influx and impaired mitochondrial activity in neurons, its effects on Aβo-induced cytotoxicity for cultured neurons were marginal, and effects on learning and memory, the ultimate test of any drug that might be successful against AD, were not addressed. In the authors' favor, subcutaneous injection of p3-Alcβ into APP-KI mice led to stronger SUVR signals from the [18F]BCPP-EF PET ligand than injection of vehicle (Figure 4), which is consistent with p3-Alcβ protecting against Aβ-mediated neuron death. [18F]BCPP-EF detects mitochondrial complex I, and though by extension it may indirectly measure neuronal viability, it is not at all clear how accurately it does so.

The revised paper does not change my opinion about those issues, but evaluating the paper by additional criteria does paint a somewhat different picture. For example, the study seems to have been very carefully designed and conducted, and the data are of very high quality. The fact that the data do not support stunning protection of cultured neurons by p3-Alcβ against Aβo-induced cell death is not the authors' fault, and those data are certainly worthy of publication because AD researchers will benefit from knowing about all of the experiments described in the paper. That said, I feel that adding to the paper the behavioral data that were provided to reviewers in the "Unpublished observations for review" attachment would substantially elevate the strength of the paper's message about p3-Alcβ. In short, the science is solid, but key results regarding protection against Aβ cytotoxicity are unimpressive for cultured neurons and ambiguous in vivo, and adding the behavioral data already in hand would alleviate that concern.

Response: First, we appreciate your review and comments that improve our manuscript and thank you very much for your evaluation of our manuscript with additional insights. We inserted unpublished observations into the figures and the expanded view figures to alleviate reviewers'' concerns. As described in the response letter of the first revised version, the results of animal behavior experiments, which are performed independently by collaborators, are preliminary and were suspended by the current COVID-19/SARS-CoV-2 pandemic. Although we showed one type of animal behavior experiment as unpublished

observations for reviewers' understanding of the effects of p3-Alcβ in animal studies, we would like to exclude these from the second revised version because the results are preliminary observations to open in publication. But we believe that the reviewers may understand the possible effect of p3-Alcβ peptide in these preliminary animal behavioral analyses. Again, the collaborative study team will progress with various behavior analyses and they will obtain further detailed results in the future. They would like to do a variety of analyses, but that will take more time to complete their studies.

For the argument of overinterpretation in the effects of p3-Alcβ peptides on Aβo-induced cytotoxicity for cultured neurons, we inserted the following sentences on page 5 with the insertion of the unpublished observation 11 into Fig. EV1G; "A higher concentration of Aβo (10 μM) induces stronger neurotoxicity than the low one (2.5 μM). The same concentration (10 μM) of p3-Alcβ peptides partially restored neuronal viability that had been beforehand damaged by Aβo (10 μM) (**Fig. EV1G**). These data indicate that p3-Alcβ37 and p3-Alcβ9-19 protect neurons against Aβo-mediated neurotoxic damage which might be not so severe as it causes cell death."

The PET analysis with [18F]BCPP-EF shows mitochondrial activity which may be one of the criteria for neuronal viability, but mitochondrial dysfunction is common in the brain of AD patients and the current drug such as memantine does not increase mitochondrial activity (Singh *et al*, 2017). The property of p3-Alcβ for AD therapy is likely to be distinct from immunotherapies that use anti-Aβ antibodies, as suggested by other reviewers, which are described in Discussion Section. We believe that the information for the p3-Alcβ properties is beneficial to various researchers in developing a new therapeutic procedure for AD.

14th Mar 2023

Dear Prof. Suzuki,

I am pleased to inform you that your manuscript is accepted for publication and is now being sent to our publisher to be included in the next available issue of EMBO Molecular Medicine.

We would like to remind you that as part of the EMBO Publications transparent editorial process initiative, EMBO Molecular Medicine will publish a Review Process File online to accompany accepted manuscripts. If you do NOT want the file to be published or would like to exclude figures, please immediately inform the editorial office via e-mail.

Please read below for additional IMPORTANT information regarding your article, its publication and the production process.

Congratulations on your interesting work,

Kind regards,
Jingyi

Jingyi Hou
Editor
EMBO Molecular Medicine

Follow us on Twitter @EmboMolMed
Sign up for eTOCs at embopress.org/alertsfeeds

*** *** *** IMPORTANT INFORMATION *** *** ***

SPEED OF PUBLICATION
The journal aims for rapid publication of papers, using using the advance online publication "Early View" to expedite the process: A properly copy-edited and formatted version will be published as "Early View" after the proofs have been corrected. Please help the Editors and publisher avoid delays by providing e-mail address(es), telephone and fax numbers at which author(s) can be contacted.

Should you be planning a Press Release on your article, please get in contact with embomolmed@wiley.com as early as possible, in order to coordinate publication and release dates.

LICENSE AND PAYMENT:

All articles published in EMBO Molecular Medicine are fully open access: immediately and freely available to read, download and share.

EMBO Molecular Medicine charges an article processing charge (APC) to cover the publication costs. You, as the corresponding author for this manuscript, should have already received a quote with the article processing fee separately. Please let us know in case this quote has not been received.

Once your article is at Wiley for editorial production you will receive an email from Wiley's Author Services system, which will ask you to log in and will present you with the publication license form for completion. Within the same system the publication fee can be paid by credit card, an invoice, pro forma invoice or purchase order can be requested.

Payment of the publication charge and the signed Open Access Agreement form must be received before the article can be published online.

PROOFS

You will receive the proofs by e-mail approximately 2 weeks after all relevant files have been sent o our Production Office. Please return them within 48 hours and if there should be any problems, please contact the production office at embopressproduction@wiley.com.

Please inform us if there is likely to be any difficulty in reaching you at the above address at that time. Failure to meet our deadlines may result in a delay of publication.

All further communications concerning your paper proofs should quote reference number EMM-2022-17052-V3 and be directed to the production office at embopressproduction@wiley.com.

Thank you,

Jingyi Hou
Editor
EMBO Molecular Medicine

EMBO Press Author Checklist

Corresponding Author Name: Toshiharu Suzuki	
Journal Submitted to: EMBO Molecular Medicine	
Manuscript Number: EMM-2022-17052-V2	

USEFUL LINKS FOR COMPLETING THIS FORM
[The EMBO Journal - Author Guidelines]
[EMBO Reports - Author Guidelines]
[Molecular Systems Biology - Author Guidelines]
[EMBO Molecular Medicine - Author Guidelines]

Reporting Checklist for Life Science Articles (updated January 2022)
This checklist is adapted from Materials Design Analysis Reporting (MDAR) Checklist for Authors. MDAR establishes a minimum set of requirements in transparent reporting in the life sciences (see Statement of Task: 10.31222/osf.io/9sm4x). Please follow the journal's guidelines in preparing your manuscript.
Please note that a copy of this checklist will be published alongside your article.

Abridged guidelines for figures
1. Data
The data shown in figures should satisfy the following conditions:
- the data were obtained and processed according to the field's best practice and are presented to reflect the results of the experiments in an accurate and unbiased manner.
- ideally, figure panels should include only measurements that are directly comparable to each other and obtained with the same assay.
- plots include clearly labeled error bars for independent experiments and sample sizes. Unless justified, error bars should not be shown for technical replicates.
- if n<5, the individual data points from each experiment should be plotted. Any statistical test employed should be justified.
- Source Data should be included to report the data underlying figures according to the guidelines set out in the authorship guidelines on Data Presentation.

2. Captions
Each figure caption should contain the following information, for each panel where they are relevant:
- a specification of the experimental system investigated (eg cell line, species name).
- the assay(s) and method(s) used to carry out the reported observations and measurements.
- an explicit mention of the biological and chemical entity(ies) that are being measured.
- an explicit mention of the biological and chemical entity(ies) that are altered/varied/perturbed in a controlled manner.
- the exact sample size (n) for each experimental group/condition, given as a number, not a range;
- a description of the sample collection allowing the reader to understand whether the samples represent technical or biological replicates (including how many animals, litters, cultures, etc.).
- a statement of how many times the experiment shown was independently replicated in the laboratory.
- definitions of statistical methods and measures:
  - common tests, such as t-test (please specify whether paired vs. unpaired), simple χ^2 tests, Wilcoxon and Mann-Whitney tests, can be unambiguously identified by name only, but more complex techniques should be described in the methods section;
  - are tests one-sided or two-sided?
  - are there adjustments for multiple comparisons?
  - exact statistical test results, e.g., P values = x but not P values < x;
  - definition of 'center values' as median or average;
  - definition of error bars as s.d. or s.e.m.

Please complete ALL of the questions below.
Select "Not Applicable" only when the requested information is not relevant for your study.

Materials

Newly Created Materials	Information included in the manuscript?	In which section is the information available? (Reagents and Tools Table, Materials and Methods, Figures, Data Availability Section)
New materials and reagents need to be available; do any restrictions apply?	Not Applicable	

Antibodies	Information included in the manuscript?	In which section is the information available? (Reagents and Tools Table, Materials and Methods, Figures, Data Availability Section)
For **antibodies** provide the following information:<br>- Commercial antibodies: RRID (if possible) or supplier name, catalogue number and or/clone number<br>- Non-commercial: RRID or citation	Yes	Commercial antibodies were described in Appendix Table S2. Noncommercial antibodies are described in Materials and Methods section (page 11)

DNA and RNA sequences	Information included in the manuscript?	In which section is the information available? (Reagents and Tools Table, Materials and Methods, Figures, Data Availability Section)
**Short novel DNA or RNA including primers, probes**: provide the sequences.	Not Applicable	

Cell materials	Information included in the manuscript?	In which section is the information available? (Reagents and Tools Table, Materials and Methods, Figures, Data Availability Section)
**Cell lines:** Provide species information, strain. Provide accession number in repository **OR** supplier name, catalog number, clone number, and/**OR** RRID.	Not Applicable	
**Primary cultures:** Provide species, strain, sex of origin, genetic modification status.	Yes	Mouse primary cultured neurons are described in Materials and Methods (pages 10-11)
Report if the cell lines were recently **authenticated** (e.g., by STR profiling) and tested for mycoplasma contamination.	Not Applicable	

Experimental animals	Information included in the manuscript?	In which section is the information available? (Reagents and Tools Table, Materials and Methods, Figures, Data Availability Section)
**Laboratory animals or Model organisms:** Provide species, strain, sex, age, genetic modification status. Provide accession number in repository **OR** supplier name, catalog number, clone number, **OR** RRID.	Yes	Laboratory animals were described in Materials and Method (pages 10-11)
**Animal observed in or captured from the field:** Provide species, sex, and age where possible.	Not Applicable	
Please detail **housing and husbandry conditions**.	Yes	The conditions are described in Materials and Methods (page 10)

Plants and microbes	Information included in the manuscript?	In which section is the information available? (Reagents and Tools Table, Materials and Methods, Figures, Data Availability Section)
**Plants:** provide species and strain, ecotype and cultivar where relevant, unique accession number if available, and source (including location for collected wild specimens).	Not Applicable	
**Microbes:** provide species and strain, unique accession number if available, and source.	Not Applicable	

Human research participants	Information included in the manuscript?	In which section is the information available? (Reagents and Tools Table, Materials and Methods, Figures, Data Availability Section)
If collected and within the bounds of privacy constraints report on age, sex and gender or ethnicity for all study participants.	Not Applicable	

Core facilities	Information included in the manuscript?	In which section is the information available? (Reagents and Tools Table, Materials and Methods, Figures, Data Availability Section)
If your work benefited from core facilities, was their service mentioned in the acknowledgments section?	Not Applicable	

Design

Study protocol	Information included in the manuscript?	In which section is the information available? (Reagents and Tools Table, Materials and Methods, Figures, Data Availability Section)
If study protocol has been **pre-registered, provide DOI in the manuscript**. For clinical trials, provide the trial registration number **OR** cite DOI.	Not Applicable	
Report the **clinical trial registration number** (at ClinicalTrials.gov or equivalent), where applicable.	Not Applicable	

Laboratory protocol	Information included in the manuscript?	In which section is the information available? (Reagents and Tools Table, Materials and Methods, Figures, Data Availability Section)
Provide DOI OR other citation details if **external detailed step-by-step protocols** are available.	Not Applicable	

Experimental study design and statistics	Information included in the manuscript?	In which section is the information available? (Reagents and Tools Table, Materials and Methods, Figures, Data Availability Section)
Include a statement about **sample size** estimate even if no statistical methods were used.	Not Applicable	
Were any steps taken to minimize the effects of subjective bias when allocating animals/samples to treatment (e.g. **randomization procedure**)? If yes, have they been described?	Not Applicable	
Include a statement about **blinding** even if no blinding was done.	Not Applicable	
Describe **inclusion/exclusion criteria** if samples or animals were excluded from the analysis. Were the criteria pre-established?<br><br>If sample or data points were omitted from analysis, report if this was due to attrition or intentional exclusion and provide justification.	Not Applicable	
For every figure, are **statistical tests** justified as appropriate? Do the data meet the assumptions of the tests (e.g., normal distribution)? Describe any methods used to assess it. Is there an estimate of variation within each group of data? Is the variance similar between the groups that are being statistically compared?	Yes	Statistical analysis was described on page 17, and also the details are described in figure legends.

Sample definition and in-laboratory replication	Information included in the manuscript?	In which section is the information available? (Reagents and Tools Table, Materials and Methods, Figures, Data Availability Section)
In the figure legends: state number of times the experiment was **replicated** in laboratory.	Yes	In figure legends.
In the figure legends: define whether data describe **technical or biological replicates**.	Not Applicable	

Ethics

Ethics	Information included in the manuscript?	In which section is the information available? (Reagents and Tools Table, Materials and Methods, Figures, Data Availability Section)
Studies involving **human participants**: State details of **authority granting ethics approval** (IRB or equivalent committee(s), provide reference number for approval.	Yes	Cohort information and quantification of CSF biomarkers. (page 17)
Studies involving **human participants**: Include a statement confirming that **informed consent** was obtained from all subjects and that the experiments conformed to the principles set out in the WMA Declaration of Helsinki and the Department of Health and Human Services Belmont Report.	Yes	Cohort information and quantification of CSF biomarkers. (page 17) Also in the original publications for CSF collection (Kasuga et al 2022) which is cited in page 17.
Studies involving **human participants:** For publication of **patient photos**, include a statement confirming that consent to publish was obtained.	Not Applicable	
Studies involving experimental **animals**: State details of **authority granting ethics approva**l (IRB or equivalent committee(s), provide reference number for approval. Include a statement of compliance with ethical regulations.	Yes	Animals and mouse primary cultured neurons. (page 10)
Studies involving **specimen and field samples:** State if relevant **permits** obtained, provide details of authority approving study; if none were required, explain why.	Not Applicable	

Dual Use Research of Concern (DURC)	Information included in the manuscript?	In which section is the information available? (Reagents and Tools Table, Materials and Methods, Figures, Data Availability Section)
Could your study fall under dual use research restrictions? Please check biosecurity documents and list of **select agents and toxins** (CDC): https://www.selectagents.gov/sat/list.htm	Not Applicable	
If you used a select agent, is the security level of the lab appropriate and reported in the manuscript?	Not Applicable	
If a study is subject to dual use research of concern regulations, is the name of the **authority granting approval and reference number** for the regulatory approval provided in the manuscript?	Not Applicable	

Reporting

The MDAR framework recommends adoption of discipline-specific guidelines, established and endorsed through community initiatives. Journals have their own policy about requiring specific guidelines and recommendations to complement MDAR.

Adherence to community standards	Information included in the manuscript?	In which section is the information available? (Reagents and Tools Table, Materials and Methods, Figures, Data Availability Section)
State if relevant guidelines or checklists (e.g., **ICMJE, MIBBI, ARRIVE, PRISMA**) have been followed or provided.	Yes	All animal studies were conducted in compliance with the ARRIVE guidelines. (page 10)
For **tumor marker prognostic studies,** we recommend that you follow the **REMARK** reporting guidelines (see link list at top right). See author guidelines, under 'Reporting Guidelines'. Please confirm you have followed these guidelines.	Not Applicable	
For **phase II and III randomized controlled trials**, please refer to the **CONSORT** flow diagram (see link list at top right) and submit the CONSORT checklist (see link list at top right) with your submission. See author guidelines, under 'Reporting Guidelines'. Please confirm you have submitted this list.	Not Applicable	

Data Availability

Data availability	Information included in the manuscript?	In which section is the information available? (Reagents and Tools Table, Materials and Methods, Figures, Data Availability Section)
Have **primary datasets** been deposited according to the journal's guidelines (see 'Data Deposition' section) and the respective accession numbers provided in the Data Availability Section?	Not Applicable	
Were **human clinical and genomic datasets** deposited in a public access-controlled repository in accordance to ethical obligations to the patients and to the applicable consent agreement?	Not Applicable	
Are **computational models** that are central and integral to a study available without restrictions in a machine-readable form? Were the relevant accession numbers or links provided?	Not Applicable	
If publicly available data were reused, provide the respective **data citations in the reference list**.	Not Applicable	
